# Dental Implant Systems

**DOI:** 10.3390/ijms11041580

**Published:** 2010-04-12

**Authors:** Yoshiki Oshida, Elif B. Tuna, Oya Aktören, Koray Gençay

**Affiliations:** 1 Department of Mechanical and Aerospace Engineering, Syracuse University, Syracuse NY 13244, USA; 2 Department of Pedodontics, Faculty of Dentistry, Istanbul University 34093, Capa Istanbul, Turkey

**Keywords:** titanium materials, dental implant system, compatibility, surface engineering, gradual functional materials

## Abstract

Among various dental materials and their successful applications, a dental implant is a good example of the integrated system of science and technology involved in multiple disciplines including surface chemistry and physics, biomechanics, from macro-scale to nano-scale manufacturing technologies and surface engineering. As many other dental materials and devices, there are crucial requirements taken upon on dental implants systems, since surface of dental implants is directly in contact with vital hard/soft tissue and is subjected to chemical as well as mechanical bio-environments. Such requirements should, at least, include biological compatibility, mechanical compatibility, and morphological compatibility to surrounding vital tissues. In this review, based on carefully selected about 500 published articles, these requirements plus MRI compatibility are firstly reviewed, followed by surface texturing methods in details. Normally dental implants are placed to lost tooth/teeth location(s) in adult patients whose skeleton and bony growth have already completed. However, there are some controversial issues for placing dental implants in growing patients. This point has been, in most of dental articles, overlooked. This review, therefore, throws a deliberate sight on this point. Concluding this review, we are proposing a novel implant system that integrates materials science and up-dated surface technology to improve dental implant systems exhibiting bio- and mechano-functionalities.

## 1. Introduction

The goal of modern dentistry is to restore the patient to normal function, speech, health and aesthetics, regardless of the atrophy, disease, or injury of the stomatognathic system. Responding to this ultimate goal, dental implants are an ideal option for people in good general oral health who have lost a tooth (or teeth) due to periodontal disease, an injury, or some other reasons. Dental implants (considered as an artificial tooth root) are biocompatible metal anchors surgically positioned in the jaw bone (in other words, surgically traumatized bone) underneath the gums to support an artificial crown where natural teeth are missing. Using the root form implants (the closest in shape and size to the natural tooth root), the non-union (due to traumatization) bone healing period usually varies from as few as three months to six or more. During this period, osseointegration occurs. The bone grows in and around the implant creating a strong structural support, to which a superstructure will be attached later on by either cemetation or screw-tightening retaining technique. Today, titanium matrials (varied from commercially pure titanium ASTM Grades 1 through 4 or Ti-based alloys) are considered to be the most biologically compatible materials to vital tissue. Titanium materials are used preferentially in many of the more recent applications in maxillofacial, oral, neuro and cardiovascular-surgery, as well as gaining increasing preference in orthopedics. These facts indicate a superiority of titanium materials. Moreover, they have been successfully used for orthopedic and dental implants. Direct bony interface promised more longevity than previously used systems; hence, oral implantology gained significant additional momentum.

Following the introduction of concept and practice of the osseintegration into restorative dentistry in the early1960s [1], completely edendulous mandibular arches in elderly patients received primary emphasis regarding the restoration of oral function. Following excellent long-term results in the treatment of completely edentulous arches, implant-supported fixed partial dentures and overdentures became common treatment modalities. The number of dental implants used in the United States ever increased an estimated four-fold from 1983 to 1987 [2], and it further increased 75% between 1986 and 1990 [3]. At the beginning of this century, it was reported that there were 25 dental implants manufacturers with marketing about 100 different dental implant systems with variety of diameters, lengths, surfaces, platforms, interfaces, and body shapes [4]. Significant differentiation and distinctions are based on (i) the implant/abutment interface, (ii) the body shape, and (iii) the implant-to-bone surface. This remarkable increased need and use of implant-treatments may result from the combined effect of a number of factors; including (1) aging population, (2) tooth loss related to age, (3) anatomic consequences of edentulism, (4) poor performance of removable prostheses, (5) psychologic aspects of tooth loss, (6) predictable long-term results of implant-supported prostheses, and (7) advantages of implant-supported prostheses [5].

Many aspects of biocompatibility profiles established for dental implants have been shown to depend on interrelated biomaterials, tissue, and host factors, being associated with either surface and bulk properties. In general, the biomaterial surface chemistry (purity and surface tension for wetting), topography (roughness), and type of tissue integration (osseous, fibrous, or mixed) can be correlated with shorter and longer term *in vivo* host responses. Additionally, the host environment has been shown directly influence the biomaterial-to-tissue interface zone specific to the local biochemical and biomechanical circumstances of healing and longer term clinical aspects of load-bearing function. The interaction at interface between recipient tissues and implanted material are limited to the surface layer of the implant and a few nanometers into the living tissues. The details of the interaction (hard or soft tissue) and force transfer that results in static (stability) or dynamic (instability or motion) conditions have also been shown to significantly alter the clinical longevities of intraoral device constructs [6].

In this review, several important requirements for successful dental implant systems will be firstly reviewed, and followed by variety of surface modifications and technology to accommodate the biological interaction at the interface between placed implant and receiving vital tissue. Within an increased predictability of dental implants, the same treatment modalities have come under consideration for growing patients [7]. However, there are special issues taken into account, due to growing hard tissue. Although extensive reviews on implants have been previously published [6,8,9], no integrated concerns can be found on dental implantology in growing patients. Accordingly, we added some special and uniqueness to this review with implant practices in growing patients.

## 2. Requirements for Successful Implant Systems

### 2.1. Safety Concerns

Safety concerns should not be limited to dental implants, but also to all dental devices. Specifications and standards have been developed to aid producers, users, and consumers in the evaluation of the safety and effectiveness of dental products. However, the decision of products to test their materials according to national and international standards is purely voluntary [10]. Until the passage in 1976 of the Medical Device Amendments to the Food and Drug Act, medical and dental materials and devices for use in the human were not regulated by any agency of the United States government. The only exception was materials for which therapeutic claims were made, which allowed the Food and Drug Administration (FDA) to consider them as a drug. The Medical Device Amendments of 1976 gave the FDA jurisdiction over all materials, devices, and instruments used in the diagnosis, cure, mitigation, treatment, or prevention of disease in man. This includes materials used professionally and the over-the-counter products sold directly to the public. The Dental Panel places an item one of three classes: Class I, materials posing minimum risk: these are subject only to good manufacturing and recordkeeping procedures. Class II, materials for which safety and efficacy needs to be demonstrated and for which performance standards are available: materials must be shown to meet the performance standard. Class III, materials that pose significant risk and materials for which performance standards have not been formulated: this class is subject to premarket approval by the FDA for safety and efficacy, in much the same manner as a new drug [11].

According to ISO specifications [12], implant devices are required to evaluate several tests; for Group I tests (cytotoxicity tests: ISO 7405, 6.1 and 6.2, and cytotoxicity tests: ISO 10993.5), for Group II tests (subchronic systemic toxicity – Oral application: ISO 10993.11.6.7.1, skin irritation and intracutaneous reactivity: ISO 10993-11.5.2, and sensitization systemic toxicity–application by inhalation: ISO 10993.11.6.7.3, genotoxicity: ISO 10993.3, and local effects after implantation: ISO 10993.6), and for Group III (pulp cappling and pulpotomy: ISO 7405.6.4, and endodontic usage test: ISO 7405.6.5).

### 2.2. Compatibility

The implantation of devices for the maintenance or restoration of a body function imposes extraordinary requirements on the materials of construction. Foremost among these is an issue of biocompatibility. There are interactions between the foreign material and the surrounding host living tissue, fluid and blood elements. Some of these are simply adaptive. Others constitute hazard, both short and long term, to the survival of the living system [13,14]. There are mechanical and physical properties which the material must provide, and structural nature which the system should exhibit. Some of these govern the ability of the device to provide its intended function from an engineering viewpoint. Others such as tribology (specifically friction and wear), corrosion and mechanical compliance relate significantly to the biocompatibility concerns. Human implantation applications impose more stringent requirements on reliability than any other engineering task. In most applications an implanted device is expected to function for the life of the patient. As the medical profession becomes more emboldened, the device lifetime must stretch to more than 30 years (if follow-up maintenance is carefully and thoroughly performed and excellent coorperation from patients is obtained), and there are very few engineering devices which have been designed to function for more than 30 years. It is necessary to think in terms of performance reliability of thousands of devices for the lifetime of a patient and a tolerable expectation of failure of perhaps no more than one in one thousand [14,15]. One of many universal requirements of implants, wherever they are used in the body, is the ability to form a suitably stable mechanical unit with the neighboring hard or soft tissues. A loose (or unstable) implant may function less efficiently or cease functioning completely, or it may induce an excessive tissue response. In either case, it may cause the patient discomfort and pain.

There are at three least major required compatibilities for placed implants to exhibit biointegration to receiving hard tissue and biofucntionality thereafter. They include (1) biological compatibility, (2) mechanical compatibility, and (3) morphological compatibility to receiving host tissues [16,17].

#### 2.2.1. Biological Compatibility (Biocompatibility)

The service conditions in the mouth are hostile, both corrosively and mechanically. All intraorally placed parts are continuously bathed in saliva, an aerated aqueous solution of about 0.1 N chlorides, with varying amounts of Na, K, Ca, PO_4_, CO_2_, sulphur compounds and mucin [18]. The pH value is normally in the range of 5.5 to 7.5, but under plaque deposits it can be as low as 2. Temperatures can vary ±36.5 °C, and a variety of food and drink concentrations apply for short periods. Loads may be up to 1,000 N (with normal masticatory force ranging from 150 N to 250 N) [18], sometimes at an impact-load superimposed. Trapped food debris may decompose to create sulphur compounds, causing placed devices discoloration [18]. With such hostile conditions, biocompatibility of metallic materials essentially equates to corrosion resistance because it is thought that alloying elements can only enter the surrounding organic system and develop toxic effects by conversion to ions through chemical or electrochemical process.

As mentioned before, the interface zone between placed implant and receiving hard/soft tissue is strongly governing the success and longevity of placed implant. Hence, there are two major research approaches; one is looking at the interface from foreign implant material side, the other is from vital tissue side. Although this review’s principle scope is aiming at the material’s side, it would be worth to take a brief look at what is happening right after the placing the implant surgically at vital traumatized hard tissue.

After implant placement, initial healing of the bony compartment is characterized by formation of blood clots at the traumatized wound site, protein adsorption and adherence of polymorphonuclear leukocyte [19]. Then approximately two days after placement of the implant, fibroblasts proliferate into the blood clot, organization begins, and an extra-cellular matrix is produced. Approximately one week after the implant is placed, appearance of osteblast-like cells and new bone is seen. New bone reaches the implant surface by osseoconduction (through growth of bone over the surface and migration of bone cells over the implant surfaces) [19].

Why do titanium and its alloys show such good biocompatibility compared with other alloys? The answer to this question is generally that titanium is passive in aqueous solutions, and the passive film that forms on titanium is stable, even in a biological system including chemical and mechanical environments. Such an interpretation is true in many cases. However, the presence of the passive film is only part of the answer when we consider the complex interfacial phenomena to be found between titanium and a biological system, in both biological and biomechanical environments [20,21]. There are certain criteria for any potential metallic materials to be evaluate excellent corrosion resistant, including (1) ease to be oxidized, (2) strong adherence of formed oxide to the substrate, (3) dense of formed oxide, and (4) protectiveness of formed oxide. The Pilling –Bedworth (P-B) ratio is the very simple indication to judge whether the formed oxide is protective or not [22]. If P-B ration is less than 1, since oxide occupies small volume than the metal, so that formed oxide is porous and non-protective. If it is larger than 2, since oxide occupies a large volume and may flake from the surface, exposing fresh substrate surface and again exhibits non-protectiveness. If P-B ration is between 1 and 2, the volume of oxide is similar to that of metal, so that the formed oxide is adherent to substrate, nonporous, and protective. It was calculated that P-B ratio for TiO_2_ formation is 1.76, indicating that the formed TiO_2_ is protective. Titanium is a highly reactive metal and will react within microseconds to form an oxide layer when exposed to the atmosphere [23]. Although the standard electrode potential was reported in a range from −1.2 to −2.0 volts for the Ti ↔ Ti^3+^ electrode reaction [24], due to strong chemical affinity to oxygen, it easily produces a compact oxide film, ensuring high corrosion resistance of the metal. This oxide, which is primarily TiO_2_, forms readily because it has one of the highest heats of reaction known (ΔH = −915 kJ/mole) (for 298.16°–2,000°K) [25]. It is also quite impenetrable to oxygen (since the atomic diameter of Ti is 0.29 nm, the primary protecting layer is only about 5 to 20 atoms thick) [26]. The formed oxide layer adheres strongly to the titanium substrate surface. The average single-bond strength of the TiO_2_ to Ti substrate was reported to be about 300 kcal/mol, while it is 180 kcal/mol for Cr_2_O_3_/Cr, 320 kcal/mol for Al_2_O_3_/Al, and 420 kcal/mol for both Ta_2_O_5_/Ta and Nb_2_O_5_/Nb [27].

During implantation, titanium releases corrosion products (which is mainly titanium oxide or titanium hydro-oxide) into the surrounding tissue and fluids even though it is covered by a thermodynamically stable oxide film [28,29]. An increase in oxide thickness, as well as incorporation of elements from the extra- cellular fluid (P, Ca, and S) into the oxide, has been observed as a function of implantation time [30]. Moreover, changes in the oxide stoichiometry, composition, and thickness have been associated with the release of titanium corrosion products *in vitro* [31]. Properties of the oxide, such as stoichiometry, defect density, crystal structure and orientation, surface defects, and impurities were suggested as factors determining biological performance [32,33].

The performance of titanium and its alloys in surgical implant applications can be evaluated with respect to their biocompatibility and capability to withstand the corrosive species involved in fluids within the human body [34]. This may be considered as an electrolyte in an electrochemical reaction. It is well documented that the excellent corrosion resistance of titanium materials is due to the formation of a dense, protective, and strongly-adhered film – which is called a passive film, as discussed before. Such a surface situation is referred to passivity or a passivation state. The exact composition and structure of the passive film covering titanium and its alloys is controversial. This is the case not only for the “natural” air oxide, but also for films formed during exposure to various solutions, as well as those formed anodically. The “natural” oxide film on titanium ranges in thickness from 2 to 7 nm, depending on such parameters as the composition of the metal and surrounding medium, the maximum temperature reached during the working of the metal, the surface finish, *etc*.

Oxides formed on Ti materials are varied with a general form; TiO_X_ (1 < x < 2). Depending on x values, there are five different crystalline oxides; *i.e.*, (1) cubic TiO (a_o_ = 4.24 Å), (2) hexagonal Ti_2_O_3_ (a_o_ = 5.37 Å, α = 56°48’), (3) tetragonal TiO_2_ (anatase) (a_o_ = 3.78Å, c_o_ = 9.50 Å), (4) tetragonal TiO_2_ (rutile) (a_o_ = 4.58 Å, c_o_ = 2.98 Å), and (5) orthorhombic TiO_2_ (brookite) (a_o_ = 9.17 Å, b_o_ = 5.43 Å, c_o_ = 5.13 Å). Besides these, there are (6) non-stoichiometric oxide (when x is not integral), and (7) amorphous oxides. It is widely believed that, among these oxides, only rutile and anatase type oxides are stable at normal conditions. Of interest, choice for rutile formation or anatase formation depends on the acidity of used electrolyte [8]. The rutile and anatase type oxides exibit different physical properties – interms of surface tension. Lim *et al*. [35] prepared various surface conditions on pure titanim and measured surface contact angles, surface electrochemical potential and roughness. It was found that the surface covered with only rutile type TiO_2_ was hydrophobic, whereas that covered with a mixture of rutile and anatase type of oxides showed hydrophilicity [35].

The level of neutrophil priming and activation following implant placement may be linked to the development and maintenance of long-term stability and osseointegration. Bisphosphonate effect on neutrophil activation was examined on differently treated surfaces [36]. Neutrophils were isolated from whole blood collected from healthy human donors, on a double dextran gradient. Treated surfaces were incubated with 5 × 10^5^ neutrophils per curette. Luminol-dependent CL (chemiluminescence) was recorded for 60 min (priming or inflammatory phase), followed by secondary stimulation with 10^−7^ M phorbol myrisitate acetate at 60 min (activation phase) for a continuous CL measurement over 120 min. SEM evaluation was preformed. Results indicated that titanium surfaces which were covered with a mixture of rutile and anatase type TiO_2_ oxide films are capable of priming neutrophils, when compared to the acid-treated surface which was covered with rutile oxide only [36].

Using Auger Electron Spectroscopy (AES) to study the change in the composition of the titanium surface during implantation in human bone, observed that the oxide formed on titanium implants grows and takes up minerals during the implantation [30,37]. The growth and uptake occur even though the adsorbed layer of protein is present on the oxide, indicating that mineral ions pass through the adsorbed protein. It was shown that, using Fourier Transform Infrared Reflection Absorption Spectroscopy (FTIR-RAS), phosphate ions are adsorbed by the titanium surface after the protein has been adsorbed. Using x-ray photoelectron spectroscopy (XPS) [38], it was demonstrated that oxides on commercially pure titanium and titanium alloy (Ti-6Al-4V) change into complex phosphates of titanium and calcium containing hydroxyl groups which bind water on immersion in artificial saliva (pH = 5.2) [39]. It was shown that titanium is in almost direct contact to bone tissue, separated only by an extremely thin cell-free non-calcified tissue layer. Transmission electron microscopy revealed an interfacial hierarchy that consisted of a 20–40 nm thick proteoglycan layer within 4 nm of the titanium oxide, followed by collagen bundles as close as 100 nm and Ca deposits within 5 nm of the surface [40]. To reach the steady-state interface described, both the oxide on titanium and the adjacent tissue undergo various reactions. The physiochemical properties of titanium have been associated with the unique tissue response to the materials: these include the biochemistry of released corrosion products, kinetics of release and the oxide stoichiometry, crystal defect density, thickness and surface chemistry [41]. All these studies indicate that the surface oxide on titanium materials reacts with mineral ions, water, and other constituents of biofluids, and that these reactions, in turn, cause a remodeling of the surface.

As seen in the above, in general, the titanium passivating layer not only produces good corrosion resistance, but it seems also to allow physiological fluids, proteins, and hard and soft tissue to come very close and/or deposit on it directly. Reasons for this are still largely unknown, but it may have something to do with things such as the high dielectric constant for TiO_2_ (50 to 170 *vs*. 4–10 for alumina and dental porcelain), which should result in considerably stronger van der Waal’s bonds on TiO_2_ than other oxides; TiO_2_ may be catalytically active for a number of organic and inorganic chemical interactions influencing biological processes at the implant interface. The TiO_2_ oxide film may permit a compatible layer of biomolecule to attach [42,43].

#### 2.2.2. Mechanical Compatibility

Biomechanics involved in implantology should include at least (1) the nature of the biting forces on the implants, (2) transferring of the biting forces to the interfacial tissues, and (3) the interfacial tissues reaction, biologically, to stress transfer conditions. Interfacial stress transfer and interfacial biology represent more difficult, interrelated problems. While many engineering studies have shown that variables such as implant shape, elastic modulus, extent of bonding between implant and bone *etc*., can affect the stress transfer conditions, the unresolved question is whether there is any biological significance to such differences. The successful clinical results achieved with osseointegrated dental implants underscore the fact that such implants easily withstand considerable masticatory loads. In fact, it was reported that bite forces in patients with these implants were comparable to those in patients with natural dentitions. A critical aspect affecting the success or failure of an implant is the manner in which mechanical stresses are transferred from the implant to bone smoothly [44]. It is essential that neither implant nor bone be stressed beyond the long-term fatigue capacity. It is also necessary to avoid any relative motion that can produce abrasion of the bone or progressive loosening of the implants. An osseointegrated implant provides a direct and relatively rigid connection of the implant to the bone. This is an advantage because it provides a durable interface without any substantial change in form or duration. There is a mismatch of the mechanical properties at the interface of Ti and bone. It is interesting to observe that from a mechanical standpoint, the shock-absorbing action would be the same if the soft layer were between the metal implant and the bone. In the natural tooth, the periodontum, which forms a shock-absorbing layer, is in this position between the tooth and jaw bone [44]. Natural teeth and implants have different force transmission characteristics to bone. Compressive strains were induced around natural teeth and implants as a result of static axial loading, whereas combinations of compressive and tensile strains were observed during lateral dynamic loading [8,9]. Magnitude of strain around the natural tooth is significantly lower than the opposing implant and occluding implants in the contra lateral side for most regions under all loading conditions. It was repoted that there was a general tendency for increased strains around the implant opposing natural tooth under higher loads and particularly under lateral dynamic loads [45].

By means of finite element (FEM) analysis, stress-distribution in bone around implants was calculated with and without stress-absorbing element [46]. A freestanding implant and an implant connected with a natural tooth were simulated. For the freestanding implant, it was reported that the variation in the modulus of elasticity of the stress-absorbing element had no effect on the stresses in bone. Changing the shape of the stress-absorbing element had little effect on the stresses in cortical bone. For the implant connected with a natural tooth, it was concluded that (i) a more uniform stress was obtained around the implant with a low modulus of elasticity of the stress-absorbing element, and (ii) the bone surrounding the natural tooth showed a decrease in the height of the peak stresses.

The dental or orthopedic prostheses, particularly the surface zone thereof, should respond to the loading transmitting function. The placed implant and receiving tissues establish a unique stress-strain field. Between them, there should be an interfacial layer. During the loading with implant/bone couple, the strain-field continuity should be held (if not, it should indicate that implant is not fused to vital bone), although the stress-field is obviously in a discrete manner due to different values of modulus of elasticity (E) between host tissue and foreign implant material. Namely, stress at bone σ_B_ = E_B_ɛ_B_ and stress at implant σ_I_ = E_I_ɛ_I_. Under the continuous strain field, ɛ_B_ = ɛ_I_. However E_B_ ≠ E_I_ due to dissimilar material couple condition. If the magnitude of the difference in modulus of elasticity is large, then the interfacial stress, accordingly, could become so large that the placed implant system will face a risky failure or detachment situation. In other words, if interfacial stress due to stress difference Δσ = (σ_I_–σ_B_) is larger than the osteointegrated fused implant retention strength, the placed impant will be failed. Therefore, materials for implant or surface zone of implants should be mechanically compatible to mechanical properties (especially, modulus of elasticity) of receiving tissues to minimize the interfacial discrete stress. This is the second compatibility and is called as the mechanical compatibility [8,16].

**Figure 1.**
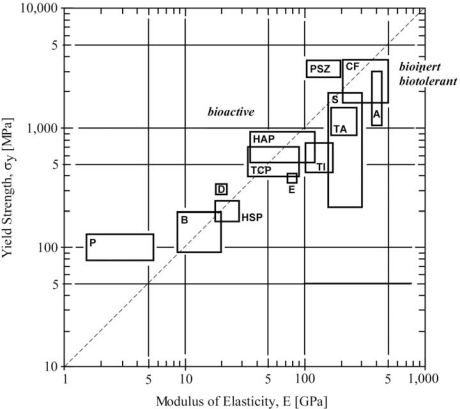
Relationship between yield strength and modulus of elasticity of various biomaterials. P: polymeric materials, B: bone, D: dentin, HSP: high strength polymers (e.g., Kevlar), E: enamel, TCP: tricalcium phosphate, HAP: hydroxyapatite, TI: commercially pure titanium, TA: titanium alloys (e.g., Ti-6Al-4V), S: 304-series stainless steel, PSZ: partially stabilized zirconia, A: alumina, CF: carbon fiber.

Figure 1 shows a relationship between yield strength and modulus of elasticity (in other words, rigidity) of various types of biomaterials and bones which are receiving vital tissue for placed implants. As seen clearly, strength and rigidity of all biomaterials concerned are related linearly on both-log scale. From the point of strain-continuity viewpoint, it is ideal to choose any implantable materials that have both strength and rigidity values close to those of receiving bone. Hydroxyapatite coating onto titanium implant has been widely adopted since the both hydroxyapatite (HA) and receiving vital bone possess similar chemical compositions, hence early adaptation can be highly expected. At the same time, E_HA_ is positioned inbetween the values of E_B_ and E_I_; as a result, HA coating will have a second function for mechanical compatibility to make the stress a smooth transfer (or to minimize the interfacial stress). This is one of the typical hindsight, because HA-coating is originally and still now performing due to its similarity of its chemical composition to receiving bone.

#### 2.2.3. Morphological Compatibility

Surface plays a crucial role in biological interactions for four reasons. First, the surface of a biomaterial is the only part in contact with the bioenvironment. Second, the surface region of a biomaterial is almost always different in morphology and composition from the bulk. Differences arise from molecular rearrangement, surface reaction, and contamination. Third, for biomaterials that do not release nor leak biologically active or toxic substances, the characteristics of the surface govern the biological response. And fourth, some surface properties, such as topography, affect the mechanical stability of the implant/tissue interface [47,48].

In a scientific article [16], it was found that surface morphology of successful implants has an upper and lower limitations in average roughness (1–50 μm) and average particle size (10–500 μm), regardless of types of implant materials (either metallic, ceramics, or polymeric materials). If a particle size is smaller than 10 μm, the surface will be more toxic to fibroblastic cells and have an adverse influence on cells due to their physical presence independent of any chemical toxic effects. If the pore is larger than 500 μm, the surface zone does not maintain sufficient structural integrity because it is too coarse. This is the third compatibility – morphological compatibility [16,17].

It has been shown that preparation methods of implant surface can significantly affect the resultant properties of the surface and subsequently the biological responses that occur at the surface [49–51]. Recent efforts have shown that the success or failure of dental implants can be related not only to the chemical properties of the implant surface, but also its macromorphologic nature [52–55]. From an *in vitro* standpoint, the response of cells and tissues at implant interfaces can be affected by surface topography or geometry on a macroscopic basis [53,55], as well as by surface morphology or roughness on a microscopic level [53,56]. These characteristics undoubtly affect how cells and tissues respond to various types of biomaterials. Of all the cellular responses, it has been suggested that cellular adhesion is considered the most important response necessary for developing a rigid structural and functional integrity at the bone/implant interface [57]. Cellular adhesion alters the entire tissue response to biomaterials [58].

The effect of surface roughness (Ra: 0.320, 0.490, and 0.874 μm) of the titanium alloy Ti-6Al-4V on the short- and long-term response of human bone marrow cells *in vitro* and on protein adsorption was investigated [59]. Cell attachment, cell proliferation, and differentiation (alkaline phosphatase specific activity) were determined. The protein adsorption of bovine serum albumin and fibronectin, from single protein solutions on rough and smooth Ti-6Al-4V surfaces was examined with XPS and radio labeling. It was found that (i) cell attachment and proliferation were surface roughness sensitive, and increased as the roughness of Ti-6Al-4V increased, (ii) human albumin was adsorbed preferentially onto the smooth substratum, and (iii) the rough substratum bound a higher amount of total protein (from culture medium supplied with 15% serum) and fibronectin (10-fold) than did the smooth one [59], suggesting an importance of the rugophilicity.

Events leading to integration of an implant into bone, and hence determining the long-time performance of the device, take place largely at the interface formed between the tissue and the implant [60]. The development of this interface is complex and is influenced by numerous factors, including surface chemistry and surface topography of the foreign material [61–65]. For example, Oshida *et al*. treated NiTi by acid-pickling in HF-HNO_3_-H_2_O (1:1:5 by volume) at room temperature for 30 seconds to control the surface topology and selectively dissolve Ni, resulting in a Ti-enriched surface layer [66], demonstrating that surface topology can be easily controlled.

The role of surface roughness on the interaction of cells with titanium model surfaces of well-defined topography was investigated using human bone-derived cells (MG63 cells). The early phase of interactions was studied using a kinetic morphological analysis of adhesion, spreading, and proliferation of the cells. SEM and double immuno-fluorescent labeling of vinculin and actin revealed that the cells responded to nanoscale roughness with a higher cell thickness and a delayed apparition of the focal contacts. A singular behavior was observed on nanoporous oxide surfaces, where the cells were more spread and displayed longer and more numerous filopods. On electrochemically micro-structured surfaces, the MG63 cells were able to penetrate inside, adhere, and proliferate in cavities of 30 or 100 μm in diameter, whereas they did not recognize the 10 μm diameter cavities. Cells adopted a 3D shape when attaching inside the 30 μm diameter cavities. It was concluded that nanotopography on surfaces with 30 μm diameter cavities had little effect on cell morphology compared to flat surfaces with the same nanostructure, but cell proliferation exhibited a marked synergistic effect of microscale and nanoscale topography [67].

On a macroscopic level (roughness > 10 μm) roughness influences the mechanical properties of the titanium/bone interface, the mechanical interlocking of the interface, and the biocompatibility of the material [68,69]. Surface roughness in the range from 10 nm to 10 μm may also influence the interfacial biology, since it is the same order as the size of the cells and large biomolecules [23]. Microroughness at this level includes material defects, such as grain boundaries, dislocation steps and kinks, and vacancies that are active sites for adsorption, and therefore influence the bonding of biomolecules to the implant surface [70]. Microrough surfaces promote significantly better bone apposition than smooth surfaces, resulting in a higher percentage of bone in contact with the implant. Microrough surfaces may influence the mechanical properties of the interface, stress distribution, and bone remodeling [71]. Increased contact area and mechanical inter-locking of bone to a microrough surface can decrease stress concentrations resulting in decreased bone resorption. Bone resorption takes place shortly after loading smooth surfaced implants [72], resulting in a fibrous connective tissue layer, whereas remodeling occurs on rough surfaces [73].

Recently developed clinical oral implants have been focused on topographical changes of implant surfaces, rather than alterations of chemical properties [55,74–77]. These attempts may have been based on the concept that mechanical interlocking between tissue and implant materials relies on surface irregularities in the nanometer to micron level. Recently published *in vivo* investigations have shown significantly improved bone tissue reactions by modification of the surface oxide properties of Ti implants [78–85]. It was found that in animal studies, bone tissue reactions were strongly reinforced with oxidized titanium implants, characterized by a titanium oxide layer thicker than 600 nm, a porous surface structure, and an anatase type of Ti oxide with large surface roughness compared with turned implants [83,84]. This was later supported by work done by Lim *et al*. [35], Oshida and [86], and Elias *et al*. [87] who found that the alkali-treated CpTi surface was covered mainly with anatase type TiO_2_, and exhibited hydrophilicity, whereas the acid-treated CpTi was covered with rutile type TiO_2_ with hydrophobicity. Besides this characteristic crystalline structure of TiO_2_, it was mentioned that good osseointegration, bony apposition, and cell attachment of Ti implant systems [28,88,89] are partially due to the fact that the oxide layer, with unusually high dielectric constant of 50–170, depending on the TiO_2_ concentration, may be the responsible feature [23,42,43].

### 2.3. MRI Safety and Image Compatibility

Magnetic resonance imaging (MRI) is a technology developed in medical imaging that is probably the most innovative and revolutionary other than computed tomography. MR is a three-dimensional imaging technique used to image the protons of the body by employing magnetic fields, radio frequencies, electromagnetic detectors, and computers [90]. For millions of patients worldwide, MRI examinations provide essential and potentially life-saving information. Some devices, such as pacemakers and neurostimulators, have limitations related to MRI safety and may be contraindicated for use with MRI. Even more devices, such as stents, vena cava filters, and some types of catheters and guidewires, are safe for use with MRI but have limited MRI image compatibility. Some of these devices are simply not well-imaged under MRI. Others have properties that interfere with the MRI image by causing an image artifact (distortion) in the area in and around the device, limiting the effectiveness of MRI for assisting placement or diagnostic follow up on these implants. It may be contraindicated in certain situations because the magnetic field present in the MRI environment may, under certain circumstances, result in movement or heating of a metallic orthopaedic implant device. Metals that exhibit magnetic attraction in the MRI setting may be subject to movement (deflection) during the procedure. Both magnetic and non-magnetic metallic devices of certain geometries may also be subjected to heating caused by interactions with the magnetic field. Of secondary concern, is the possibility of image artifacts that can compromise the procedure and image quality.

There are currently several researchers as well as an ASTM committee exploring methods for accurately assessing the MRI compatibility of implant devices. The primary focus of the research has been the measurement of implant movement in response to a magnetic field. Shellock and co-workers [91–93] conducted several studies in which the movement/deflection of various orthopaedic implants was measured in the high magnetic field (0.3–1.5 Tesla) region of MRI units. The results of these studies show no measurable movement of implants fabricated from cobalt, titanium and stainless steel alloys. The movemen/deflection of selected orthopaedic implants in a 3.0 Tesla MRI unit was also examined and it was found that devices fabricated from cobalt, titanium and stainless steel exhibited little or no movement/deflection [94].

Ferromagnetic metal will cause a magnetic field inhomogeneity, which, in turn, causes a local signal void, often accompanied by an area of high signal intensity, as well as a distortion of the image. They create their own magnetic field and dramatically alter precession frequencies of protons in the adjacent tissues. Tissues adjacent to ferromagnetic components become influenced by the induced magnetic field of the metal hardware rather than the parent field and, therefore, either fail to precess or do so at a different frequency and hence do not generate useful signal. Two components contribute to susceptibility artifact, induced magnetism in the ferromagnetic component itself and induced magnetism in protons adjacent to the component. Artifacts from metal may have varied appearances on MRI scans due to different type of metal or configuration of the piece of metal. In relation to imaging titanium alloys are less ferro-magnetic than both cobalt and stainless steel, induce less susceptibility artifact and result in less marked image degradation [94–96].

## 3. Surface Texturing

Surface modifications have been applied to metallic biomaterials in order to improve mechanical, chemical, and physical properties such as wear resistance, corrosion resistance, biocompatibility and surface energy, *etc*. For enhancing the mechanical retention between two surfaces, one or both surfaces are normally modified to increase effective surface area either by sand-blasting, shot-peening, or laser-peening method. Another distinct purpose of surface modification is found on implant surfaces for both dental and orthopedic applications to exhibit biological, mechanical and morphological compatibilities to receiving vital hard/soft tissue, resulting in promoting osseointegration [97]. Such modifications are, in general, divided into two categories: surface concave texturing and surface convex texturing. Surface concave textures can be achieved by either material removal from its surface layer by chemical or electrochemical action, or mechanical indentations (caused by sand-blasting, shot-peening, or laser-peening) [97]. On the other hand, surface convex textured surfaces can be formed by depositing certain types of particles by one of several physical or chemical depositing techniques (like CVD, PVD, plasma-spraying, *etc*.) or diffusion bonding [97]. If density and porosity of deposited particles can be appropriately controlled, a porous surface can be achieved, leading to successful bone ingrowth. Surface roughness measurement is one of the most frequently and easily employed methods to characterize the modified surfaces. Hence, alternation of surface roughness should also be discussed in association with surface modifications.

Biological survival, particularly longevity of biological adhesive joints, is often dependent on thin surface films. Surfaces and interfaces behave completely different from bulk properties, as previously discussed. The characteristics of a biomaterial surface govern the processes involved in biological response. Surface properties such as surface chemistry, surface energy, and surface morphology may be studied in order to understand the surface region of biomaterials [97]. The surface plays a crucial role in biological interactions for four reasons: (1) the surface of a biomaterials is the only part contacting with the bio-environment, (2) the surface region of a biomaterial is almost always different in morphology and composition from the bulk, (3) for biomaterials that do not release or leak biologically active or toxic substance, the characteristics of the surface governs the biological response (foreign material *vs*. host tissue), and (4) some surface properties such as topography affect the mechanical stability of the implant-tissue interface [98–102]. Like the interface, the surface has a certain characteristic thickness, (1) for the case when the interatomic reaction is dominant, such as wetting or adhesion, atoms within a depth of 100 nm (1,000 Å) will be important, (2) for the case of the mechanical interaction, such as tribology and surface hardening, since the elasticity due to the surface contact and the plastically deformed layer will be a governing area, the thickness of about 0.1–10 μm will be important, and (3) for the case when mass transfer or corrosion is involved, the effective layer for preventing the diffusion will be within 1–100 μm [98–102].

As described previously, controlled surface roughness (rugophilicity) plays an important role to enhance the osseointegration of titanium implants [103–105]. Compared to smooth surfaces, osteoblasts can be grown on rough surfaces, which were fabricated in various methods, as will be discussed in details in the following sections [106,107]. One of the most important manufacturing parameters of titanium implants is roughening of the surface for increasing the effective surface area of implant body adjacent to the bone interface, thereby improving the cell attachment, bone apposition and biomechanical stability of the implant [59,108–113].

Such important surfaces can be further modified or altered in a favorable fashion to accommodate, facilitate, or promote more biofunctionality and bioactivity in mechanical, chemical, electrochemical, thermal, or any combination of these methods.

### 3.1. Sand-Blasting

Sand-blasting, as well as shot-peening (which will be discussed in the following section), has three purposes: (1) cleaning surface contaminants prior to further operation, (2) roughening surfaces to increase effective surface area (for example, under some circumstances, the effective surface area could be double than the original surface area), and (3) producing beneficial surface compressive residual stress [114]. As a result, such treated surfaces exhibit higher surface energy, indicating higher surface chemical and physical activities, and enhancing fatigue strength as well as fatigue life due to compressive residual stress [114].

In order to obtain satisfactory fixation and biofunctionality of biotolerated and bioinert materials, some of the mechanical surface alternation such as threaded surface, grooved surface, pored surface, and rough surface have been produced that promote tissue and bone ingrowth [115]. But so far, there is no report on suitable roughness to specific metallic biomaterials. In general, on the macroscopic level (>10 μm), roughness will influence the mechanical properties of the interface, the way stresses are distributed and transmitted, the mechanical interlocking of the interface, and the biocompatibility of biomaterials. On a smaller scale, surface roughness in the range from 10 nm to 10 μm may influence the interface biology, since it is of the same order in size as cells and large biomolecules [48]. Topographic variations of the order of 10 nm and less may become important because microroughness on this scale length consists of material defects such as grain boundaries, dislocation steps, and vacancies, which are known to be active sites for adsorption, and thus may influence the bonding of biomolecules to the implant surface. There is evidence that surface roughness on a micron scale allows cellular adhesion that alters the overall tissue response to biomaterials [48]. Microrough surfaces allow early better adhesion of mineral ions or atoms, biomolecules, and cells, form stronger fixation of bone or connective tissue, result in a thinner tissue-reaction layer with inflammatory cells decreased or absent, and prevent microorganism adhesion and plaque accumulation, when compared with the smooth surfaces [48].

Piattelli *et al*. [116] conducted a histological and histochemical evaluation in rabbits to study the presence of multinucleated giant cells (MGCs) at the interface with machined, sand-blasted (with 150 μm alumina media), and plasma-sprayed titanium implants. It was reported that (i) MGCs were not observed at any of the experimental times around machined and sand-blasted titanium surfaces; whereas (ii) MGCs were present at the interface with titanium plasma-sprayed implants at two weeks and two months, (iii) at four and eight weeks these cells tended to decrease in number, and (iv) an inflammatory infiltrate was not present in connection with the MGCs [116].

Although alumina (Al_2_O_3_) or silica (SiO_2_) particles are most frequently used as a blasting media, there are several different types of powder particles utilized as media [113]. Surface roughness modulates the osseointegration of orthopedic and dental titanium implants [113]. This process may cause the release of cytotoxic silicium or aluminium ions in the peri-implant tissue [113]. To generate a biocompatible roughened titanium surface, an innovative grid-blasting process using biphasic calcium phosphate (BCP) particles was developed by Citeau *et al*. [113]. Ti-6Al-4V discs were either polished, BCP grid-blasted, or left as-machined. BCP grid-blasting created an average surface roughness of 1.57 μm compared to the original machined surface of 0.58 μm. X-ray photoelectron spectroscopy indicated traces of calcium and phosphorus and relatively less aluminum on the BCP grid-blasted surface than on the initial titanium specimen. It was reported that (i) scanning electronic microscopy observations and measurement of mitochondrial activity (MTS assay) showed that osteoblastic MC3T3-E1 cells were viable in contact with the BCP grid-blasted titanium surface, (ii) MC3T3-E1 cells expressed alkaline phosphate (ALP) activity and conserved their responsiveness to bone morphogenetic protein BMP-2, and (iii) the calcium phosphate grid-blasting technique increased the roughness of titanium implants and provided a non-cytotoxic surface with regard to mouse osteoblasts [113]. Tribo-chemical treatment has been proposed to enhance the bond strength between titanium crown and resin base [117]. Using silica-coated alumina as a blasting media under relatively low pressure, silica layer is expected to remain on the blasted surface so that retention force is enhanced by silan-coupling treatment.

Although the recent development of investment materials and casting machines has enabled clinical applications of titanium in dentistry, there remain several problems to be solved. First of all, efficient finishing techniques are required. Titanium is known to be difficult to grind because of its plasticity, stickiness, low heat conductivity, and chemical reactivity at high temperatures [118,119]. Although blasting shows several advantages, there is evidence of adverse effects: (1) surface contamination, depending on type of blasting media, and (2) distortion of blasted workpiece, depending on blasting manner and intensity. Miyakawa *et al*. [120] studied the surface contamination of abraded titanium. Despite low grinding speeds and water cooling, the abraded surfaces were found to be contaminated by abrasive constituent elements. Element analysis and chemical bond state analysis of the contaminants were performed using an electron probe microanalyzer. X-ray diffraction of the abraded surface was performed to identify the contaminants. It was reported that (i) the contamination of titanium is related to its reactivity as well as its hardness, (ii) in spite of water cooling and slow-speed abrading, titanium surfaces were obviously contaminated, (iii) contaminant deposits with dimensions ranging from about 10 to 30 μm occurred throughout the surfaces, and (iv) the contaminant of titanium, although related to the hardness, resulted primarily from a reaction with abrasive materials, and such contamination could negatively influence titanium’s resistance to corrosion and its biocompatibility [120].

Normally, fine alumina particles (50 μm Al_2_O_3_) are recycled within the sand-blasting machine. Ceramics such as alumina are brittle in nature, therefore some portions of recycled alumina might be brittle-fractured. If fractured sand blasting particles are involved in the recycling media, it might result in irregular surfaces, as well as potential contamination. Using fractal dimension analysis [121–123], a sample plate surface was weekly analyzed in terms of topographic changes, as well as chemical analysis of sampled recycled Al_2_O_3_ particles. It was found that after accumulated use time exceeded 30 mins, the fractal dimension (D_F_) remained a constant value of about 1.4, prior to that it continuously increased from 1.25 to 1.4. By the electron probe microanalysis on collected blasting particles, unused Al_2_O_3_ contains 100% Al, whereas used (accumulated usage time was about 2,400 sec) particles contained Al (83.32 wt%), Ti (5.48), Ca (1.68), Ni (1.36), Mo (1.31), S (1.02), Si (0.65), P (0.55), Mn (0.49), K (0.29), Cl (0.26), and V (0.08), strongly indicating that used alumina powder was heavily contaminated, and a high risk for the next material surface to be contaminated. Such contaminants are from previously blasted materials having various chemical compositions, and investing materials as well [124].

There is evidence of surface contamination due to mechanical abrasive actions [125]. As a metallographic preparation, the surface needs to be mechanically polished with a metallographic paper (which is normally SiC-adhered paper) under running water [125]. It is worth mentioning here that polishing paper should be changed between different types of materials, and particularly when a dissimilar metal-couple is used for galvanic corrosion tests, such couple should not be polished prior to corrosion testing because both materials could become cross-contaminated. Hence, there are attempts to use TiO_2_ powder for blasting onto titanium material surfaces. It was reported that titanium surfaces were sand-blasted using TiO_2_ powder (particle size ranging from 45 μm, 45 μm-63 μm, and 63 μm-90 μm) to produce the different surface textures prior to fibroblast cell attachment [126].

In the rabbit tibia, CpTi implants, which were sand-blasted with 25 μm Al_2_O_3_ and TiO_2_ particles, were inserted in the rabbit tibia for 12 weeks [127]. Even though the amount of Al on the implant surface was higher than for the Al_2_O_3_-blasted implants compared to implants not blasted with Al_2_O_3_, any negative effects of the Al element were not detected [128], which is in contrast to those reported by Johansson *et al*. [127], who reported that Al release from Ti-6Al-4V implants was found to coincide with a poorer bone-to-implant over a three month period. It is possible that the lack of differences between TiO_2_-blasted and the Al_2_O_3_-blasted implants depends on lower surface concentrations of toxic Al ions than those reported by Johansson *et al*. [127]. Wang [129] investigated the effects of various surface modifications on porcelain bond strengths. Such modifications included Al_2_O_3_ blasting, TiO_2_ blasting, HNO_3_ + HF + H_2_O treatment, H_2_O_2_ treatment, and pre-oxidation in air at 600 °C for 10 min. Ti-porcelain couples were subjected to 3-point bending tests. It was concluded that TiO_2_ air abrasion showed the highest bond strength, which was significantly different from other surface treatments.

Recently, it was reported that sand-blasting using alumina particle caused a remarkable distortion on a Co-Cr alloy and a noble alloy [130,131]. It was estimated that the stress causing the deflection exceeded the yield strength of tested materials. It was also suggested that the sand-blasting should be done using the lowest air pressure, duration of blasting period, and particle size alumina in order to minimize distortion of crowns and frameworks. To measure distortion, Co-Cr alloy plates (25 mm long, 5 mm wide, 0.7 mm thick) were sand-blasted with Al_2_O_3_ of 125 μm. Distortion was determined as the deflection of the plates as a distance of 20mm from the surface. It was reported that (i) the mean deflections varied between 0.37 mm and 1.72 mm, and (ii) deflection increased by an increase in duration of the blasting, pressure, particle size, and by a decrease in plate thickness [130].

### 3.2. Shot-Peening and Laser-Peening

Shot peening (which is a similar technique to sand-blasting, but has more controlled peening power, intensity, and direction) is a cold working process in which the surface of a part is bombarded with small spherical media called shot. Each piece of shot striking the material acts as a tiny hammer, imparting to the surface small indentations or dimples. In order for the dimple to be created, the surface fibers of the material must be yielded in tension. Below the surface, the fibers try to restore the surface to its original shape, thereby producing below the dimple a hemisphere of cold-worked material highly stressed in compression. Overlapping dimples (which are sometimes called forged dimples) develop an even layer of metal in residual compressive stress. Both compressive stresses and cold working effects are used in the application of shot peening in forming metal parts, called “shot forming” [132].

The laser peening technology is recently developed, claiming non-contact, no-media, and contamination-free peening method [132]. Before treatment, the workpiece is covered with a protective ablative layer (paint or tape) and a thin layer of water. High-intensity (5–15 GW/cm^2^) nanosecond pulses (10–30 ns) of laser light beam (3–5 mm width) striking the ablative layer generate a short-lived plasma which causes a shock wave to travel into the workpiece. The shock wave induces compressive residual stress that penetrates beneath the surface and strengthens the workpiece [133–136], resulting in improvements in fatigue life and retarding in stress corrosion cracking occurrence. Cho *et al*. laser-treated CpTi screws and inserted in right tibia metaphysics of white rabbits for 8 weeks [137]. It was reported that (i) SEM of laser-treated implants demonstrated a deep and regular honeycomb pattern with small pores, and (ii) eight weeks implantation, the removal torque was 23.58 N-cm for control machined and 62.57 N-cm for laser-treated implants. Gaggl *et al*. reported that (i) surfaces of laser-treated Ti implants showed a high purity with appropriate roughness for good osseointegration, and (ii) the laser-treated Ti had regular patterns of micropore with interval of 10–12 μm, diameter of 25 μm, and depth of 20 μm [138].

At the end of this section, it is necessary to summarize various techniques to measure and characterize the surface roughness. They include that (1) surface roughness can be measured using a profilometer with sharp edge stylus, which is a contact method, (2) atomic force microscopy can provide non-contact surface topography from which the surface roughness can be indirectly measured, and (3) fractal dimension analysis can be used to present the surface roughness in non-Euclidian dimension [121,124]. Recently, Hansson *et al*. [139] employed computer simulations to measure surface roughness. The lateral resolution was defined as the pixel size of a profiling system. A surface roughness was simulated by a trigonometric function with random periodicity and amplitude. The function was divided into an array of pixels simulating the pixels of the profiling system. The mean height value for each pixel was used to calculate the surface roughness parameters. It was found that the accuracy of all the surface roughness parameters investigated decreased with increasing pixel size. This tendency was most pronounced for mean slope and developed length ratio, amounting to about 80% of their true values for a pixel size of 20% of the true mean high-spot spacing. It was concluded that the lateral resolution of an instrument/method severely compromises the precision of surface roughness parameters which are measured for roughness features with a mean high-spot spacing less than five times the lateral resolution [139].

### 3.3. Chemical, Electrochemical, and Thermal Modifications

There are several experimental results on chemical, electrochemical, thermal, and combinations of these with regard to altering the Ti surface to facilitate better surface chemical, mechanical, and biological reactions. Endo [140] treated NiTi in 30% NHO_3_, then heated at 400 °C for 0.75 h, and NHO_3_ treatment, followed by boiling in water for 6–14 h. The variously treated NiTi surfaces were tested for dissolution resistance in bovine serum. It was found that (i) those stems thermally treated were found to have significantly lower metal ion release due to stable rutile oxide (TiO_2_) formation, (ii) human plasma fibronection (an adhesive protein) was covalently immobilized onto an alkylaminosilane derivate of NiTi substrate with glutaraldehyde, and (iii) the XPS spectra suggested that gamma-aminopropyltriethoxysilane (γ-APS) was bonded to the surface through metallosiloxane bonds (Ti-O-Si) formed via a condensation reaction between the silanol end of γ-APS and the surface of the hydroxyl group, with a highly cross-linked siloxane network formed after heat treatment of the silanized surface at 100 °C. Based on these findings, it was concluded that human plasma fibronectin was immobilized at the surface, and significantly promoted fibroblast spreading, suggesting that this chemical modification offers an effective means of controlling metal/cell interactions [140]. In study done by Browne *et al*. [141], hip replacement stems manufactured from the Ti-6Al-4V alloy were surface-treated and tested for dissolution resistance in bovine serum. Specimens were degreased in 1,2-dichloroethane vapor and surface treated in one of four ways: (1) 35% nitric acid for 10 min-typical commercial treatment, (2) 35% nitric acid for 16 h and rinsed in distilled water, (3) thermal heating in a furnace for 0.75 h at 400 °C, and (4) 35% nitric acid, then aged in boiling distilled water in a silica beaker for various times, 6, 8, 10, and 14 h. It was found that thermal treatment and aging of surface oxides promote the formation of dense rutile structure. This is effective in reducing metal ion dissolution (up to 80%), particularly in the early stages of implantation where the stem surface is equilibrating with its surroundings. This benefit is further enhanced on rough surfaces with an increased surface area. It was, therefore, concluded that (i) the thermal treatment and aging of the surface oxides are important with respect to cementless and porous implants, and (ii) such treatments could be incorporated in commercial manufacturing procedures to reduce the risk of metal dissolution being a contributory factor towards revision surgery [141].

Krozer *et al*. [142] investigated the possible influence of an amino-alcohol solution on machined Ti surface properties. Screw-shaped CpTi implants and CpTi studs were used. They were rinsed (1) in running deionized water for 2 min, (2) NaCl solution for 2 min followed by deionized water washing, and (3) rinsed in 5% H_2_O_2_ for 2 min followed by deionizd water washing, and rinsed in deionized water for 2 min. The amino-alcohol solution was supplied to the sample surfaces, and four methods were used in order to remove the adsorbed alcohol molecules. It was shown that (i) rinsing in water, saline solution, and 5% H_2_O_2_ did not remove the amino-alcohol from the surface; however (ii) exposure to ozone produced by using a commercial mercury lamp in ambient air resulted in complete removal of the adsorbed amino-alcohol, and (iii) the presence of such a film most likely prevents re-integration to occur at the implant-tissue interface *in vivo* [141]. In study done by Rupp *et al*. [142], CpTi was first blasted with 354–500 μm large grits, followed by (1) HCl/HF/HNO_3_ etching, (2) HCl/H_2_SO_4_ etching, (3) HCl/H_2_SO_4_/HF/ oxalic acid + neutralized, and (4) HCl/H_2_SO_4_/HF/oxalic acid + oxidized. It was reported that the Ti modifications which shift very suddenly from a hydrophobic (high surface contact angle) to a hydrophilic (low surface contact angle) state adsorbed the highest amount of immunologically assayed fibronectin [143]. This is suggesting that microtexturing greatly influenced both the dynamic wettability of Ti implant surfaces during the initial host contact and the initial biological response of plasma protein adsorption.

MacDonald *et al*. [144] investigated the microstructure, chemical composition, and wettability of thermally, and chemically modified Ti-6Al-4V disks, and correlated the results with the degree of adsorption between the radiolabeled fibronectin and Ti-6Al-4V alloy surface and subsequent adhesion of osteoblast-like cells. It was found that (i) heating either in pure oxygen or atmosphere resulted in an enrichment of Al and V within the surface oxide, (ii) heating (in pure oxygen or atmosphere) and hydrogen peroxide treatment, both followed by butanol treatment, resulted in a reduction in content of V, but not in Al, (iii) heating (oxygen/atm) or hydrogen peroxide treatment resulted in a thicker oxide layer and a more hydrophilic surface when compared with chemically-passivated controls (in 40% NHO_3_); however, the post-treatment with butanol resulted in a less hydrophilic surface than heating or hydrogen peroxide treatment alone, and (iv) the greatest increases in the adsorption of radiolabeled fibronectin following treatment were observed with hydrogen peroxide/butanol-treated samples followed by hydrogen peroxide/butanol and heat/butanol, although binding was only increased by 20–40% compared to untreated control. These experiments with radiolabeled fibronectin indicated that enhanced adsorption to the glycoprotein was more highly correlated with changes in chemical composition, reflected in V content and decrease in the V/Al ratio, than with changes in wettability. It was, therefore, concluded that an increase in the absolute content of Al and/or V, or in the Al/V ratio is correlated with an increase in the fibronectin-promoted adhesion of an osteoblast-like cell line [144]. Li *et al*. [145] modified the surface of CpTi (grade 2) implants by the micro-arc oxidation, operated under voltage ranging from 190, 230, 270, 350, 450 and 600 V to form a porous layer. It was found that (i) with increasing voltage, the roughness (from 0.3 to 2.5 μm) and thickness (from 1 μm to 15 μm) of the film increased, and (ii) the TiO_2_ phase changed from anatase to rutile. The micro-arc oxidation was carried out in an aqueous electrolyte with calcium acetate monohydrate and calcium glycerophosphate in deionized water. During the micro-arc oxidation, it was found that (i) Ca and P ions were incorporated into the oxide layer, (ii) the *in vitro* cell responses were also dependent on the oxidation condition, and (iii) with increasing voltage, the alkaline phosphatase activity increased, while the cell proliferation rate decreased. Preliminary *in vivo* tests of the micro-arc oxidation-treated specimens on rabbits showed a considerable improvement in their osseointegration capacity as compared to the un-modified CpTi implant [145].

The surface bioactivity of titanium was investigated after water and hydrogen plasma immersion ion implantation (PI^3^) by Xie *et al*. [146]. PI^3^ method excels in the surface treatment of components possessing a complicated shape such as medical implants. In addition, water and hydrogen plasma immersion ion implantation has been extensively studied as a method to fabricate silicon-on-insulator substrates in the semiconductor industry, and so it is relatively straightforward to transfer the technology to the biomedical field. Water and hydrogen were plasma-implanted into titanium sequentially. It was found that (i) after incubation in simulated body fluids for cytocompatibililty evaluation *in vitro*, bone-like hydroxyapatite was found to precipitate on the (H_2_O + H_2_) implanted samples, while no apatite was found on titanium samples plasma implanted with water or hydrogen alone, and (ii) human osteoblast cells were cultured on the (H_2_O+H_2_)-implanted titanium surface and they exhibited good adhesion and growth. It was, accordingly, suggested that plasma immersion ion implantation is a practical means to improve the surface bioactivity and cytocompatibility of medical implants made of titanium [146].

Rohanizadeh *et al*. [147] investigated methods of preparing different types of titanium oxide (TiO_2_) and their effects on apatite deposition and adhesion on titanium surfaces. CpTi discs were subjected to the following treatments: (1) heat treatment at 750 °C; (2) oxidation in H_2_O_2_ solution followed by heat treatment; (3) dipping in rutile/gelatin slurry; and (4) dipping in anatase/gelatin slurry. Surface-treated Ti discs were immersed in a supersaturated calcium phosphate solution to allow apatite deposition. It was shown that (i) the percentage of area covered by deposited apatite was highest in sample discs which were dipped in an anatase/gelatin slurry, compared to the other groups, (ii) apatite deposited on Ti discs pretreated in H_2_O_2_ solution demonstrated the highest adhesion to the titanium substrate, and (iii) the surface treatment method affects the type of TiO_2_ layer formed (anatase or rutile) and affects apatite deposition and adhesion on the Ti surface [147].

### 3.4. Coating

The coating layer is not only required to exhibit an expected function, depending on its original specific aims, but it is also important to notice that the coating layer is only functional if it adheres well to the metal substrate and if it is strong enough to transfer all loads. Coated substrate possesses at least two layers and one intermediate interface. If such coupled is subjected to stressing, although the strain field should be assumed to be a continuum, the stress field of the couple exhibits a discrete one due to differences in modulus of elasticity, as discussed in the previous section for mechanical compatibility. This discrete stress field results in interfacial stress, and if the interfacial stress is higher than the bonding strength, the couple can be debonded or delaminated, causing the structural integrity to no longer be maintained.

#### 3.4.1. Carbon, Glass, Ceramic Coating

The surface of Ti-6Al-4V has been modified by ion beam mixing a thin carbon film [148]. XPS analysis showed that after mixing, the surface film consists essentially of a Ti compound containing (Ti, O, and C), TiO_2_, Ti, and C. The composition of the surface modified film determined by Rutherford backscattering spectrometry is approximately Ti_0.5_O_0.3_C_0.2_ and its thickness is about 200 μm. It was also reported that after three months immersion in a simulated body fluid, the growth of calcium phosphate species containing both HPO_4_^−^ and H_2_PO_4_^−^ (probably CaHP_4_ and Ca(PO_4_)_2_) have been observed [148]. The corrosion resistance and other surface and biological properties of NiTi were enhanced using carbon plasma immersion ion implantation and deposition (PI^3^). Poon *et al*. [149] mentioned that either an ion-mixed amorphous carbon coating fabricated by plasma immersion ion implantation and deposition or direct carbon PI^3^ can drastically improve the corrosion resistance and block the out-diffusion on Ni from the metal. The tribo-logical tests showed that the treated surfaces are mechanically more superior and cytotoxicity tests revealed that both sets of plasma-treated samples favored adhesion and proliferation of osteoblasts [149]. With regard to potential toxicity of Ni, this is one of methods to prevent or shield the Ni element to diffuse out from NiTi surface. There is another way to achieve the similar outcome by selectively leaching out Ni from the NiTi surface layer by chemically etching the NiTi surface in mixed acid aqueous solution of HF + HNO_3_ + H_2_O (1:1:3 by volume) [66].

Bioactive glass (BAG) is a bioactive material with a high potential as implant material. Reactive plasma spraying produces a feasible BAG-coating for Ti-6Al-4V dental implants. It was shown that (i) the coating withstands, without any damage, an externally generated tensile stress of 47 MPa, and (ii) adhesion testing after two months of *in vitro* reaction in a simulated body fluid showed that coating adhesion strength decreased by 10%, but the implant was still adequate for load-bearing application [150].

Saiz *et al*. [151] evaluated the *in vitro* response in simulated body fluid of silicate glass coating on Ti-6Al-4V. Glasses belonging to the SiO_2_-CaO-MgO-Na_2_O-K_2_O-P_2_O_5_ system were used to prepare 50–70 μm thick coatings by employing a simple enameling technique. It has been found that (i) coatings with silica content lower than 60 wt% are more susceptible to corrosion and precipitate carbonated HA on their surface during *in vitro* tests; however (ii) these coatings have a higher thermal expansion than the metal, (iii), after 2 month in simulated body fluid, crack grows in the coating, reaches the glass/metal interface and initiates delamination, and (iv) glasses with silica content higher than 60wt% are more resistant to corrosion and have lower thermal expansion, and these coatings do not crack, but such glasses with silica do not precipitate apatite even after two months in simulated body fluid [151]. Lee *et al*. [152] prepared calcium-phosphate, apatite-wollastonite (CaSiO_3_) (1:3 by volume fraction) glass ceramic, apatite-wollastonite (1:1) glass ceramic, and bioactive CaO-SiO_2_-B_2_O_3_ glass ceramic coatings by the dipping method. Coated and uncoated Ti-6Al-4V screws were inserted into the tibia of 18 adult mongrel male dogs for 2, 4 and 8 weeks. It was found that (i) at 2, 4, and 8 weeks, the extraction torque of these ceramic-coated screws was significantly higher than the corresponding insertion torque, and (ii) strong fixation was observed even at two weeks in all three coatings except CaO-SiO_2_-B_2_O_3_ glass ceramic coating [152].

#### 3.4.2. Hydroxyapatite Coating

Enhancement of the osteoconductivity of Ti implants is potentially beneficial to patients since it shortens the medical treatment time and increases the initial stability of the implant. To achieve better osteoconductivity, apatite [Ca_10-x_(HPO_4_)_x_(PO_4_)_6-x_(OH)_2-x_] coating has been commonly employed on the Ti implant surface [152].

Hydroxyapatite is a major mineral component in animal and human bodies [153]. It has been used widely not only as a biomedical implant material but also as biological chromatography supports in protein purification and DNA isolation. Spherical HA ceramic beads have recently been developed that show improvements in mechanical properties and physical and chemical stability. These spherical ceramic beads are typically 20–80 μm in size. There are advantages to reducing the granule size of the spherical HA material: (1) the smaller the granule size, the higher the specific surface area and the higher the bonding capacity, (2) theoretically, the specific surface area (*i.e.*, surface area per volume) is proportional to 6/d, where d is the diameter of the spherical granule, (3) in addition, the mechanical properties of a packed column can be improved by reducing the granule size, resulting in more contacting surface areas, and thereby greater frictional forces between granules, and (4) furthermore, a uniform pack is expected to have a homogeneous pore distribution [154]. The porosity and the specific surface areas of the HA material can be controlled by changing the morphology of the granules, for example, the solid spheres and doughnuts. Hence, Luo *et al*. [155] introduced a new method to produce spherical HA granules ranging in size from 1 to 8 μm with controlled morphology. This method involves an initial precipitation followed by a spray-drying process, which is the controlling step, to produce granules with various structures. It was reported that by adjusting the operating parameters (e.g., atomization pressure) and starting slurry (e.g., concentration), the hollow or solid spheres and doughnut-shaped HA were fabricated.

BSE (bovine spongiform encephalopathy) or ‘mad cow disease’ could have been caused by animal-feed contaminated with human remains a controversial theory. Accordingly, although bovine-derived hydroxyapatite was used as a semi-natural HA [154], the articlers reviewed here are limited to those published before the BSE issue became to be addressed and received a public attention.

With the growing demands of bioactive materials for orthopedic as well as maxillofacial surgery, the utilization of hydroxyapatite (HA, with Ca/P = 1.67) and tri-calcium phosphate (TCP, with Ca/P = 1.50) as fillers, spacers, and bone graft substitutes has received great attention during the past two to three decades, primarily because of their biocompatibility, bioactivity, and osteoconduction characteristics with respect to host tissue. Porous hydroxyapatite granules with controlled porosity, pore size, pore size distribution, and granule size were fabricated using a drip-casting process. Granules with a wide range of porosity from 24 to 76 vol.%, pore size from 95 to 400 μm, and granular sizes from 0.7 to 4 mm can be obtained. This technique allows the fabrication of porous granules with desirable porous characters simulating natural bone architecture, and is expected to provide advantages for biomedical purposes [155].

Plasma-sprayed HA-coated devices demonstrated wide variability in the percentage of the HA coating remaining on the stems. Porter *et al*. [156] reported that (i) the coating was missing from a substantial portion of a stem after only about six months of implantation, and (ii) many ultrastructural features of the bone bonded to the HA coatings on these implants from human subjects were comparable to those on HA-coated devices implanted in a canine model. The geometric design and chemical compositions of an implant surface may have an important part in affecting early implant stabilization and influencing tissue healing.

The influence of different surface characteristics on bone integration of titanium implants was investigated by Buser *et al*. [54]. Hollow-cylinder implants with different surfaces were placed in the metaphyses of the tibia and femur in six miniature pigs. After three and six weeks, the implants with surrounding bone were removed and analyzed in undecalcified transverse sections. The histologic examination revealed direct bone-implant contact for all implants. However, the morphometric analyses demonstrated significant differences in the percentage of bone-implant contact, when measured in cancellous bone. It was reported that (i) electropolished, as well as the sand-blasted and acid pickled (medium grit, HF/HNO_3_) implant surfaces, had the lowest percentage of bone contact with mean values ranging between 20 and 25%, (ii) sand-blasted implants, with a large grit, and titanium plasma-sprayed implants demonstrated 30–40% mean bone contact, and (iii) the highest extent of bone-implant interface was observed in sand-blasted and acid attacked surfaces (large grit; HCl/H_2_SO_4_) with mean values of 50–60%, and hydroxyapatite (HA)-coated implants with 60–70%. It was therefore concluded that the extent of the bone-implant interface is positively correlated with an increasing roughness of the implant surface. Moreover the morphometric results indicated that (i) rough implant surfaces generally demonstrated an increase in bone apposition compared to polished or fine structured surfaces, (ii) the acid treatment with HCl/H_2_SO_4_ used for sand-blasted with large grit implants has an additional stimulating influence on bone apposition, (iii) the HA-coated implants showed the highest extent of bone-implant interface, and (iv) the HA coating consistently revealed signs of resorption. It was suggested that sand-blasting and chemical etching with HCl/H_2_SO_4_ as well as HA coating, seemed to be the most promising alternatives to titanium implants with smooth or titanium plasma-sprayed surfaces [54].

Souto *et al*. [157] investigated the corrosion behavior of four different preparations of plasma-sprayed hydroxyapatite (HA) coatings (50 μm and 200 μm) on Ti-6Al-4V substrates in static Hank’s balanced salt solution through DC potentiodynamic and AC impedance EIS techniques. Because the coatings are porous, ionic paths between the electrolytic medium and the base material can eventually be produced, resulting in the corrosion of the coated metal. It was concluded that significant differences were found in electrochemical behavior for similar nominal thicknesses of HA coatings obtained under different spraying [157]. Filiaggi *et al*. [158] reported that evaluations of an HA-coated Ti-6Al-4V implant system using a modified short bar technique for interfacial fracture toughness determination revealed relatively low fracture toughness values. Using high resolution electron spectroscopic imaging, evidence of chemical bonding was revealed at the plasma-sprayed HA/Ti-6Al-4V interface, although bonding was primarily due to mechanical interlocking at the interface [158]. The modulus of elasticity, residual stress and strain, bonding strength, and microstructure of the plasma-sprayed hydroxyapatite coating were evaluated on Ti-6Al-4V substrate with and without immersion in Hank’s balanced salt solution. It was reported that (i) the residual stress and strain, modulus of elasticity, and bonding strength of the plasma-sprayed HA coating after immersion in Hank’s solution were substantially decreased, and (ii) the decayed modulus of elasticity and mechanical properties of HA coatings were caused for by the degraded interlamellar or cohesive bonding in the coating due to the increased porosity after immersion that weakens the bonding strength of the coating and substrate system. It was also suggested that the controlled residual stress and strain in the coating might promote the long-term stability of the plasma-sprayed HA-coated implant [159]. Yang *et al*. [160] investigated the effect of titanium plasma-sprayed (TPS) and zirconia (ZrO_2_)-coated titanium substrates on the adhesive, compositional, and structural properties of plasma-sprayed HA coatings. Apatite-type and α-tricalcium phosphate phases were observed for all HA coatings. The coating surfaces appeared rough and melted, with surface roughness correlating to the size of the starting powder. It was found that (i) no significant difference in the Ca/P ratio of HA on Ti and TPS-coated Ti substrates was observed, (ii) however, the Ca/P ratio of HA on ZrO_2_-coated Ti substrate was significantly increased, (iii) interfaces between all coatings and substrates were observed to be dense and tightly bound, except for HA coatings on TPS-coated Ti substrate interface; however (iv) an intermediate TPS or ZrO_2_ layer between the HA and Ti substrate resulted in a lower adhesive strength as compared to HA on Ti substrate [160].

HA can be admixed with Ti powder. 80HA-20Ti powder and 90HA-10Ti powder (by weight) were mechanically mixed and dry-pressed and heat treated at 1,100 °C for 30 min in vacuum (less than 6 × 10^−3^ Pa). Heat treatment of HA specimens in vacuum resulted in the loss of hydroxyl groups, as well as the formation of a secondary β-tricalcium phosphate phase. It was concluded that the *in vacuo* heat treatment process completely converted the metal-ceramic composites to ceramic composites [161]. Moreover, using air plasma spraying and vacuum plasma spraying methods, HA and a mixture of HA + Ti were deposited on Ti-6Al-4V. It was reported that a higher adhesion was obtained with vacuum plasma spraying rather than with air plasma spraying [162].

Lee *et al*. [163] employed the electron-beam deposition method to obtain a series of fluoridated apatite coatings. The fluoridation of apatite was aimed to improve the stability of the coating and elicit the fluoride effect, which is useful in the dental restoration area. Apatite fluoridated at different levels was used as initial evaporants for the coatings. It was observed that (i) the as-deposited coatings were amorphous, but after heat-treated at 500 °C for 1 h, the coatings crystallized well to an apatite phase without forming any cracks, (ii) the adhesion strengths of the as-deposited coatings were about 40 MPa, and after heat treatment at 500 °C, it decreased to about 20 MPa; however the partially fluoridated coating maintained its initial strength. It was also reported that (i) the osteoblast-like cells responded to the coatings in a similar manner to the dissolution behavior, (ii) the cells on the fluoridate coatings showed a lower proliferation level compared to those on the pure HA coating, and (iii) the alkaline phosphatase activity of the cells was slightly lower than of on the pure HA coating [163]. Kim *et al*. [164] deposited HA and fluoridated HA films on CpTi (grade 2). HA sol was prepared by mixing P(C_2_H_5_O)_3_ and distilled water, and fluoridated HA sol was prepared by NH_4_F in P containing solution (like HA by replacing the OH group with F ions) and their films were deposited on CpTi (grade II). The mixture was stirred at room temperature for 72 hours. Dipping CpTi into the solutions was performed at 500 °C for 1 h. It was concluded that (i) the coatings layers were dense, uniform, and had a thickness of approximately 5μm after heat treatment at 500 °C, (ii) the fluoridated HA layer showed much lower dissolution rate (in 0.9% NaCl as physiological saline solution) than pure HA, suggesting the tailoring of solubility with F-incorporation within the apatite structure, (iii) the osteoblast-like MG63 and HOS cells grew and proliferated favorably on both coatings and pure Ti, and (iv) especially, both coated Ti exhibited higher alkaline phosphatase expression levels as compared to non-coated Ti, confirming the improved activity and functionality of cells on the substrate via the coatings [164].

There are several studies done on effects of post-spray coating heat-treatment on mechanical and structural integrities of the coated film. A post-plasma-spray heat treatment (at 960 °C for 24 h followed by slow furnace cooling) was performed to enhance chemical bonding at the metal/ceramic interface, and hence improve the mechanical properties. It was found that any improvements realized were lost due to the chemical instability of the coating in a moisture-laden environment, with a concomitant loss in bonding properties, and this deterioration appeared to be related to environmentally assisted crack growth as influenced by processing conditions [165]. HA coatings on Ti-6Al-4V were annealed at 400 °C in air for 90 h, and were evaluated as post-coating heat treatment. It was found that (i) the oxide species TiO_2_, Al_2_O_3_, V_2_O_5_, V_2_O_3_ and VO_2_ were present on both as-coated and as heat-treated samples, and (ii) the fatigue resistance of the substrate was found to be significantly reduced by the heat treatment, due to the stress relief [166]. Ti-6Al-4V substrate was heated at 25°, 160°, and 250 °C, followed by cooling in air or air + CO_2_ gas during operating of the HA plasma coating. It was reported that (i) when residual compressive stress was 17 MPa the bonding strength was 9 MPa, while the bonding strength continuously reduced to 3 MPa for a residual compressive stress of about 23 MPa, and (ii) the compressive residual stress weakened the bonding at the interface of the HA and the Ti-substrate [167], due to remarkable mechanical mismatching.

As has been reviewed in the above, a plasma spray method has been widely accepted as the apatite coating method because it gives tight adhesion between the apatite coating and Ti. However, the plasma spray method employs extremely high temperatures (10,000–12,000 °C) during the coating process. Unfortunately, it results in potentially serious problems including (1) an alteration of structure, (2) formation of apatite with extremely high crystallinity, and (3) long term dissolution and the accompanying debonding of the coating layer. To reduce these drawbacks associated with the conventional plasma spray method, a high viscosity flame spray method was developed. Although the high viscosity flame spray method employs lower temperature compared with the plasma spray method, it is still 3,000 °C, which is adequate to alter the crystal structure and formation of apatite with extremely high crystallinity. To avoid shortcomings in the apatite coating methods using high temperatures, many alternative room temperature coating methods were studied extensively including ion beam sputtering, dipping, electrophoretic deposition, and electrochemical deposition [168]. Mano *et al*. [168] developed a new coating method called blast coating, using common sand-blasting equipment at room temperature. Blast-apatite coated CpTi implants were inserted in tibia of rats for 1, 3, and 6 weeks. It was found that (i) the apatite coating adhered tightly to the Ti surface even after the 6 week implantation, (ii) the implants cause no inflammatory response, showing strong bone response and much better osteoconductivity compared with the uncoated CpTi implant, (iii) the new bone formed on the surface of coated implants was thinner compared with that formed on the surface of Ti implant. Therefore, the blast-apatite implant has a good potential as an osteoconductive implant material [168].

Plasma-sprayed HA coatings on commercial orthopedic and dental implants consist of mixtures of calcium phosphate phases, predominantly a crystalline calcium phosphate phase, hydroxyapatite, and an amorphous calcium phosphate with varying ratios. Alternatives to the plasma-spray method are being explored because of some of its disadvantages, as mentioned before. Moritz *et al*. [169] developed a two-step (immersion and hydrolysis) method to deposit an adherent apatite coating on titanium substrate. First, titanium substrates were immersed in acidic solution of calcium phosphate, resulting in the deposition of a monetite (CaHPO_4_) coating. Second, the monetite crystals were transformed to apatite by hydrolysis in NaOH solution. Energy dispersive spectroscopy had revealed the presence of calcium and phosphorous elements on the titanium substrate after removing the coating using tensile or scratching tests. It was reported that (i) the average tensile bond of the coating was 5.2 MPa and cohesion failures were observed more frequently than adhesion failures, (ii) this method has the advantage of producing a coating with homogenous composition on even implants of complex geometry or porosity, and (iii) this method involves low temperatures and, therefore, can allow the incorporation of growth factors or biogenic molecules [169].

A novel method to rapidly deposit bone apatite-like coatings on titanium implants in simulated body fluid (SBF) has been proposed by Han *et al*. [170]. The processing was composed of two steps: micro-arc oxidation of titanium to form titania (TiO_2_) films, and UV-light illumination of the titania-coated titanium in SBF. It was reported that (i) the micro-arc oxidation films were porous and nanocrystalline, with pore sizes varying from 1 to 3 μm and grain sizes varying from 10–20 to 70–80 nm; the predominant phase in titania films changed from anatase to rutile, and the bond strength of the films decreased from 43.4 to 32.9 MPa as the applied voltage increased from 250 to 400 V, (ii) after UV-light illumination of the films in SBF for 2 h, bone apatite-like coating was deposited on the micro-arc oxidation film formed at 250 V, and (iii) the bond strength of the apatite/titania bilayer was about 44.2 MPa [170].

The technique was further developed using the sol-gel method [164] to coat Ti surface with hydroxyapatite (HA) films [171]. The coating properties, such as crystallinity and surface roughness, were controlled and their effects on the osteoblast-like cell responses were investigated. The obtained sol-gel films had a dense and homogeneous structure with a thickness of about 1μm. It was found that (i) the film heat-treated at higher temperature had enhanced crystallinity (600 °C > 500 °C > 400 °C), while retaining similar surface roughness, (ii) when heat-treated rapidly (50 °C/min), the film became quite rough, with roughness parameters being much higher (4–6 times) than that obtained at a low heating rate (1 °C/min), and (iii) the dissolution rate of the film decreased with increasing crystallinity (400 °C > 500 °C > 600 °C), and the rougher film had slightly higher dissolution rate. The attachment, proliferation, and differentiation behaviors of human osteosarcoma HOS TE85 cells were affected by the properties of these films. It was further reported that (i) on the films with higher crystallinity (heat treated over 500 °C), the cells attached and proliferated well, and expressed alkaline phosphatase and osteocalcin to a higher degree as compared to the poorly crystallized film (heat treated at 400 °C), and (ii) on the rough film, the cell attachment was enhanced, but the alkaline phosphatase and osteocalcin expression levels were similar as compared to the smooth films [171].

The sol-gel method was favored due to the chemical homogeneity and fine grain size of the resultant coating, and the low crystallization temperature and mass-producibility of the process itself. The sol-gel-derived HA and TiO_2_ films, with thicknesses of about 800 and 200 nm, adhered tightly to each other and to the CpTi (grade 2) substrate. It was reported that (i) the highest bond strength of the double layer coating was 55 MPa after heat treatment at 500 °C due to enhanced chemical affinity of TiO_2_ toward the HA layer, as well as toward the Ti substrate, (ii) human osteoblast-like cells, cultured on the HA/TiO_2_ coating surface, proliferated in a similar manner to those on the TiO_2_ single coating and on the CpTi surfaces; however (iii) alkaline phosphatase activiy of the cells on the HA/TiO_2_ double was expressed to a higher degree than on the TiO_2_ single coating and CpTi surfaces, and (iv) the corrosion resistance of Ti was improved by the presence of the TiO_2_ coating, as confirmed by a potensiodynamic polarization test [172]. Sol-gel-derived TiO_2_ coatings are known to promote bone-like hydroxyapatite formation on their surfaces *in vitro* and *in vivo*. Hydroxyapatite integrates into bone tissue. In some clinical applications, the surface of an implant is simultaneously interfaced with soft and hard tissues, so it should match the properties of both. Ergun *et al*. [173] studied the chemical reactions between hydroxylapatite (HA) and titanium in three different kinds of experiments to increase understanding the bond mechanisms of HA to titanium for implant materials. HA powder was bonded to a titanium rod with hot isostatic pressing. Interdiffusion of the HA elements and titanium was found in concentration profiles measured in the electron microprobe. Titanium was vapor-deposited on sintered HA discs and heated in air; perovskite (CaTiO_3_) was found on the HA surface with Rutherford backscattering and X-ray diffraction measurements. Powder composites of HA, titanium, and TiO_2_ were sintered at 1,100 °C; again, perovskite was a reaction product, as well as β-Ca_3_(PO_4_)_2_, from a decomposition of the HA. Based on these findings, it was reported that (i) chemical reactions and interdiffusion between HA and TiO_2_ during sintering resulted in chemical bonding between HA and titanium; thus, cracks and weakness at HA-titanium interfaces probably result from mismatch between the coefficients of thermal expansion of these materials, and (ii) HA composites with other ceramics and different alloys should lead to better thermal matching and better bonding at the interface [173].

The effects of HA coating and macrotexturing of Ti-6Al-4V was tested by Hayashi *et al*. [174,175]. Implants were inserted in dog’s femoral condyles. It was demonstrated that when grooved Ti implants were used, the addition of HA coating significantly improved the biological fixation. In addition, a grooved depth of 1 mm was found to give significantly better fixation than 2 mm. When compared to implants with traditional diffusion-bonded bead-coated porous surfaces, HA-coated grooved Ti implants were found to show better fixation at 4 weeks after implantation, but significantly inferior fixation at 12 weeks. Hence, it was concluded that while a groove depth of 1 mm was optimal in HA-coated and further grooved Ti implants, they are still inferior to bead-coated Ti implants with respect to longer-term fixation [174]. Two different groups of HA coated and uncoated porous Ti implants, 250–350 μm and 500–700 μm diameter beads, were press-fitted into femoral canine cancellous bone, for 12 weeks. It was reported that (i) the percentage of bone and bone index were higher in the HA coated implants, when comparing HA coated *vs*. uncoated implant in the 250–350 μm bead diameter group, (ii) comparing 500–700 μm, bone ingrowth and bone depth penetration were higher in HA coasted samples, and (iii) the HA-coating was an effective method for improving bone formation and ingrowth in the porous implants [176].

Several HA-coated and uncoated Ti-6Al-4V femoral endoprostheses were evaluated in adult dogs for 52 weeks. It was found that (i) histologic sections from the uncoated grooved implants showed no direct bone-implant apposition with no fibrous tissue interposition after up to 10 weeks, and (ii) the HA-coated grooved implants demonstrated extensive direct bone-coating apposition after five weeks [177]. HA-coated cylindrical plugs of CpTi, Cp-Ti screws, and partially HA-coated CpTi screws were inserted in tibia of adult New Zealand white rabbits for three months. The histological results demonstrated that although there were no marked differences in bony reaction at the cortical level to the different implant materials, HA-coating appeared to induce more bone formation in the medullary cavity. It was also noted that 3 months after insertion loss of coating thickness had occurred [178]. Threaded HA-coated CpTi implants were inserted in the rabbit tibial metaphysis. After six weeks and 1 year post-insertion, the semiloaded implants were histomorphometrically analyzed. It was reported that (i) there was more direct bony contact with the HA-coated implants after 6 weeks, and (ii) one year after insertion, there was significantly more direct bone-to-implant contact with the uncoated CpTi controls, suggesting that the HA coating does not necessarily improve implant integration over a long time period [179]. After six or 12 weeks, four goats were used for mechanical tests and three for histology. To cope with the severe femoral bone stock loss encountered in revision surgery, impacted trabecular bone grafts were used in combination with an HA-coated Ti stem. In this first experimental study, the results indicated that this technique had a high complication rate. However, it was shown that impacted grafts sustained the loaded stems and that incorporation of the graft occurred with a biomechanically stable implant. The technique allows gradual graft incorporation and stability, but more investigations are needed before its introduction into clinical practice [180]. Surface treatments, such as sand-blasting or deposit or rough pure Ti obtained by vacuum plasma spraying, bring about an increase of passive and corrosion current density values as a consequence of the increase of the surface exposed to the aggressive environment. It should, however, be outlined that in the case of rough Ti, only Ti ions are released instead of Al or V out of Ti-6Al-4V. The presence of deposits of HA by vacuum plasma spraying causes an increase of about one order of magnitude in the passive and corrosion current density of the metallic substrate [181].

There are several works in regard to mechanical properties of HA and bond strength of HA layer. Cook *et al*. [182] conducted interface shear strength tests using a transcortical push-out model in dogs after 3, 5, 6, 10 and 32 weeks on CpTi-coated Ti-6Al-4V and HA-coated Ti-6Al-4V. The mean values for interface shear strength increased up to 7.27 MPa for HA-coated implants after 10 weeks of implantation, while uncoated CpTi had 1.54MPa [182]. Yang *et al*. [183] reported that the direction of principal stresses were in proximity to and perpendicular to the spraying direction. The measured modulus of elasticity (MOE) of HA (16 GPa at maximum) was much lower than the theoretical value (*i.e.*, 112 GPa). The denser, well-melted HA exhibited a higher residual stress (compressive, 10–15 MPa at maximum), as compared with the less dense, poor-melting HA. The denser coatings could be affected by higher plasma power and lower powder feed rate. It was also reported that the thicker 200 μm HA exhibited higher residual stress than that of the thinner 50 μm HA [183]. Mimura *et al*. [184] characterized morphologically and chemically the coating-substrate interface of a commercially available dental implant coated with plasma-sprayed HA, when subjected to mechanical environment. A thin Ti oxide film containing Ca and P was found at the interface on Ti-6Al-4V. When the implant was subjected to mechanical stress, a mixed mode of cohesive and interfacial fractures occurred. The cohesive fracture was due to separation of the oxide film from the substrate, while the interfacial fracture was due to the exfoliation of the coating from the oxide film bonded to the substrate. It was reported that (i) microanalytical results showed diffusion of Ca into the metal substrate, hence indicating the presence of chemical bond at the interface; however, (ii) mechanical interlocking seemed to play the major role in the interfacial bond [184]. Lynn *et al*. [166] conducted uniaxial fatigue tests (σ_max_/σ_min_ stress ratio, R = −1, stress amplitude of 620 MPa, frequency of 50Hz) on blasted-Ti-6Al-4V with HA coating on Ti-6Al-4V with film thickness ranging from 0, 25, 50, 75, 100, and 150 μm. It was found that (i) samples with 150 μm were shown to have significantly decreased fatigue resistance, while coatings of 25–100 μm were found to have no effect on fatigue resistance, and (ii) HA coatings with 25–50 μm show no observable delamination during fatigue tests, while coatings with 75–150 μm thick were observed to spall following but not prior to the initiation of the first fatigue crack in the substrate [166].

There are several studies done on apatite-like formation without HA coating. Ti can form a bone-like apatite layer on its surface in simulated body fluid (SBF) when it is treated in NaOH. When pre-treated Ti is exposed to SBF, the alkali ions are released from the surface into the surrounding fluid. The sodium ions increase the degree of supersaturation of the soaking solution with respect to apatite by increasing pH. On the other hand, the released Na^+^ causes an increase in external alkalinity that triggers an inflammatory response and leads to cell death. Therefore, it would be beneficial to decrease the release of Na^+^ into the surrounding tissue. It was found that (i) the rate of apatite formation was not significantly influenced by a lower amount of Na^+^ ion in the surface layer, and (ii) Ti with the lowest content of Na^+^ could be more suitable for implantation in the human body (4 at.%) [185]. Jonášová *et al*. [186] pre-treated CpTi surfaces: (1) in 10 M NaOH at 60 °C for 24 h, and (2) etched in HCl under inert atmosphere of CO_2_ for 2hr, followed by 10 M NaOH treatment. It was found that Ti treated in NaOH can form hydroxycarbonated apatite after exposition in SBF. Generally, Ti is covered with a passive oxide layer. In NaOH this passive film dissolves and an amorphous layer containing alkali ions is formed. When exposed to SBF, the alkali ions are released from the amorphous layer and hydronium ions center into the surface layer, resulting in the formation of Ti-OH groups in the surface. The acid etching of Ti in HCl under inert atmosphere leads to the formation of a micro-roughened surface, which remains after alkali treatment in NaOH. It was shown that the apatite nucleation was uniform and the thickness or precipitated hydroxycarbonated apatite layer increased continuously with time. The treatment of Ti by acid etching in HCl and subsequently in NaOH, is a suitable method for providing the metal implant with bone-bonding ability [186]. Wang *et al*. [187] reported that bone-like apatite was formed on Ti-6Al-4V surfaces pre-treated in NaOH solution immersed in SBF, while no apatite was formed on untreated Ti-6Al-4V. The increase in electrical resistance (by EIS) in the outermost surface of pre-treated Ti-6Al-4V indicated apatite nucleation. With an increase in immersion time in SBF, islands of apatite were seen to grow and coalesce on pre-treated Ti-6Al-4V under SEM. It was also mentioned that the growth of apatite corresponded to the increase in electrical resistance of the surface layer [187].

#### 3.4.3. Ca-P Coating

As we have just reviewed the evidence showed a formation of an apatite-like film containing Ca and P ions when Ti was pre-treated in NaOH, followed by immersion in SBF (simulated body fluid) or PBL (phosphate buffered liquid). Clinically, it was found that for Ti an increase in oxide thickness and an incorporation of Ca and P were found in placed Ti implants which were in the shape of screws, and had been osseointegrated in patients’ jaws for times ranging from 0.5 to 8 years [30]. These provide “hindsight” knowledge, where later on people will use this knowledge to modify the Ti surfaces.

Formation of calcium-phosphate on alkali- (10 M NaOH at 60 °C for 24 h) and heat-treated (alkali-treated then at 600 °C for 1 hfollowed by furnace cooling) CpTi was tested. Samples were immersed in the revised SBF with the same HCO_3_^−^ concentration level as in human blood plasma. It was reported that (i) electron diffraction for the precipitates revealed that octacalcium phosphate, instead of HA, directly nucleates from amorphous calcium-phosphate, (ii) the octacalcium phosphate (OCP) crystals continuously grew on the Ti surfaces rather than transforming to AP, and (iii) calcium titanate (CaTiO_3_) was identified by electron diffraction [188]. Hanawa *et al*. [189] characterized surface films formed on titanium specimens which were immersed in electrolyte solutions (pH: 4.5, 5.1, 7.4) at 37 °C for 1 h, 1 day, 30 days and 60 days by XPS, FTIR-RAS Fourier transform infrared reflection absorption spectroscopy to understand the reaction between Ti and inorganic ions. For comparison, the surfaces of Ti-6Al-4V and NiTi were also characterized. XPS data revealed that calcium phosphates (CP) were naturally formed on these specimens. In particular, compared with the CP formed on the Ti alloys, the CP formed on Ti immersed for 30 days in the solution with pH 7.4 was more like HA. It was also reported that (i) the compositions of the CP formed on the specimens changed with the immersion time and the pH value of the solution (the solution with pH 7.4 was Hank’s balanced solution without organic species, solution with pH 4.5 was the artificial saliva without sodium sulphide and urea), and (ii) a CP similar to AP is naturally formed on Ti in a neutral electrolyte solution in 30 days [189].

Calcium phosphate (Ca-P) coatings have been applied onto titanium alloy prostheses to combine the strength of the metals with the bioactivity of Ca-P. It has been clearly shown in many publications that a Ca-P coating accelerates bone formation around the implant. However, longevity of the Ca-P coating for an optimal bone apposition onto the prosthesis remains controversial. Barrère *et al*. [190] evaluated biomimetic bone-like carbonate apatite (BCA) and octacalcium phosphate (OCP) coatings which were deposited on Ti-6Al-4V samples to evaluate their *in vitro* and *in vivo* dissolution properties. The coated plates were soaked in α-MEM for 1, 2, and 4 weeks, and they were analyzed by Back Scattering Electron Microscopy (BSEM) and by Fourier Transform Infra Red spectroscopy (FTIR). Identical coated plates were implanted subcutaneously in Wistar rats for similar periods. BSEM, FTIR, and histomorphometry were performed on the explants. *In vitro* and *in vivo*, a carbonate apatite (CA) formed onto OCP and BCA coatings via a dissolution-precipitation process. It was reported that (i) *in vitro*, both coatings dissolved overtime, whereas *in vivo* BCA calcified and OCP partially dissolved after 1 week, and (ii) thereafter, OCP remained stable. This different *in vivo* behavior can be attributed to (1) different organic compounds that might prevent or enhance Ca-P dissolution, (2) a greater reactivity of OCP due to its large open structure, or (3) different thermodynamic stability between OCP and BCA phases. It was, therefore, concluded that these structural and compositional differences promote either the progressive loss or calcification of the Ca-P coating, and might lead to different osseointegration of coated implants [190]. Barrère *et al*. [191] studied the nucleation and growth of a calcium phosphate (Ca-P) coating deposited on titanium implants from simulated body fluid (SBF), using atomic force microscopy (AFM) and environmental scanning electron microscopy (ESEM). Forty titanium alloy plates were assigned into two groups: a smooth surface group having a maximum roughness *R*_max_ < 0.10 μm, and a rough surface group with an *R*_max_ < 0.25 μm. Titanium samples were immersed in SBF concentrated by five (SBF × 5) from 10 min to 5 hours and examined by AFM and ESEM. It was observed that scattered Ca-P deposits of approximately 15 nm in diameter appeared after only 10 min of immersion in SBF × 5, and (ii) these Ca-P deposits grew up to 60–100 nm after 4 h on both smooth and rough Ti-6Al-4V substrates. It was found that (i) a continuous Ca-P film formed on titanium substrates, (ii) a direct contact between the Ca-P coating and the Ti-6Al-4V surface was observed, and (iii) the Ca-P coating was composed of nanosized deposits and of an interfacial glassy matrix, which might ensure the adhesion between the Ca-P coating and the Ti-6Al-4V substrate. The Ca-P coating detached from the smooth substrate, whereas the Ca-P film extended onto the whole rough titanium surface over time. In the case of rough Ti-6Al-4V, the Ca-P coating evenly covered the substrate after immersion in SBF × 5 for 5 h. Accordingly, it was suggested that (i) the heterogeneous nucleation of Ca-P on titanium was immediate and did not depend on the Ti-6Al-4V surface topography, and (ii) the further growth and mechanical attachment of the final Ca-P coating strongly depended on the surface, for which a rough topography was beneficial [191].

The bioconductivity of a new biomedical Ti-29Nb-13Ta-6Zr alloy was achieved by a combination of surface oxidation and alkaline treatment [192]. It was reported that (i) immersion in a protein-free SBF and fast calcification solution led to the growth of CP phase on the oxidized and alkali-treated (10 M NaOH at 40 °C for 24 h) alloy, and the new bioconductive surface was still harder than the substrate, (ii) oxidation at 400 °C × 24 h led to the formation of a hard layer, (iii) the oxides are mainly composed of TiO_2_, with small amounts of Nb_2_O_5_ and ZrO_2_; an oxygen diffusion layer exists beneath the surface oxide layer, and (iv) a titanate layer forms on the pre-oxidized surface after alkali treatment, and growth of a layer of Ca-P has been successfully induced on the titanate layer by immersing in SBF [192]. Bogdanski *et al*. [193] coated NiTi with CP by dipping in oversaturated CP solution. Since polymorphonuclear neutrophil grannucytes (PMN) belong to the first cells which will adhere to implant materials, the apoptosis of isolated human PMN after cell culture on non-coated and CP-coated SME NiTi was analyzed by light and scanning electron microscopy and flow cytometry. It was found that (i) in contrast to PMN adherent to non-coated TiNi, the apoptosis of PMN adherent CP-coated samples was inhibited, and (ii) cell culture media obtained from cultured leukocytes with CP-coated were able to transfer the apoptosis inhibiting activities to freshly isolated PMN [193]. The compositions of the surface and the interface of calcium phosphate ceramic (CP) coatings electrophoretically deposited and sintered on CpTi and Ti-6Al-4V were evaluated before and after four weeks immersion in a simulated physiological solution. In the CP coating-metal interface, it was mentioned that (i) the phosphorus diffused beyond the Ti oxide layer, resulting in the depleted phosphorus in the ceramic adjacent to the metal, and (ii) the surface of the ceramic, however, was substantially unchanged [194].

The possible mechanisms of minimization of prosthesis-derived bone growth inhibitors by shielding of the metal and reduction of the associated metal dissolution was investigated by Ducheyne *et al*. [195]. Ti, Al, and V release rates were determined *in vitro* for Ti-6Al-4V alloy both with and without a CP coating. Surfaces were passivated in 40% HNO_3_ at 55 °C for 20 min. Calcium phosphate ceramic was electrophoretically deposited and subsequently vacuum sintered at 925 °C for 2 h. Immersion for 1, 2, 4, 8, and 16 weeks in Hank’s balanced salt solution, simulated physiological solution with 1.5 mM DS-EDTA. It was found that (i) the CP-coated specimens contained no measurable amounts of Ti, (ii) the Al ion solution around the CP-coated specimen was significantly greater than the concentration around the non-coated specimens; however (iii) Al did not increase significantly with time, at least up to 4 weeks immersion. The CP coating produced a significant increase of biological fixation, yet at the same time a greater Al release into solution, calling into question the value of calcium phosphate ceramic coating in shielding adverse metal passive dissolution to enhance bone growth [195].

Similarly to HA coating, there are various (chemical, electrochemical, and physical) processes for Ca-P coating available. The ions were implanted in sequence, first Ca and then P, both at a dose of 10^17^ ions/cm^2^ at beam energy of 25 keV on CpTi (grade 2). The corrosion tests were done in SBF at 37 °C. It was concluded that (i) the CpTi surface subjected to two-step implantation of Ca+P at a dose of 10^17^ ions/cm^2^ becomes amorphous, (ii) the implantation of Ca+P ions increases the corrosion resistance in SBF exposure at 37 °C for up to 3,200 h, (iii) during exposures to SBF, CP precipitates form the implanted as well as non-implanted samples, (iv) the CP precipitates have no effect on the corrosion resistance, and (v) under the conditions of the applied examination, the biocompatibility of Ti subjected to the two-step implantation of Ca+P ions was similar to that of untreated sample [196]. CpTi (grade 2) were surface-modified by anodic spark discharge anodization and a thin layer (ca. 5 μm) of amorphous TiO_2_ containing Ca and P - Ti/AM. Some of the Ti/AM samples were further modified by hydrothermal treatment to produce a thin outermost (ca. 1 μm) of HA (Ti/AM/HA). It was reported that (i) non-anodized vacuum annealed and hydrofluoric acid etched samples, used as control material, showed good bone adsorption producing Ti excellent surface properties, (ii) anodization at a voltage of 275 V that produced a crack free amorphous TiO_2_ film containing Ca and P (Ti/AM) provided good results for cytocompatibility, important morphological characteristics (micropores without crack development) but presented the lowest bone apposition probably due to the degradation of amorphous TiO_2_ film, and (iii) hydrothermal treatment at 300 °C for 2 h that produced a sub-micrometer layer of HA crystals (ca. 1 μm) upon the amorphous TiO_2_ film (Ti/AM/HA) gave rise to the highest bone apposition only at 8 weeks [81].

Coating by a RF magnetron sputter technique for the production of thin Ca-P coatings can be produced that vary in Ca/P ratio as well as structural appearance. Jansen *et al*. [197] studied the effect of non-coated titanium and three different Ca-P sputtered surfaces on the proliferation and differentiation (morphology and matrix production) of osteoblast-like cells. It was found that (i) proliferation of the osteoblast-like cells was significantly higher on non-coated than on Ca-P coated samples, (ii) on the other hand, more mineralized extracellular matrix was formed on the coated surfaces, and (iii) TEM confirmed that the cells on the coated substrates were surrounded by extracellular matrix with collagen fibres embedded in crystallized needle shaped structures. On the basis of these findings, it was concluded that (i) the investigated Ca-P sputter coatings possess the capacity to activate the differentiation and expression of osteogenic cells, and (ii) bone formation proceeds faster on Ca-P surfaces than on Ti substrates. Further, it was noticed that this bone stimulating effect appeared to be independent of the Ca-P ratio of the deposited coatings [197].

Hayakawa *et al*. [198] prepared four types of Ti implants: (1) as-blasted with titania (<150 μm), (2) sintered with Ti beads with 50–150 μm diameter, (3) blasted with IBDM (ion beam dynamic mixing) Ca-P coating, and (4) multilayered sintered implants with IBDM Ca-P coating. The Ca-P coating was rapid heat-treated with infrared radiation at 700 °C. The implants were inserted into the trabecular bone of the left and right femoral condyles of 16 rabbits, for 2, 3, 4, and 12 weeks. It was reported that (i) histological evaluation revealed new bone formation around different implant materials after already 3 weeks of implantation, (ii) after 12 weeks, mature trabecular bone surrounded all implants, and (iii) the combination of surface geometry and Ca-P coating benefits the implant-bone response during the healing phase [198].

Biomimetic deposition and electrochemical deposition in solution were used for CP deposition on sintered CpTi bead-cylinders (1,300 °C for 2 h under vacuum). The supersaturated solution for CP deposition was prepared by dissolving NaCl, CaCl_2_, Na_2_HPO_4_·2H_2_O in distilled water buffered to pH 7.4. Biomimetic deposition was done by immersing samples in solution at 37 °C for 1, 2, 4, and 6 days, while electrochemical deposition was performed at −1.8 V (*vs*. Hg_2_SO_4_/Hg reference) for 2 h at 37 °C. It was reported that (i) a pre-coating alkaline treatment (5 mol/L NaOH at 60 °C for 5 h) is necessary to obtain a uniform coating layer on the inner pore surfaces when the biomimetric deposition is used, (ii) electrochemical deposition is more efficient and less sensitive to the conditions of the Ti surfaces compared to the biomimetric depositions; however (iii) the electrochemical deposition produces less uniform and thinner coating layers on the inner pore surfaces compared with the biomimetric deposition, and (iv) the crystal structure of the deposited Ca-P is octacalcium phosphate regardless of the deposition methods [199]. Heimann *et al*. [200] deposited hydroxyapatite and duplex hydroxyapatite + titania bond coat layers onto Ti-6Al-4V substrates by atmospheric plasma spraying at moderate plasma enthalpies. From as-sprayed coatings and coatings incubated in simulated body fluid, electron-transparent samples were generated by focused ion beam excavation. It was found that (i) adjacent to the metal surface a thin layer of amorphous calcium phosphate (CP) was deposited, (ii) after *in vitro* incubation of duplex coatings for 24 weeks, Ca-deficient defect apatite needles precipitated from amorphous CP, and (iii) during incubation of hydroxyapatite without a bond coat for 1 week diffusion bands were formed within the amorphous CP of 1–2 μm width parallel to the interface metal/coating, presumably by a dissolution-precipitation sequence [200]. Aluminoborosilicate glass + HA powder were coated on CpTi at 800–900 °C. It was found that (i) the precipitates were Ca-deficient carbonate with low crystallinity, (ii) both morphologies and composition of the precipitates *in vivo* were similar to those *in vitro*, and (iii) the HA particles on the surface of the composite act as nucleation sites for precipitation in physiologic environments, whereas the glass matrix is independent of it [201].

For the same purpose as done for the HA-coating, a Ca-P coated layer is needed to post-coating heat treatment. Lucas *et al*. [202] investigated the post-deposition heat treatments of ion beam as-sputtered coatings by varying the time and temperature. Ca-P coatings, deposited using a hydroxyapatite-fluorapatite target, received the post-deposition heat treatments as follows: 150, 300, 400, 500, and 600 °C for either 30 min or 60 min, followed by furnace cooling to room temperature. It was found that (i) the average bond strengths for the coatings heated to 500 °C (30 min), 600 °C (30 min) and 600 °C (1 h) were 40.1 MPa, 13.8 MPa and 9.1 MPa, respectively. The 500 °C heat treatment for 30 minute resulted in an HA-type coating without reducing the tensile bond strength of the coating. Thus, temperature and time are critical parameters in optimizing coating properties [202], suggesting that the diffusion process must be significantly involved.

Gan *et al*. [203] evaluated the interface shear strength of Ca-P thin films applied to Ti-6Al-4V substrates using a substrate straining method - a shear lag model. The Ca-P films were synthesized using sol-gel methods from either an inorganic or organic precursor solution. Strong interface bonding was demonstrated for both film types. It was reported that (i) the films were identified as non-stoichiometric hydroxyapatite, but with different Ca/P ratios, (ii) the Ca-P films were 1–1.5 μm thick, and (iii) the shear strength was approximately 347 and 280 MPa for inorganic and organic route-formed films, respectively [203]. NiTi was coated with CP by dipping in oversaturated CP solution, with layer thickness (5–20 μm). The porous nature of the layer makes it mechanically stable enough to withstand both the shape memory transition upon cooling and heating and also strong bending of materials (superelasticity). The adhence of human leukocytes and platelets to the AP layer was analyzed *in vivo*. By comparison, in non-coated SME-NiTi, it was reported that leukocytes and platelets showed a significantly increased adhesion to the coated NiTi [204]. Fend *et al*. [205] pre-treated CpTi: by immersing in boiling Ca(OH)_2_ solution for 30–40 min to have only Ca-added pre-calcification, immersing in 20% H_3_PO_4_ solution at 85–95 °C for 30 min to have only P-added pre-phosphatization, and immersed these pre-calcified CpTi in supersaturated CP solution at 37 °C for 1 week. It was found that (i) the osteoblast amount and activity on the surfaces containing Ca are higher than those on the surface containing sole P ions, (ii) Ca^2+^ ion sites on the material surfaces favor protein adsorption, such as fibronectin or vitronectin as ligands of osteoblast, onto the surface due to positive electricity, chemical, and biological function, and (iii) on the AP surfaces, Ca^2+^ ions are the active sites of the osteoblast adhesion and also promote the cell adhesion on PO_4_^3–^ ion sites [205].

As we have seen above, there are many evidence indicating that, *in vitro* tests, calcium phosphate is precipitated on such surfaces when they are immersed in a simulated physiological solution, suggesting a main reason for excellent biocompatibility (e.g., [181]). On the other hand, there are evidence suggesting that, *in vivo*, after even seven day implantation of such modified Ti in rat bone, it was found that Ca/P ratio reduced less than 1 (*i.e.*, Ca-depletion) [206].

#### 3.4.4. Composite Coating

Bioactive calcium phosphate (CaP) coatings were produced on titanium by using phosphate-based glass (P-glass) and hydroxyapatite (HA), and their feasibility for hard tissue applications was addressed *in vitro* by Kim *et al*. [207]. P-glass and HA composite slurries were coated on Ti under mild heat treatment conditions to form a porous thick layer, and then the micropores were filled in with an HA sol-gel precursor to produce a dense layer. The resultant coating product was composed of HA and calcium phosphate glass ceramics, such as tricalcium phosphate (TCP) and calcium pyrophosphate (CPP). It was reported that the coating layer had a thickness of approximately 30–40 μm and adhered to the Ti substrate tightly, (ii) the adhesion strength of the coating layer on Ti was as high as about 30 MPa, (iii) the human osteoblastic cells cultured on the coatings produced by the combined method attached and proliferated favorably, and (iv) the cells on the coatings expressed significantly higher alkaline phosphatase activity than those on pure Ti, suggesting the stimulation of the osteoblastic activity on the coatings [207]. Maxian *et al*. [208] evaluated the effect of amorphous calcium phosphate and poly-crystallized (60% crystalline) HA coatings on bone fixation of smooth and rough (Ti-6Al-4V powder sprayed) Ti-6Al-4V implants after four and 12 weeks of implantation in a rabbit trascortical femoral model. Histological evaluation of amorphous *versus* poorly crystallized HA coatings showed significant differences in bone apposition and coating resorption that were increased within cortical compared to cancellous bone. The poorly crystallized HA coatings showed the most degradation and least bone apposition. Mechanical evaluation, however, showed no significant differences in push-out shear strengths. Significant enhancement in interfacial shear strengths for bioceremic coated, as compared to uncoated implants, was seen for smooth-surfaced implants (3.5–5 times greater) but not for rough-surfaced implants at four and 12 weeks. Based on these results, it was suggested that once early osteointegration is achieved biodegradation of a bioactive coating should not be detrimental to the bone/coating/implant fixation [208]. Plasma sprayed HA coatings on titanium alloy substrates have been used extensively due to their excellent biocompatibility and osteoconductivity. However, the erratic bond strength between HA and Ti alloy has raised concern over the long-term reliability of the implant. Accordingly, Khor *et al*. developed HA/yttria-stabilized-zirconia (YSZ)/Ti-6Al-4V composite coatings that possess superior mechanical properties to conventional plasma sprayed HA coatings [209]. Ti-6Al-4V powders coated with fine YSZ and HA particles were prepared through a unique ceramic slurry mixing method. The composite powder was employed as feedstock for plasma spraying of the HA/YSZ/Ti-6Al-4V coatings. The influence of net plasma energy, plasma spray standoff distance, post-spray heat treatment on microstructure, phase composition, and mechanical properties were investigated. It was found that (i) coatings prepared with the optimum plasma sprayed condition showed a well-defined splat structure, (ii) HA/YSZ/Ti-6Al-4V solid solution was formed during plasma spraying, which was beneficial for the improvement of mechanical properties, (iii) the microhardness, modulus of elasticity, fracture toughness, and bond strength increased significantly with the addition of YSZ, and (iv) post-spray heat treatment at 600 °C and 700 °C for up to 12 h was found to further improve the mechanical properties of coatings [209]. Yttria stabilized zirconia (YSZ) is often used as reinforcement for many ceramics because it has the merits of high strength and enhanced toughening characteristics during crack-particle interactions [210–213].

Yamada *et al*. [214] utilized the Cullet method for which (1) the mixture of HA powder and glass frits are sintered at 900–1000 °C for from 5 to 10 min to prepare well homogenized coating powder, whereas the conventional method is just mixing and not sintering, and (2) the time of etching treatment, through which the bioactive surface is formed using the mixed solution of HNO_3_ and HF, is relatively short (within 1 min) compared to the conventional method. Through this method, functionally gradient HA/Ti composite implants were successfully fabricated with higher quality compared with the conventional method [214]. Suzuki *et al*. [215] coated titanium dioxide onto silicone substrates by radio-frequency sputtering. It was reported that silicone coating with titanium dioxide enhanced the breakdown of peroxynitrite by 79%. Titanium dioxide-coated silicone inhibited the nitration of 4-hydroxy-phenylacetic acid by 61% compared to aluminum oxide-coated silicone and 55% compared to uncoated silicone. Titanium dioxide-coated silicone exhibited a 55% decrease in superoxide compared to uncoated silicone, and a 165% decrease in superoxide compared to uncoated polystyrene [215]. Titanium dioxide-coated silicone inhibited IL-6 production by 77% compared to uncoated silicone. Based on these findings, it was concluded that the anti-inflammatory properties of titanium dioxide can be transferred to the surfaces of silicone substrates [215].

HA coatings with titania addition were produced by the high velocity oxy-fuel spray process by Li *et al*. [216]. It was found that (i) the addition of TiO_2_ improves the MOE, fracture toughness, and shear strength of high velocity oxy-fuel sprayed HA-based coatings, (ii) the incorporation of the secondary titania phase is found to have a negative effects on the adhesive strength of high velocity oxy-fuel sprayed HA coatings, (iii) the titania is found to inhibit the decomposition of HA at evelated temperatures lower than 1,410 °C, at which point the mutual chemical reaction occurs, and (iv) a small amount of TiO_2_ added into high velocity oxy-fuel sprayed HA coatings with less than 20 vol% is therefore recommended for strengthening of HA-coatings [216]. Lu *et al*. [217] fabricated a two-layer hydroxyapatite (HA)/HA + TiO_2_ bond coat composite coating (HTH coating) on titanium by the plasma spraying technique. The HA + TiO_2_ bond coat (HTBC) consists of 50 vol% HA and 50 vol% TiO_2_ (HT). The as-sprayed HT coating consists mainly of crystalline HA, rutile TiO_2_ and amorphous Ca-P phase, but the post-spray heat treatment at 650 °C for 120 min effectively restores the structural integrity of HA by transforming non-HA phases into HA [216]. It was found that there exists interdiffusion of the elements within the HTBC, but no chemical product between HA and TiO_2_, such as CaTiO_3_ was formed. The toughening and strengthening mechanism of HTBC is mainly due to TiO_2_ as obstacles resisting cracking, and the reduction of the near-tip stresses resulting from stress-induced microcracking [217]. Ng *et al*. [218] mimicked bio-mineralization of bone by applying an initial TiO_2_ coating on Ti-6Al-4V by electrochemical anodization in two dissimilar electrolytes, followed by the secondary calcium (CaP) coating, subsequently applied by immersing the substrates in a simulated body fluid (SBF) with three times concentration (SBF × 3). Electrochemical impedance spectroscopy (EIS) and DC potentiodynamic polarization assessments in SBF revealed that the anodic TiO_2_ layer is compact, exhibiting up to a four-fold improvement in *in vitro* corrosion resistance over unanodised Ti-6Al-4V. X-ray photoelectron spectroscopy analysis indicates that the anodic Ti oxide is thicker than air-formed ones with a mixture of TiO_2–_*_x_* compound between the TiO_2_/Ti interfaces. The morphology of the dense CaP film formed, when observed using scanning electron microscopy, is made up of linked globules 0.1–0.5 μm in diameter without observable delamination. It was also found that (i) the calculated Ca:P ratios of all samples (1.14–1.28) are lower than stoichiometric hydroxyapatite (1.67), and (ii) a duplex coating consisting of a compact TiO_2_ with enhanced *in vitro* corrosion resistance and bone-like apatite coating can be applied on Ti-6Al-4V by anodization and subsequent immersion in SBF [218].

Knabe *et al*. [219] investigated the effects of novel calcium titanium, calcium, titanium zirconium phosphates suitable for plasma spraying on CpTi substrate on the expression of bone-related genes and proteins of human bone-derived cells, and compared the effects to that on native Ti and HA-coated Ti. Test materials were acid etched and sand-blasted, plasma-sprayed HA, and sintered CaPO_4_ with Ti, Zr, TiO_2_, and ZrO_2_. Human bone-derived cells were grown on these surfaces for 3, 7, 14, and 21 days, counted and probed for various mRNAs and proteins. It was reported that (i) all surfaces significantly affected cellular growth and the temporal expression of an array of bone-related genes and proteins, (ii) at 14 and 21 days, cells on sintered displayed significantly enhanced expression of all osteogenetic mRNAss, and (iii) surfaces of 55CaO·20TiO_2_·31P_2_O_5_ and CaTi_4_(PO_4_)_6_ had the lost effect on osteoblastic differentiation inducing a greater expression on an array of osteogenetic markers than recorded for cells grown on HA, suggesting that these novel materials may possess a higher potency to enhance osteogenesis [219]. Shtansky *et al*. [220] performed a comparative investigation of multicomponent thin films based on the systems Ti-Ca-C-O-(N), Ti-Zr-C-O-(N), Ti-Si-Zr-O-(N) and Ti-Nb-C-(N). TiC_0.5_ + 10%CaO, TiC_0.5_ + 20%CaO, TiC_0.5_ + 10%ZrO_2_, TiC_0.5_ + 20%ZrO_2_, Ti_5_Si_3_ + 10%ZrO_2_, TiC_0.5_ + 10%Nb_2_C and TiC_0.5_ + 30%Nb_2_C composite targets were manufactured by means of self-propagating high-temperature synthesis, followed by DC magnetron sputtering in an atmosphere of argon or in a gaseous mixture of argon and nitrogen. The biocompatibility of the films was evaluated by both *in vitro* and *in vivo* experiments. The *in vitro* studies involved the investigation of the proliferation of Rat-1 fibroblasts and IAR-2 epithelial cells on the tested films, morphometric analysis and actin cytoskeleton staining of the cells cultivated on the films. *In vivo* studies were fulfilled by subcutaneous implantation of Teflon plates coated with the tested films in mice and analysis of the population of cells on the surfaces. It was reported that (i) the films showed high hardness in the range of 30–37 GPa, significant reduced modulus of elasticity, low friction coefficient down to 0.1–0.2, and low wear rate in comparison with conventional magnetron-sputtered TiC and TiN films, (ii) no statistically significant differences in the attachment, spreading, and cell shape of cultured IAR-2 and Rat-1 cells on the Ti-Ca-C-O-(N), Ti-Zr-C-O-(N) (TiC_0.5_+10%ZrO_2_ target), Ti-Si-Zr-O-(N) films and the uncoated substrata was detected, and (iii) the adhesion and proliferation of cultured cells *in vitro* was perfect at all investigated films. Based on these findings, it was concluded that the combination of excellent mechanical properties with non-toxicity and biocompatibility makes Ti-Ca-C-O-N, Ti-Zr-C-O-N, and Ti-Si-Zr-O-N films promising candidates as tribological coatings to be used for various medical applications like total joint prostheses and dental implants [220]. Von Walter *et al*. [221] introduced a porous composite material, named “Ecopore”, and described *in vitro* investigation of the material and its modification with fibronectin. The material is a sintered compound of rutile TiO_2_ and the volcanic silicate perlite with a macrostructure of interconnecting pores. In an *in vitro* model, human primary osteoblasts were cultured directly on Ecopore. It was reported that human osteoblasts grew on the composite as well as on samples of its single constituents, TiO_2_ and perlite glass, and remained vital, but cellular spreading was less than on tissue culture plastic. To enhance cellular attachment and growth, the surface of the composite was modified by etching, functionalization with aminosilane and coupling of fibronectin, resulting in greatly enhanced spreading of human osteoblasts. It was therefore concluded that (i) Ecopore is non-toxic and sustains human osteoblasts growth, cellular spreading being improvable by coating with fibro-nectin, and (ii) the composite may be usable in the field of bone substitution [221].

Different biomaterials have been used as scaffolds for bone tissue engineering. Rodrigues *et al*. [222] characterized biomaterial composed of sintered (at 1,100 °C) and powdered hydroxyapatite and type I collagen (both of bovine origin) designs for osteoconductive and osteoinductive scaffolds. Collagen/HA proportions were 1/2.6 and 1/1 by weight, with particle sizes ranging from 200 to 400 μm. X-ray diffraction and infrared spectroscopy showed that the sintered (1,100 °C) bone was composed essentially of HA with minimum additional groups as surface calcium hydroxide, surface and crystal water, free carbon dioxide, and possibly brushite. It was reported that osteoblasts adhered and spread on both the HA particle surface and the collagen fibers, which seemed to guide cells between adjacent particles [222], suggesting that this biocomposite can be considered as ideal for its use as scaffold for osteoconduction and osteoinduction. Cheng *et al*. [223] prepared electrochemically a bovine serum albumin (BSA) protein-containing AP coating on a HA coated Ti-6Al-4V. It was reported that (i) the method resulted in a 70-fold increase in BSA inclusion compared to simple adsorption, and was subsequently released by a slow mechanism (15% loss over 70 h), and (ii) thus, this technique provides an efficient method of protein incorporation at physiological stem, with a potential for sustained release of therapeutic agents, as may be required for metallic implant fixation [223].

Redepenning *et al*. [224] prepared another type of biocomposite coatings containing brushite (CaHPO_4_·2H_2_O) and chitosan by electrochemical deposition. The brushite/chitosan composites were converted to hydroxyapatite/chitosan composites in aqueous solutions of sodium hydroxide. The coatings ranged from about 1 to 15% chitosan by weight. It was mentioned that qualitative assessment of the coatings showed adhesion significantly improved over that observed for electrodeposited coatings of pure HA [224].

#### 3.4.5. TiN Coating

In spite of their high strength, low density, and good corrosion resistance, the usefulness of Ti alloys in general engineering components is frequently limited by their poor wear resistance. If the alloy surface is subjected to conditions of sliding or fretting, adhesive wear can rapidly lead to catastrophic failure unless appropriate surface engineering is carried out. In order to combat modest contact loads, several surface treatments are commercially available, such as plasma nitriding or PVD coating with TiN. Titanium nitride is known for its high surface hardness and mechanical strength. It was also reported that the dissolution of Ti ions is very low [225]. As for dental implants, they are comprised of various components. The implant abutment part (the mucosa penetration part) is exposed in the oral cavity, and so plaque and dental calculus easily adhere on it. Removal of the plaque and dental calculus is necessary to obtain a good prognosis throughout the long term maintenance of the implant. Based on this background, Kokubo *et al*. [226] prepared CpTi (grade 1) samples by polishing with #2000grit paper, or buff-polishing with 6 μm diamond emulsion paste, followed by a 0.1% HF acid solution for 10 s to clean the surface, then treated in N_2_ atmosphere of 1 atm at 850 °C for 7 h (N_2_ flow rate: 50 L/min). It was reported that (i) the nitrided layer about 2 μm thick composed of TiN and Ti_2_N was formed on Ti by a gas nitriding method, and the dissolved amount of Ti ion in SBF (simulated body fluid) was as low as the detectable limit of ICP-MS (Inductively Coupled Plasma Mass Spectroscopy), and that the 1% lactic acid showed no significant difference from Ti [226]. SBF, in genral, consists of Na^+^ (142.0), K^+^ (5.0), Mg^2+^ (1.5), Ca^2+^ (2.5), Cl^−^(148.8), HCO_3_^−^ (4.2), HPO_4_^−^ (1.0). H.P. Na (142.0), K (5.0), Mg (1.5), Ca (2.5), Cl (103.0), HCO_3_ (13.5), and HPO_4_ (1.0).

Surface topography and chemistry have been shown to be extremely important in determining cell-substrate interactions and influencing cellular properties such as cell adhesion, cell-cell reactions, and cytoskeletal organization [227]. The cell-substrate interaction of primary hippocampal neurones with thin films of TiN was studied *in vitro*. TiN films of different surface chemistries and topographies were deposited by pulsed DC reactive magnetron sputtering and closed field unbalanced magnetron sputter ion plating to result in TiN thin films with similar surface chemistries, but different topographical features. It was reported that (i) primary hippocampal neurones were found to attach and spread to all of the TiN films, (ii) neuronal network morphology appeared to be more preferential on the nitrogen rich TiN films, and also reduced nanotopographical features, (iii) at early time points of one and four days *in vitro* primary hippocampal neurones respond to the presence of interstitial nitrogen rather than differences in nanotopography; however (iv) at seven days more preferential neutronal network morphology is formed on TiN thin films with lower roughness values and decreased size of topographical features [227]. Bull *et al*. [228] studied the use of thin titanium interlayers to promote the adhesion of TiN coatings on a range of substrates. For thin interlayers, an interstitially hardened titanium layer is formed at the interface, resisting the interfacial crack propagation. However, at a critical interlayer thickness, the surface contaminants are completely dissolved in the interfacial layer, and depositing any further titanium leads to an overall softer interfacial layer which offers less resistance to crack propagation, and delamination can easily take place. For this reason, failure is observed within the interlayer for thick interlayers, whereas it occurs at the interlayer/substrate interface for thinner interlayers. Another contribution to the enhanced adhesion comes from the reduction in coating stresses in the interfacial region due to the presence of a soft compliant layer, which was examined by changing the hardness of the interlayer deposited before coating deposition. It was concluded that (i) softer interlayers do not lead to improved adhesion performance in most cases, and (ii) it appears that the best adhesion results from a hard interlayer that leads to ductile failure at the coating/substrate interface, rather than the brittle failure observed due to the presence of oxide films [228].

Mechanical-electrochemical interactions accelerate corrosion in mixed-metal modular hip prostheses. These interactions can be reduced by improving the modular component machining tolerances or by improving the resistance of the components to scratch or fretting damage. Wrought Co-alloy (Co-Cr-Mo) is known to have better tribological properties compared to the Ti-6Al-4V alloy. Thus, improving the tribological properties of this mixed-metal interface should center on improving the tribological properties of Ti-6Al-4V. It was mentioned that (i) the nitrogen-diffusion-hardened Ti alloy samples had a more pronounced grain structure, more nodular surface, and significantly higher mean roughness values than the control Ti-6Al-4V, (ii) the nitrided Ti-6Al-4V samples also exhibited at least equivalent corrosion behavior and a definite increases in surface hardness compared to the control Ti-6Al-4V samples, and (iii) fretting can be reduced by decreasing micromotion or by improving the tribological properties (wear resistance and surface hardness) of the material components at this interface [229]. The corrosion behavior of the titanium nitride-coated TiNi alloy was examined in 0.9% NaCl solution by potentiodynamic polarization measurements and a polarization resistance method [230]. XPS spectra showed that the titanium nitride film consisted of three layers, a top layer of TiO_2_, a middle layer of TiN_x_ (x > 1), and an inner layer of TiN, which agreed very well with results obtained by Oshida *et al*. [231]. The passive current density for the titanium nitride-coated alloy was approximately two orders of magnitude lower than that of the polished alloy in the potential range from the free corrosion potential to +500 mV (*vs*. Ag/AgCl). Pitting corrosion associated with breakdown of the coated film occurred above this potential. The polarization resistance data also indicated that the corrosion rate of the titanium nitride-coated alloy at the corrosion potential (+50 to +100mV) was more than one order of magnitude lower than that for the polished alloy. It was concluded that the corrosion rate of TiNi alloy can be reduced by more than one order of magnitude by titanium nitride coating, unless the alloy is highly polarized anodically *in vivo* [230]. Bordji *et al*. [232] prepared Ti alloys treated by: (1) glow discharge nitrogen implantation (10^17^ atoms cm^−2^), (2) plasma nitriding by plasma diffusion treatment, and (3) deposition of TiN layer by plasma-assisted chemical vapour deposition additionally to plasma diffusion treatment. A considerable improvement was noticed in surface hardness, especially after the two nitriding processes. A cell culture model using human cells was chosen to study the effect of such treatments on the cytocompatibility. The results showed that Ti-5Al-2.5Fe alloy was as cytocompatible as the Ti-6Al-4V alloy, and the same surface treatment led to identical biological consequences on both alloys. It was concluded that (i) after the two nitriding treatments, cell proliferation and viability appeared to be significantly reduced and the SEM revealed somewhat irregular surface states; however (ii) osteoblast phenotype expression and protein synthesis capability were not affected [232]. Goldberg *et al*. [233] utilized the plasma vapor deposition technique, by which the samples were placed into a vacuum chamber and sputtered to remove the oxide film, followed by depositing a 200 nm thick interlayer of titanium to enhance the coating/substrate interface. Alternating layers of TiN and AlN were deposited until a coating thickness of approximately 5 μm was produced. The mechanical and electro-chemical behavior of the surface oxides of Co-Cr-Mo and Ti-6Al-4V alloys during fracture and repassivation play an important role in the corrosion of the taper interfaces of modular hip implants. These corrosion properties were investigated in one group of Co-Cr-Mo and Ti-6Al-4V alloy samples passivated with nitric acid and another group coated with TiN/AlN coating. It was found that (i) Co-Cr-Mo had a stronger surface oxide and higher interfacial adhesion strength, making it more resistant to fracture than Ti-6Al-4V, (ii) if undistributed, the oxide on the surface of Ti-6Al-4V significantly reduced dissolution currents at a wider range of potential than Co-Cr-Mo, making Ti-6Al-4V more resistant to corrosion, (iii) the TiN/AlN coating had higher hardness and modulus of elasticity than Co-Cr-Mo and Ti-6Al-4V. It was much less susceptible to fracture, had higher interfacial adhesion strength, and was a better barrier to ionic diffusion than the surface oxides on Co-Cr-Mo and Ti-6Al-4V [233].

The most of surface treatments such as plasma nitriding or PVD coating with TiN are, however, carried out in the solid state and the depth of coating or hardening is restricted by low diffusivity. The diffusion coefficient of nitrogen in Ti is more than a thousand times slower than that in steels due to different crystalline structures. The packing factor of Ti (HCP) is 74%, while that of steel (BCC) is 68%, so that steel has more spaces available for diffusing species. In order to achieve the depth of hardening necessary to withstand the subsurface Hertzian stresses induced by heavy rolling contact, it is necessary to alloy the Ti surface in the molten state. The necessary depth of surface hardening can readily be achieved in this way by laser melting the surface in the presence of interstitial alloying elements such as carbon, oxygen, and nitrogen. Of these, nitrogen has been found to provide the best balance between increased hardness and decreased ductility, and can easily be added by laser gas nitriding. The Hertzian compressive stress in the substrate was increased to 1.36 GPa [234]. Pure iron has allotropic phase transformations: the first one is 910 °C between α-BCC and γ-FCC, and the second one is 1,390 °C between γ-FCC and δ-BCC. While investigating the deformation mechanism of transformation superplastivity, Oshida observed that the transformation front behaves as if sime-liquid due to loosing a clear crystalline electorn diffraction pattern even both α-BCC and γ-FCC are still in solid state. Based on this finding, the “semi-liquid trans-formation front” model was proposed. One of various applications using transformation superplasticity is a nitridation of metallic materials if they have an allotropic phase transformation temperature. It was demonstrated that CpTi was succeesfully nitrided when CpTi was heated and cooled repeatedly passing the β-transus temperature (between 800 °C and 930 °C) for several times in nitrogen gas filled chamber [231]. Ion implantation, diffusion hardening, and coating are surface modification techniques for improving the wear resistance and surface hardness of Ti alloy surfaces [235–239]. A Ti-6Al-4V sample was diffusion-hardened in a nitrogen atmosphere for 8 h at 566 °C and argon or nitrogen quenched to room temperature. The nitrogen-diffusion-hardened Ti-6Al-4V had TiN and TiNO complexes at the immediate surface and sub-surface layers. The diffusion-hardened samples also had a deeper penetration of oxygen compared to regular Ti-6Al-4V samples [240].

As briefly described in the above, Oshida *et al*. [231,241] applied a TiN coating onto CpTi substrate prior to porcelain firing to develop a new method to control the excessive oxidation. The bonding strength of porcelain to metals depends on the oxide layer between the porcelain and the metal substrate. Oxidation of a metal surface increases the bonding strength, whereas excessive oxidation decreases it. Titanium suffers from its violent reactivity with oxygen at high temperatures that yield an excessively thick layer of TiO_2_, and this presents difficulties with porcelain bonding. The oxidation kinetics of titanium simulated to porcelain firing was investigated, and the surface nitridation of CpTi as a process of controlling the oxidation behavior was evaluated. Nitrided samples with the arc ion plating PVD process and un-nitrided control CpTi were subjected to oxidation simulating of Procera porcelain with 550, 700, and 800 °C firing temperatures for 10 min in both 1 and 0.1 atmospheric air. The weight difference before and after oxidation was calculated, and the parabolic rate constant, K_p_ (mg^2^/cm^4^/s), was plotted against inverse absolute temperature (*i.e.*, in an Arrhenius plot). Surface layers of the samples were subjected to x-ray and electron diffraction techniques for phase identifications. Results revealed that both nitrided and un-nitrided samples obey a parabolic rate law with activation energy of 50 kcal/mol. In addition, the study shows that nitrided CpTi had a K_p_ about 5 times lower than the un-nitrided CpTi, and hence the former needs 2.24 times longer oxidation time to show the same degree of oxidation. Phase identification resulted in confirming the presence of TiO_2_ as the oxide film in both groups, but with 1–2 μm thickness for the un-nitrided CpTi and 0.3–0.5 μm thickness for nitrided samples. Therefore, it can be concluded that nitridation of titanium surfaces can be effective in controlling the surface oxide thickness that might ensure satisfactory bonding with porcelain [231]. Oshida *et al*. [241] evaluated CpTi substrates subjected to porcelain firing and bond strengths under three-point bending mode (span length: 15mm; crosshead speed: 0.5 mm/min). Experimental variables included surface treatments of CpTi and porcelain firing schedules. Variables for the surface treatments were: (1) sandblasting, (2) mono- and triple-layered nitridation, and (3) mono-layered chrome-doped nitridation. Variables for the porcelain firing schedule included (4) bonding agent application, (5) bonding agent plus gold bonding agent application, and (6) Procera porcelain application. Statistically, all of them exhibited no significant differences. Hence, we employed two further criteria: (i) the minimum bond strength should exceed the maximum porcelain strength *per se*, and (ii) the CpTi substrate should not be heated close to the beta-transus temperature. After applying these criteria, it was concluded that mono-layered nitridation and mono-layered application of chrome-doped nitridation on both (with and without) sand-blasted and non-sand-blasted surfaces were the most promising conditions for a successful titanium-porcelain system [241]. It seems that an alloy which has the properties of titanium and is relatively inexpensive would be a very good material for surgical purposes. These requirements could be met, for example, by stainless steel coated with a firmly adhering non-porous titanium film. Głuszek *et al*. [242] coated 316L (18Cr-8Ni-2Mo with low carbon content) stainless steel with Ti or TiN by ion plating. The galvanic effects for the galvanic couples 316L/Ti, 316L/Ti-coated 316L, 316L/TiN-coated 316L were studied in Ringer’s solution. It was concluded that (i) both Ti and TiN coatings were non-porous, (ii) Ti served as an anode in the couple 316L/Ti, whereas for the other two couples, the coatings were the cathodes; however (iii) the dissolution rate of 316L stainless steel in these couples was smaller than expected owing to a strong polarization of the coatings [242].

For hip prostheses, the coupling between the metallic femoral head and the polymeric acetabular cup is normally used. Biotribiological phenomena contribute principally to the clinical failure of the prosthesis. In the metal-polymer coupling, the problems consist of biotribological wear, creep of the UHMWPE (ultra high molecular weight polyethylene), and fretting corrosion of the metal femoral head. Cyclic stress exceeding the fatigue resistance of UHMWPE produces surface microcracks and particulates that can migrate into the tissue of the host implant. This fretting-wear debris causes local irritation, proliferation of fibrous tissue, and necrosis of bone. Minimizing the wear is critically important for maintaining the long life of the femoral prosthesis. On the other hand, titanium alloys are susceptible to fretting corrosion; this susceptibility can be reduced via surface treatments. UHMWPE was gamma-ray sterilized. This sterilization technique results in a cross-linking of the polymer, which enhances its wear resistance. Tribio-logical behavior of N_2_-implanted and nonimplanted titanium alloys coupled with UHMWPE were studied using pin-on-flat tests, according to ASTM F 732-82 in bovine serum. The results show that while the non-implanted titanium alloy and the titanium with N_2_ on UHMWPE resulted in high final wear values, titanium implanted with O_2_ generates a wear value less than that obtained for polyethylene against 316L stainless steel. Ti-6Al-4V implanted with chromium exhibited the lowest wear. Hardness values of the implanted material corresponded to the wear rates, which assist in determining optimal elements for implantation. Implantation of certain elements may increase the surface activity, resulting in more adherent oxide layers that also increase wettability [100].

#### 3.4.6. Ti Coating

Lee *et al*. [243] conducted the *in vivo* study to evaluate the behavior and mechanical stability in implants of three surface designs, which were smooth surface Ti, rough Ti surface by plasma spray coating, and alkali and heat-treated. The implants were inserted transversely in a dog thighbone and evaluated at 4 weeks of healing. At four weeks of healing after implantation in bone, it was found that (i) the healing tissue was more extensively integrated with an alkali and heat-treated Ti implant than with the implants of smooth surface and/or rough titanium surfaces, (ii) the bone bonding strength (pull-out force) between living bone and implant was observed by a universal testing machine, (iii) the pull-out forces of the smooth surface Ti, plasma spray coated Ti, and alkali and heat-treated Ti implants were 235, 710, and 823 N, respectively, and (iv) histological and mechanical data demonstrated that appropriate surface design selection can improve early bone growth and induce an acceleration of the healing response, thereby improving the potential for implant osseointegration. In order to improve the biocompatibility of functional titanium-based alloys, Sonoda *et al*. [244] investigated pure titanium coatings on Ti-6Al-4V alloy by sputtering. More high quality thin film and higher growth rate were obtained by the sputtering with a DC source than with an RF source. After the cleaning method was established, the effect of sputtering on the thickness of the film was investigated with DC sputtering. It was concluded that the growth rate of sputtered titanium film was proportional to the applied electric power, and the orientation of the film highly depended on the heating temperature of the substrate [244]. Sonoda *et al*. [245] further applied this technique to the complete denture base of the Ti-6Al-4V alloy fabricated by superplastic forming. The base was attached to the substrate holder and cooled by water or heated at 417 °C. It was reported that the film deposited on the heated base was superior to that on the cooled one in smoothness, glossiness, uniformity, and covering of the fine cracks [245].

Histologically, Ti has been demonstrated to be a highly biocompatible material on account of its good resistance to corrosion, absence of toxic effects on macrophages and fibroblasts, and lack of inflammatory response in peri-implant tissues [245–249]. Ti endosseous dental screws with different surfaces (smooth Ti, Ti plasma-sprayed, alumina oxide sand-blasted and acid-etched, zirconia (ZrO_2_) sand-blasted and acid-etched) were implanted in femura and tibiae of sheep for 14 days to investigate the biological evolution of the peri-implant tissues and detachment of Ti debris from the implant surfaces in early healing. Implants were not loaded. It was reported that (i) all samples showed new bone trabeculae and vascularized medullary spaces in those areas where gaps between the implants and host bone were visible, (ii) in contrast, no osteogenesis was induced in the areas where the implants were initially positioned in close contact with the host bone, (iii) the threads of some screws appeared to be deformed where the host bone showed fractures, and (iv) Ti granules of 3–60 μm were detectable only in the peri-implant tissues of Ti plasma sprayed implants both immediately after surgery and after 14 days, thus suggesting that this phenomenon may be related to the friction of the Ti plasma spray coating during surgical insertion [250].

The use of porous coated implants for long-term biological fixation has been receiving an enthusiastic response, especially when the patients are younger and more active [251,252]. The application of Ti plasma-sprayed coatings to Ti-6Al-4V orthopedic implants results in a dramatic decrease in high-cycle fatigue performance. It was noted that the better bonding of the plasma sprayed and heat-treated implants results in a lower high-cycle fatigue strength. As with conventional sintered porous coatings, the application of a coating that contains defects serves as the crack initiator of the high cycle fatigue. It was also mentioned that the addition of the post-coating heat treatment to improve coating bonding strength resulted in a further reduction in the high cycle fatigue strength, most likely due to a higher frequency of bonding sites between the coating and substrate, and a more intimate metallurgical bond at those sites [253].

Recently, there was a growing interest in Ti plasma sprayed overcoats as a viable alternative to sintered bead or diffusion-bonded fiber metal surfaces, since the inherent roughness of such coatings is believed to favor the osteointegration of the bone [254,255]. Surface treatment plays an important role in the corrosion resistance of Ti. The cement, in spite of having reduced electrical conductivity in comparison to metal, is an ionic transporter, and therefore capable of participating in the corrosion process. The crevice corrosion at the metal-cement interface was studied by Reclaru *et al*., who reported that in the case of plasma spray surfaces, a process of diffusion of Ti particles in the electrolyte could accompany the crevice corrosion [256].

Xue *et al*. [257] modified plasma-sprayed titanium coatings by an alkali treatment. The changes in chemical composition and structure of the coatings were examined by SEM and AES. The results indicated that (i) a net-like microscopic texture feature, which was full of the interconnected fine porosity, appeared on the surface of alkali-modified titanium coatings, (ii) the surface chemical composition was also altered by alkali modification, and (iii) a sodium titanate compound was formed on the surface of the titanium coating and replaced the native passivating oxide layer. The bone bonding ability of titanium coatings were investigated using a canine model. The histological examination and SEM observation demonstrated that more new bone was formed on the surface of alkali-modified implants, and grew more rapidly into the porosity. It was therefore concluded that (i) the alkali-modified implants appose directly to the surrounding bone, (ii) in contrast, a gap was observed at the interface between the as-sprayed implants and bone, (iii) the push-out test showed that alkali-modified implants had higher shear strength than as-sprayed implants after 1 month of implantation, and (iv) an interfacial layer, containing Ti, Ca, and P, was found to form at the interface between bone and the alkali-modified implant [257].

Borsari *et al*. [258] developed a new implant surface with the purpose of avoiding as much stress shielding as possible, and thus prolong the prosthesis lifespan, and investigated the *in vitro* effect of this new ultra-high roughness and dense Ti (R_a_ = 74 μm) in comparison with medium (R_a_ = 18 μm) and high (R_a_ = 40 μm) roughness and open porous coatings, which were obtained by vacuum plasma spraying. MG63 osteoblast-like cells were seeded on the tested materials and polystyrene, as control, for three and seven days. It was reported that (i) akaline phosphatase activity had lower values for high roughness surfaces than medium and ultra-high rough surfaces, (ii) procollagen-I synthesis reduced with increasing roughness, and the lowest data was found for the ultra-high rough surface, (iii) all tested materials showed significantly higher Interleukin-6 levels than those of polystyrene at both experimental times, and (iv) the new ultra-high roughness and dense coating provided a good biological response, even though, at least *in vitro*, it behaved similarly to the coatings already used in orthopedics [258]. The bone response to different titanium plasma-sprayed implants was evaluated in the trabecular femoral condyles of 10 goats by Vercaigne *et al*. [259]. These implants were provided with three different titanium plasma-sprayed coatings with a R_a_ of 16.5, 21.4, and 37.9 μm, respectively. An Al_2_O_3_ grit-blasted implant with a R_a_ of 4.7 μm was used as a control. After an implantation period of three months, the implants were evaluated histologically and histomorphometrically. Only one implant was not recovered after the evaluation period. It was reported that (i) most of the implants showed a different degree of fibrous tissue alternating with direct bone contact, (ii) complete fibrous encapsulation of the implant was observed in some of the sections, and no signs of delamination of the plasma-sprayed coating was visible, (iii) no significant differences in bone contact were measured between the different types of implants, (iv) hismorphometrical analysis revealed significantly higher bone mass close to the implants (0–500 μm) for treated implants placed in medial femoral condyle and implants placed in the lateral condyle, and (v) at a distance of 500–1500 μm no difference in bone mass measurements between the different implants was observed [259]. Ong *et al*. [260] *in vivo* evaluated the bone interfacial strength and bone contact length at the plasma sprayed HA and Ti plasma sprayed implants. Non-coated Ti implants were used for control. Cylindrical coated or non-coated implants were implanted in the dogs’ mandibles. Loading of the implants was performed at 12 weeks after implantation. At 12 weeks after implantation (prior to loading) and one year after loading, implants were evaluated for interfacial bone-implant strength and bone-implant contact length. It was found that (i) no significant differences in interfacial bone-implant strength for all groups at 12 weeks after implantation and after one year loading in normal bone were found; however (ii) bone contact length for HA implants was significantly higher than the Ti plasma sprayed and Ti implants for both periods tested, and (iii) Ti plasma sprayed implants exhibited similar pull-out strength compared to the HA implants [260].

#### 3.4.7. Titania Film Coating

The high corrosion resistance and good biocompatibility of Ti and its alloys are due to a thin passive film that consists essentially of TiO_2_. There is increasing evidence, however, that under certain conditions, extensive Ti release may occur *in vivo*. An ion-beam-assisted sputtering deposition technique has been used to deposit thick and dense TiO_2_ films on Ti and stainless steel surfaces. A higher electrical film resistance, lower passive current density, and lower donor density (in order of 10^15^ cm^−3^) have been measured for sputter-deposited oxide film on Ti in contrast to the naturally formed passive oxide film on Ti (donor density in the order of 10^20^ cm^−3^). It was found that (i) the coated surface exhibited improved corrosion resistance in phosphate buffered saline, and (ii) the improved corrosion protection of the sputter-deposited oxide film can be explained by a low defect concentration and, consequently, by a slow mass transport process across the film [261]. The National Industrial Research Institute of Nagoya has established technology for forming a functional gradient Ti-O oxide film on Ti-6Al-4V by the reactive DC sputtering vapor deposition method. The overall film thickness in the experiment was 3 μm, and the Vickers hardness of the surface was 1,500 (*vs*. CpTi: 200–300) [262].

The conditions for obtaining titanium dioxide from the substrates titanium tetra-chloride and oxygen, and applying this to a surgical 316L stainless steel by PACVD (Plasma Assisted Chemical Vapor Deposition) were determined. It was established that during the process, Ti dioxide anatase is created. During exposure of the 316L stainless steel with the Ti dioxide coating, in Ringer’s solution, it was found that (i) protective properties of this coating improved, (ii) Ti dioxide covering increased the resistivity of 316L stainless steel to pitting corrosion and general corrosion, and (iii) any damage or partial removal of the coating did not cause an increased galvanic corrosion of the substrate [263].

Wollastonite (CaSiO_3_) ceramic was studied as a medical material for artificial bones and dental roots because of its good bioactivity and biocompatibility [264,265]. Lui *et al*. [266] prepared wollastonite/TiO_2_ composite coating using plasma spraying technology onto Ti-Al6-4V substrate. The composite coating revealed a lamellar structure with alternating wollastonite coatings and TiO_2_ coating. In the case of composite coatings, the primarily crystalline phases of the coatings were wollastonite and rutile, indicating wollastonite and TiO_2_ did not react during the plasma spraying process. It was found that (i) the mean Vickers microhardness of the coatings increased with an increase in the content of TiO_2_. Wollastonite/TiO_2_ composite coatings were soaked in SBF to examine their bioactivity, (ii) a carbonate-containing HA layer was formed on the surface of the wollastonite and composite coatings (wollastonite/TiO_2_: 7/3) soaked in SBF, while a carbonate-containing HA layer was not formed on the surface of the TiO_2_ and composite (with wollastonite/TiO_2_: 3/7) coatings after immersion, and (iii) a silica-rich layer appeared at the interface of the carbonate-containing HA and wollastonite and composite (7/3 with wollastonite/TiO_2_) coatings. The cytocompatibility study of osteoblasts seeded onto the surface indicated that the addition of wollastonite promoted the proliferation of osteoblasts [266].

Haddow *et al*. [267] investigated the effects of dip rate, sintering temperatures, and time on the chemical composition of the titania films, their physical structure and thickness, and adherence to a silica substrate. In order to produce films, the sol-gel method was employed. By this method, films which can be mimiced as closely as possible the natural oxide layer that is found on titanium. CpTi (grade IV) isopropoxide was dissolved in isopropanol to form the starting sol. Due to the ease of hydrolysis of this sol, a chelating agent, diethanolamine, was added. A small amount of water was added to the solution of alkoxide to partially polymerize the Ti species. Thin surface films of titania have been deposited onto glass substrates. These films are to be used as substrates in an *in vitro* model of osseointegration. If titania has been deposited onto glass substrates, the use of low dipping rates prevents cracking in the films, irrespective of the subsequent firing time or temperature. Firing at higher temperature (600 °C) produces predominantly glass films and mimic closely, in chemistry, the natural oxide layer that is formed on Ti implants. Refinement of the dipping set-up, or the use of dilute solutions, may result in the production of thinner films. Thinner films will almost certainly be crack-free after firing, and may result in less organic products being trapped in the film during the firing process. It was mentioned that (i) these data are important to use the titania films to develop an *in vitro* model to study the phenomenon of osseointegration, and (ii) coatings may be deposited onto a wide range of materials; this would be particularly beneficial, enabling the study of osseointegration by TEM and photon-based spectroscopies [267].

Sato *et al*. [268] used the sol-gel processing to coat Ti substrates with HA, TiO_2_, and poly(*DL*-lactic-glycolic acid). Coatings were evaluated by cytocompatibility testing with osteoblast-like cells (or bone-forming cells). The cytocompatibility of the HA composite coatings was compared to that of a traditional plasma-sprayed HA coating. Results showed that (i) osteoblast-like cell adhesion was promoted on the novel HA sol-gel coating compared to the traditional plasma-sprayed HA coating, and (ii) hydrothermal treatment of the sol-gel coating improved osteoblast-like cell adhesion [268]. Wang *et al*. [269] treated an amorphous titania gel layer on the CpTi surface after the Ti surface was treated with a H_2_O_2_/HCl solution at 80 °C. The thickness of the gel layer increased almost linearly with the period of the treatment. It was found that (i) a subsequent heat treatment above 300 °C transformed gradually the amorphous gels to the anatase crystal structure, and the rutile started to appear after heat treatment at 600 °C, and (ii) similar to the sol-gel derived titania gel coatings, titania gel layers exhibited *in vitro* apatite deposition ability after the gel layers exceeded a minimum thickness (0.2 μm) and was subsequently heated in a proper temperature range (400–600 °C) [269].

Titania (5–20 mol%) was mixed with pure HA or HA containing Ag_2_O (10–20 mol%) and was heated at 900 °C for 12 h. The sintered samples were found to contain mainly tricalcium phosphate (β-TCP). Enhanced TCP formation with impurity was observed with TiO_2_-Ag_2_O addition. An *in vitro* solubility study in phosphate buffer at physiological conditions showed the resorbale nature of these materials. It was also mentioned that the gradually functional material structure was formed by spreading a TiO_2_-Ag_2_O mixture on the surface of the HA green compact and heating at 900 °C [270].

### 3.5. Porosity Controlled Surface and Texturing

As a result of coating titanium surfaces, uncontrolled surface porosity is produced. As reviewed in the previous section, not only coated material’s property but also porosity *per se* attributes the favorable osseointegartion. There are several researches and methods proposed to control surface porosity.

Void metal composite (VMC) is a porous metal developed to fix a prosthesis to bone by tissue ingrowth. The material is made by techniques which produce structures with controlled porosity, density, and physical properties. The ability to produce a range of structures creates porosities to study the effects of pore size, shape, and density on bone/metal interface strength. Ti-6Al-4V is the metal of choice for VMC. It was selected for its corrosion resistance, good mechanical properties, low density, and good tolerance by body tissue. Structures with spherical pore size ranging from 275 to 650 μm, and have been fabricated with up to 80% theoretical densities. The optimum structure for attachment strength seems to be a pore size of 450 μm and 50% theoretical density [271]. The control porosity can be achieved by blasting with alumina or titania [272].

The topography of titanium implants is of importance with respect to cellular attachment. Chung *et al*. [273] examined the topographies of three as-received implant systems (Nobelpharma, Swede-Vent, and Screw-Vent), followed by thermal (700 °C for 240 min) and anodic oxidation (70 V in 1M acetic acid solution) of the fixtures. Fixtures were self tapped into freshly sacrificed swine rib bone. It was found that (i) thermal and anodic oxidation, as well as implantation shear stress, had no effect on topography, and (ii) the growth of oxides and implantation shear stress had no affect on topography [272].

Petronis *et al*. [274] developed a model system for studying cell-surface interactions, based on microfabricated cell culture substrates. Porous surfaces consisting of inter-connecting channels with openings of subcellular dimensions are generated on flat, single crystal, silicon substrates. Channel size (width, depth), distribution, and surface coating can be varied independently and used for systematic investigation of how topographical, chemical, and elastic surface properties influence cell or tissue biological responses. Model porous surfaces have been produced by using two different microfabrication methods. Submicron-sized channels with very high depth-to-width aspect ratios (up to 30) have been made by using electron beam lithography and anisotropic reactive ion etching into single-crystal silicon. Another method uses thick-resist photolithography, which can be used to produce channels wider than 1 μm and with depth-to-width aspect ratios below 20 in an epoxy polymer [274]. Xiaoxiong *et al*. [275] created pit with controlled pit density and geometry to exhibit porosity controlled surfaces. The pit initiation process on CpTi in bromide solution was investigated by means of surface analysis. The results showed that the titanium surface film formed by anodic polarization in bromide solution was mainly TiO_2_. Prior to the pit initiation, Br ions were absorbed and accumulated on localized spots of the TiO_2_ film, forming bromine nuclei containing mostly TiBr_4_. The bromine nuclei grew into the critical nuclei when the film was in the critical state of breaking down. The depth of the critical nuclei was equal to or less than 3 nm. The concentration of bromine in the critical nuclei was the critical surface concentration. It was the requisite condition for pit initiation that the concentration of bromine in bromine nuclei reached critical surface concentration. It was mentioned that, in the system of titanium/bromide solution, the critical surface concentration was 25–35 wt% and was independent of the temperature and concentration of the solution [275].

The *in vitro* mineralization of osteoblast-like cells on CpTi with different surface roughness was examined. CpTi discs were polished through 600 grit SiC paper (grooved), polished through 1 μm diamond paste (smooth), or sand-blasted (rough). The discs were cleaned, acid passivated and UV sterilized (254 nm, 300 μW/cm^2^). Osteoblast-like cells were harvested from rat pups and were cultured. The cultures were grown for 6 or 12 days in media supplemented with 5 mM β-glycerophosphate. It was found that (i) *in vitro* mineralization responses were independent of surface roughness, and (ii) Alizarin red staining indicated small zones of mineralization on all surfaces, indicating that surface topography is known to affect osteoblast-like cell activities [272].

Ungersboeck *et al*. [276] investigated five types of limited contact dynamic compression plates of CpTi with different surface treatments and electropolished stainless steel limited contact dynamic compression plates. The surface roughness parameters and chemical surface conditions were determined and checked for probable surface contamination. After an implantation period of 3 months on long sheep bones, the soft tissue adjacent to the plates was evaluated histomorphometrically. The difference in roughness parameters was statistically significant for most surface conditions. It was reported that (i) a correlation was found between the surface roughness of the implants and the thickness of the adjacent soft tissue layer, (ii) the thinnest soft tissue reaction layer with a good adhesion to the implant surface was observed for the titanium anodized plates with coarse surface (20% nitric acid at 60 °C for 30 min), and (iii) smooth implants, in particular the electropolished stainless steel plates, induced statistically significant thicker soft tissue layers [276]. Larsson *et al*. [277] investigated the bone formation around titanium implants with varied surface properties. Machined and electropolished samples with and without thick anodically formed surface oxide were prepared and inserted in the cortical bone of rabbits (1, 3, and 6 weeks). It was found that (i) light microscopic morphology and morphometry showed that all implants were in contact with bone and had a large proportion of bone within the threads at six weeks, (ii) the electro-polished implants, irrespective of anodic oxidation, were surrounded by less bone than the machined implants after one week, (iii) after six weeks the bone volume, as well as the bone-implant contact, were lower for the merely electropolished implants than for the other three groups, and (iv) a high degree of bone contact and bone formation are achieved with titanium implants which are modified with respect to oxide thickness and surface topography; however, the result with the smooth (electropolished) implants indicates that a reduction of surface roughness, in the initial phase, decreases the rate of bone formation in rabbit cortical bone [277].

Thelen *et al*. [278] investigated mechanics issues related to potential use of a recently developed porous titanium material for load-bearing implants. This material may have advantages over solid Ti for enhancing the bone-implant interface strength by promoting bone and soft tissue ingrowth, and for reducing the bone-implant modulus mismatch, which can lead to stress shielding. It was mentioned that (i) simple analytic models provide good estimates of the elastic properties of the porous Ti, and (ii) the moduli can be significantly reduced to decrease the mismatch between solid Ti and bone, achieving the mechanical compatibility proposed by Oshida [279]. The finite element simulations show that bone ingrowth will dramatically reduce stress concentrations around the pores [278]. Takemoto *et al*. [280] prepared porous bioactive titanium implants (porosity of 40%) by a plasma-spray method and subsequent chemical and thermal treatments of immersion in a 5 M aqueous NaOH solution at 60 °C for 24 h, immersion in distilled water at 40 °C for 48 h, and heating to 600 °C for 1 h. It was reported that compression strength and bending strength were 280 MPa (0.2% offset yield strength 85.2 MPa) and 101 MPa, respectively. For *in vivo* analysis, bioactive and nontreated porous titanium cylinders were implanted into 6 mm diameter holes in rabbit femoral condyles. It was found that (i) the percentage of bone-implant contact (affinity index) of the bioactive implants was significantly larger than for the nontreated implants at all post-implantation times (13.5 *versus* 10.5, 16.7 *versus* 12.7, 17.7 *versus* 10.2, 19.1 *versus* 7.8 at 2, 4, 8, and 16 weeks, respectively), and (ii) the percentage of bone area ingrowth showed a significant increase with the bioactive implants, whereas with the nontreated implants it appeared to decrease after four weeks (10.7 *versus* 9.9, 12.3 *versus* 13.1, 15.2 *versus* 9.8, 20.6 *versus* 8.7 at 2, 4, 8, and 16 weeks, respectively), suggesting that porous bioactive titanium has sufficient mechanical properties and biocompatibility for clinical use under load-bearing conditions [280].

### 3.6. FoaMed Metal

The foam-shaped materials exhibit a continuously connected ligamented, reticulated open-cell geometry having a duodecahedronal cell shape. The foam-shaped material is in a density ranging from 1 to 20% theoretical, and a cell density of 10 to 50 cells per linear inch, with material density and cell density independently variable. Foamed materials are presently produced in a wide range of plastics, metals, and composites having either solid or tubular ligaments. The metals include aluminum, nickel, copper, silver, zinc, leads, tin, magnesium, and stainless steel [281]. Unfortunately, although the technology for fabricating foamed metal is available, there is no report found on foamed titanium yet.

## 4. Dental implants for Growing Patients

Considerable research supports the efficiency of rehabilitation of a completely or partially edentulous mandible and maxilla using prosthesis supported by implants [282,283]. However, almost all scientific investigations regarding implants have been performed in adults, when the dynamics of growth and development (statural, skeletal, facial, dentoalveolar) are not an issue. Currently, it is seen that severe hypodontia or even anodontia in children or adolescents, most often associated with congenital syndromes such as ectodermal dysplasia (ED), are treated with implant-supported prostheses for optimum functional and psychosocial development of the pediatric patient. The insertion of dental implants in children or adolescents before the completion of craniofacial growth, however, could cause problems since the maxilla and the mandible are dynamically changing during childhood [284–287]. Therefore, several relevant aspects should be considered before inserting an implant in growing patients.

### 4.1. Special Considerations for Implant Therapy in Children

Implant timing and implant positioning are the important issues to be assessed carefully before the implant therapy in young patients with dynamic growth. The finding of the ideal time for the implant treatment in children has been reported as quite difficult since many different aspects have to be considered while finding the best individual treatment strategy [288]. Additionally, lack of relevant long term clinical implant studies in children and its effects on the development of the maxillofacial structures also could create problems on implant timing in young patients. From the orthodontic view, it is known that the safest time to place implants is the time during the lower portion of the declining adolescent growth curve at or near adulthood [289]. A majority of studies have advocated delaying implant placement until skeletal and dental growth has been completed [290–292] especially when natural teeth are present [292,293]. However, in some cases, especially in children with completely edentulous mandible and maxilla, insertion of an endosseous implant could be necessary before the craniofacial growth is completed. Furthermore, in assessing young patients with implants caution must be exercised in generalizing the results since there are difficulties in prediction of the growth process which varies from individual to individual.

The potential problems associated with placing endosseous implants in growing patients have been addressed by many authors [292,294–299]. The fact that the implants does not follow the normal growth of the maxilla or mandible in the three planes of space and may interfere with the normal growth of the alveolar process are important issues in implant dentistry of growing patients [296,300,301]; and additionally, the end result of an osseointegrated implant placed during growth could be difficult to predict.

There is no capacity for compensatory eruption or physiological movement of the implant fixture in individuals where growth is incomplete since osseointegrated implants lack the compensatory mechanisms of a periodontal ligament and are in direct apposition to bone [298,299]. An osseointegrated implant behaves like an ankylosed tooth and become submerged because of growth associated with the continued eruption of neighboring natural teeth [298]. In the nearly anadontic child, however, these problems can be neglected. Placement of implants in the growing maxilla and/or mandible with only a few missing permanent teeth has been studied, and it has been demonstrated both clinically [292,302] and experimentally [298,299] that osseointegrated endosseous implants adjacent to the natural teeth do not move in vertical, transverse, or sagittal direction and become submerged because of lack of associated growth of the alveolar growth and continued eruption of neighboring natural teeth [297–299,303]. The authors [298,299] stated, however, that it is difficult to directly extrapolate the results from animals to growing children. Additionally, it was also emphasized that the fact that implants placed adjacent to natural teeth in a growing child will become submerged should not necessarily be considered a contraindication to the use of endosseous implants [288]. Kawanami *et al*. [304] states that infraposition occurs even in patients more than 20 years old. The studies have also reported that the changes in adults occur over decades and also result in teeth misalignment. Additionally, it is known that the majority of skeletal growth in females is completed by 15 years, but males grow up to 25 years [292,296].

The bony apposition and resorption patterns could alter the position of implants placed in maxilla and mandible. Maxillary growth occurs as a result of both passive displacement and enlargement. Passive displacement occurs as the maxilla is carried downward and forward by growth and flexion of the cranial base nada complicated system of sutures in the midface [305]. Because of the resorptive aspects of maxillary growth at the nasal floor and the anterior surface of the maxilla, unpredictable dislocations in vertical and anteroposteror direction can occur and even implant losses may be expected. Cronin *et al*. [302] have stated delaying implant placement in the growing maxilla until early adulthood. It is likely that in the absence of teeth, the alveolar bone apposition in a vertical dimension is inhibited and that implant position is affected only by sutural growth in the maxilla. In mandibula, it was noted [306] that mandibular growth pattern is generally characterized by upward and forward curving growth in the condyles, with anteroposterior growth occuring mainly at the posterior mandible [286]. It was stated that the rotational growth resulting particularly in vertical alterations [286,287]. It was also demonstrated that the position of implants placed in the posterior mandible in growing pigs could be altered as a result of bony apposition and resorption patterns, leading to a multidimensional dislocation of the implants [299]. To our knowledge, there exist in the literature no reports on implant insertions in the posterior mandible in pediatric patients. Additionally, one other potential problem of implants in growing tissues is the fact that the implants could jeopardize the germs of the adjacent permanent teeth or alter the path of eruption [299,303].

The fixed implant constructions crossing the midpalatal suture will result in a transversal growth restriction of the maxilla since the transversal growth of the maxilla occurs mostly at the midpalatal suture. In the mandible, the majority of transversal growth at the mandibular symphyseal suture occurs quite early in childhood, usually ceases in the first six months, therefore, the transversal skeletal growth or alveola-dental changes are less dramatic than the maxilla [306]. Kearns *et al*. [307] has reported that no interference with transverse mandibular growth is to be expected when implants are installed in the anterior mandible. Because maxillary transverse growth at the midpalatal suture has been suggested to be adversely affected by rigid prosthetic devices [297], all maxillary prosthetic bar attachments that cross the maxillary midline can be separated in the midline to maintain uninterrupted growth at the midpalatal suture [292,307].

Implants placed in edentulous jaws of growing patients provided the most predictable outcomes in the edentulous anterior mandible, fixtures moved in harmony with sagittal mandibular growth and during the follow-up period no alteration in abutment or prosthesis was required. Several case reports of implant insertions in the anterior mandible of children have been published [7,308–310]. However, the use of implants to replace single teeth in the anterior mandible is not advisable due to the compensatory anteroposterior and the vertical growth in this area. Consequently, implants would remain in an infraocclusal position and would probably be displaced in the anteroposterior direction [306].

A multidisciplinary approach to implant treatment is recommended for children [311,312]. A pediatric dentist, an orthodontist, a prosthodontist, and an oral and maxillofacial surgeon are necessary for the the best individual implant treatment strategy. All specialities have an impact on the process by contributing their specific views and knowledge on rehabilitation [290]. The status of skelatal growth, the individual status of the existing dentition (the degree of hypodontia, the functional status of mastication and phonetics) should be evaluated, esthetic aspects and dental compliance of both the pediatric patient and parent to implant treatment and implant hygiene should be taken into account in determining the optimal individual time period of implant insertion [307].

A risk/benefit assessment must be made for each individual to optimize dental rehabilitation [313]. It should be remembered also that there are significant shortcomings of removable prostheses. Poor retention and instability of prostheses [314], dental hygiene problems, speech difficulties, and dietary limitations, moreover, progressive resorption of basal bone when the edentulous ridge is loaded by prosthesis at an early age should be weighed against the need to change abutments and the possibility that implants will need to be removed at a later date. Furthermore, in young patients’ social development, emotional/psychological well-being [315], extension of related psychosocial stress is important issues. Congenitally missing teeth can create dental and facial disfigurement, which can lead to social withdrawal, especially in the adolescent years [316]. Bergendal *et al*. [308] have stated that functional, esthetic, and psychological rehabilitation should start early in the patient’s life. Högberg *et al*. [317] reported that children with disabilities realize at the age of 9 years their specific conditions when they compare themselves with other children. Nussbaum *et al*. [318] has focused the cases probably resulting in a state of depression. Therefore, the dental team also has to support the child in coping with issues of attractiveness during the formative years of childhood [317] and could apply dental implants while they are still growing [316]; benefits of placing implants in young patients should not be discouraged. Guckes [319] has stated that if implant –supported prostheses were shown to have positive effects on craniofacial growth, social development, self image, and food choice, their use in the anterior mandible might be routinely recommended in younger patients. Dhanrajani and Jiffry [314] have reported that the patient’s skeletal and dental maturity, not chronological age should be the determining factor for the time of implant placement, and the parents should be informed about the possible complications.

### 4.2. Clinical Studies on Use of Endosseous Implants

The literature contains several anecdotal reports of the use of dental implants in growing patients, many with anodontia or oligodontia, often associated with ED, or from trauma [295,296,308,320–324]. The lack of relevant long-term clinical studies has not prevented clinicians from using implant-assisted prostheses in children; dental implants in children were described in dental literature as a successfull adjunt to oral rehabilitation [308,314,320,325,326]; and implant success rates were reported as 87% in preadolescents (ages 7–11), 90% in adolescents (ages 12–17), and 97% in adults (older than 17) [318]. The studies have demonstrated that implants may have high success rates in the edentulous child [307], and implants placed in girls after 15 and in boys after 18 years of age have a better prognosis than in younger children [297,302]. Stable implant conditions were reported after an observation period of 4 to 5.5 years in children with ED [308,320,325,327].

Alcan *et al*. [328] reported on 6-year follow-up of a child with ectodermal dysplasia who was treated with implants surgery very early. In edentulous patients, the 10-year survival rates of such implants were 82% and 94% for the maxilla and mandible, respectively [307]. The mandibular endosseous implant was placed in a 4-year-old patient with hypohidrotic ectodermal dysplasia and oligodontia. It was reported that (i) the congenital anomaly does not appear to retard healing and the osseointegration remains after six years and three months of loading, (ii) mandibular and maxillary skeletal growth and development was normal, (iii), however, because of lack of alveolar growth, in time, patient’s vertical growth pattern changed to low angle. This could be corrected by changing the vertical heights of the abutment and prosthesis. As a result, in ectodermal dysplasias cases with anadontia, early implant placement and fixed prosthesis could be a good multidisciplinary treatment option for poor cooperative child. Balshi *et al*. [329] claimed the advantages of implants (Ti Brånemark implants, and zygomatic implants as well) due to biomechanical and esthetic uniqueness associated with implant-related prosthodontic orofacial rehabilitation for ED patients. A similar clinical opinion can be found from Becktor *et al*. [330], who used the endosseous implants in the oral rehabilitation of adolescent patients with HED and reported it should be considered a viable treatment option. The clinical report of a 18-year-old patient with hypohidrotic ED treated with a maxillary implant overdenture and a mandibular hybrid prosthesis supported by osseointegrated implants have presented significant improvements in oral function and psychosocial activities at the one-year follow-up [331]. Patients with oligodontia may benefit from the use of dental implants in the mandibualr anterior region, with restoration of function and improvement in psychosocial development, without waiting for the completion of growth to initiate treatment [7]. In older ED patients, for whom growth has stabilized, osseointegrated implants can be used as an alternative treatment to support, stabilize, and retain the prosthesis [308,314].

Failures in dental implant treatment can be classified as early or late depending on certain complications. Late failures are usually attributed to peri-implantitis and/or occlusal overload. However, the hypothesis that occlusal overload causes peri-implant bone loss is still being debated [332–335], and scientific evidence for such a relationship has not been fully established [336]. In several studies [337–339], marginal bone defects similar to periodontal lesions found around teeth were created around the peri-implant tissues experimentally, through plaque accumulation promoted by various methods [7]. Although an increasing number of reports have presented the successful regenerative treatment of peri-implantitis defects [7,340–342], histologic evidence of re-osseointegration in humans is lacking. Persson *et al*. [343] demonstrated only a dense connective tissue capsule formation in the peri-implantitis defects next to commercially pure titanium surfacesin a dog study. However, the same authors recently demonstrated substantial re-osseointegration next to a sandblasted/acid-etched surface in another dos study [343,344]. Also, rapid biologic host recovery of the sandblasted/acid-etched surface was shown, with early radiographic signs of loss of osseointegration [316].

Becktor *et al*. [330] pointed out that opinions vary as to whether it is advisable to place endosseous implants in growing patients, since there is a lack of scientific knowledge concerning the fate of these implants and associated prosthetic rehabilitation. Bone volume in young patients may not be sufficient for placing the implants in ideal positions to support the prosthesis. Also unknown is what happens psychologically to these patients when no treatment or various temporary solutions are provided. It was postulated that implants in the alveolus of a young, growing maxilla may become significantly buried in bone and their apical portions exposed as the nasal floor and maxillary sinuses remodel. The effect of remodeling in the presented subject can be seen on the maxillary superimposition. Björk *et al*. [284] showed an average nasal floor remodeling of 4.6 mm in boys aged four to 20 years. The inferior repositioning related to surface remodeling in the present patient was 3.8 mm. Because the continuous lengthening of the maxilla occurred posterior to the implants, the implants moved in harmony with the sagittal displacement and growth of the maxilla. No transverse enlargement could be registered in the tuberosity region of models from age nine to 20 years. By not rigidly connecting the right and left implants, interference at the midline growth suture could possibly be avoided [307].

Bergendal *et al*. [345] reported that dental implant placement has been a rarely used treatment modality in Swedish children less than 16 years old in the last 20 years. The failure rate in children treated because of tooth agenesis was only slightly higher than that reported for adult individuals, where in young children with ED and anadontia in the mandible, implants seemed to present special challenges, and the failure rate was very high. The small jaw size and preoperative conditions were thought to be the main risk factors.

The fact that the imp does not follow the normal growth of the maxilla or mandible in the three planes of space and behaves like an ankylosed tooth has been demonstrated in some clinical studies [296,300,301]. Additionally, Rossi and Andreasen [346], analyzed the unfavorable clinical and radiographic findings of a single-tooth replacement in a 10 year child and reported 9 mm discrepancy between the implant collar and the CEJ of the adjacent teeth in 25 years of age; additionally they have stated that there was no significant bone loss at the implant site during a 10 year observation.

### 4.3. Alternative Treatments

In childhood, a removable partial denture (RPD) or complete overdenture is often the treatment of choice because of the need to easily modify the intraoral prosthesis during rapid growth periods. These treatment options afford the patient and his or her family an easy, affordable, and reversible method of oral rehabilitation. Cooperation of the patient, as well as the support of the family, are necessary of removable prostheses are to be successful in young patients. The functional, esthetic, and psychologic benefits of successful prosthetic restoration for these children should be weighed against the need to change abutments and the possibility that implants will need to be removed at a later date. Rockman *et al*. [347] reported a technique using magnets to enhance the retention of maxillary and mandibular prostheses in a 9-year-old boy, and suggested that the case report introduces an alternative prosthetic design for children.

It was demonstrated that anadontia has many adverse effects on the psychological and physiological conditions of patients during childhood. Therefore, complete dentures must be applied. As a rule, the younger the child, the easier will be the adaptation to the denture. However, treatment is completely dependent on patient-parent cooperation [348]. Based on oral examination showing total anadontia in both maxillary and mandibular arches, a 5 year old male patient who had ED with anadontia was treated with upper and lower complete dentures. It was reported that retention and stabilization of the dentures were clinically acceptable. Prosthodontic treatment in patients with ectodermal dysplasia is difficult to manage because of the oral deficiencies typical in this disorder and because afflicted individuals are quite young when they are evaluated for treatment. Furthermore, pediatric patients, with oligodontia or anadontia, using prostheses to restore form and function can be a challenge.

In conclusion, the use of endosseous implants is a viable option for dental rehabilitation of children with anadontia or oligodontia. However, the published reports about implant application in young patients are as yet very limited. Well controlled, randomized, prospective longitudinal trials that include a sufficient number of patients are needed; and for a successful outcome, a multidisciplinary approach for oral and maxillofacial rehabilitation of these patients is strongly recommended.

## 5. Future Perspectives

Research and development in science and technology should not be discrete. If we evaluate correctly and examine carefully what have been done in the past, we will be able to foresee what would be available for us in future. Titanium materials science is a typical interdisciplinary science and engineering. In order for engineered titanium materials to serve as titanium biomaterials, we have been discussing and reviewing numerous articles to prove that appropriate surface modifications and characterizations should be properly preformed and reflected to appropriate fabrication technologies and methods.

In the past, when tissues became diseased or damaged, a physician had little recourse but to remove the offending part, with obvious limitations. Removal of joints, vertebrae, teeth, or organs led to only a marginally improved quality of life. However, human survivability seldom exceeded the progressive decrease in quality of tissues, so the need for replacement parts was small. During the last century the situation changed greatly. The discovery of antiseptics, penicillin and other antibiotics, chemical treatment of water supplies, improved hygiene, and vaccination all contributed to a major increase in human survivability in developed countries. Life expectancy is now in the range of 80+ years. In the past, it was the major practice to remove the diseased tissues, whereas at the present, either transplants (using autografts, heterografts, or homografts) or implants (using biological fixation, bioactive fixation, or cement fixation) are commonly utilized, and in the future, regeneration of tissues, based on engineered tissues and regenerative bioactive materials will become a major clinical impact. Regeneration of tissues implies restoration of structure, restoration of function, restoration of metabolic and biochemical behavior, and restoration of biomechanical performance. Our challenge for the future is to extend these findings to studies in compromised bones with osteopenia and osteoporosis, to apply the findings to larger animals, and especially humans, with aging bones, and to use the findings to design the 3-D architectures required for engineering of tissues [349].

Based on what we have seen so far, we can see further research and development in materials, technology, engineering and clinical applications to provide better health services coming years, particularly characterized by an ever-aging society. In this section, among many future perspectives associated with medical and dental, as well as industry, Ti biomaterials development and related technologies will be revealed.

### 5.1. Titanium Industry and New Materials R&D

The titanium industry is rebounding with projections that the worldwide mill product shipments by 2008 will be about 56,000 tones per year – up significantly from the 43,000 tones per year of 2002/2003. This is a result of the increase in commercial airplane production along with increased sales to the military, industrial, and consumer markets. As always, cost remains the major barrier to ore titanium use, especially in the industrial and consumer markets [350]. Numerous attempts have been undertaken in the last 60 years to reduce the cost of producing titanium [351–353]. With the advent of high-quality, lower-cost titanium powders, the emphasis in titanium powder metallurgy P/M technology has centered on production of near-net shapes with acceptable levels of mechanical properties. The pre-alloyed, blended elemental, and metal injection molding (MIM) approaches are all looking attractive, and should post significant growth in the next few years [354].

Among many developed titanium materials, these are just few to list of newly developed titanium materials; V-free Ti alloys [355,356], Ti-Pd-Co alloy [357], Ti-V-Fe-Al alloy [358], Ti-Cr alloy [359], Ti-Cu-Ni-Sn-M (M: Nb, Ta, Mo) alloy [360], Ti-Cu-Pd [361], Ti-Cu-Si [362], Ti-Zr [363], Ti-Hf [364,365].

Amorphous materials are non-crystalline solids. For the last three decades, amorphous alloys have attracted great interest because of the results from their new alloy compositions and new atomic configurations [366]. TiAl amorphous alloy provides high strength, linear elastic behavior, and the infinite fatigue life necessary for high device reliability. This alloy was originally developed for material for the digital micromirror device (DMD) chip, and actually it is a TiAl_3_ phase [367]. Bulk amorphous Ti-based alloys were found to be formed in the diameter range up to 5 mm for the Ti-Ni-Cu-Sn and Ti-Ni-Cu-Si-B systems, which possess a high glass-forming ability [366,368]. There are newly reported Ti45-Ni20-Cu25-Sn5-Zr5 [369] and Ti50-Cu20-Ni24-Si4-B2 [370] that can be amorphatized, too.

### 5.2. Gradient Functional Material System

Material is composed of multilayer, with each layer having unique characteristics, yet adjacent layers having some similarity is called gradient functional material (GFM). Although such functions can include various properties, it is limited to mechanical, physical, or thermal properties since other properties, such as chemical or electrochemical, are more likely important to the surface layer, and not related to bulky or semi-bulky behavior. For example, if hydroxyapatite is needed to spray-coat onto CpTi, this GFM concept can be applied. Instead of applying HA powder directly onto the CpTi surface, a multilayer of the following sequence: HA/HA + Al_2_O_3_/Al_2_O_3_ + TiO_2_/TiO_2_/CpTi can be prepared to enhance the bonding strength. From the HA side to CpTi side, the mechanical properties (particularly, modulus of elasticity) and thermal properties (such as linear coefficient of thermal expansion) are gradually changing, so that when this HA-coated CpTi is subjected to stressing, interfacial stress between each constituent layer can be minimized, resulting in that the degree of discreteness in the stress field can also be minimized.

A novel technology for forming a gradient functional titanium-oxide film on a titanium alloy (Ti-6Al-4V) was developed by the reactive DC sputtering vapor deposition method [371]. The method was developed for fabricating denture bases and implants, and the oxygen concentration was changed continuously during sputtering to provide a gradient in the film composition, by which adhesivity to the alloy, surface hardness, and biocompatibility was improved. Denture bases produced by superplastic forming (SPF) are cleaned with an organic solvent, the oxygen concentration is changed continuously during sputtering, and pure titanium is vapor-deposited by the reactive DC sputtering. In the initial stage, intermetallic bonding is achieved by oxygen-free vapor deposition, so the adhesion is excellent and there is no fear of exfoliation. But farther away from the metal surface, the oxygen concentration is raised gradually to form a gradient film. At the surface some titanium oxides are formed. Titanium oxide features excellent biocompatibility, and since it is a hard material, resists damage. The overall film thickness in the experimental was 3 μm, and the Vickers hardness of the surface was 1,500 (200–300 for pure titanium) [371].

Bogdanski *et al*. [372] fabricated the functionally graded material, which was prepared through powder metallurgical processing with thoroughly mixed powders of the elements. Ten mixtures were prepared ranging from Ni:Ti of 90:10 (by atomic %) through 80:20, to Ni:Ti of 10:90, and pure Ti (0:100). The compaction was done by hot isostatic pressing (HIP) at 1,050 °C and 195 MPa for 5 h. It was reported that, using cells (comprised of osteoblast-like osteosarcoma cells, primary human osteoblasts, and murine fibroblasts), good biocompatibility of Ni-Ti alloys has shown up to about Ni 50% [372].

### 5.3. Coating

Surface modifications have been applied to metallic biomaterials in order to improve their wear properties, corrosion resistance, and biocompatibility. Methods of applying calcium phosphate-based materials are being actively investigated with the aim of enhancing osteoinduction on titanium materials [373,374]. This work is necessary because a plasma sprayed calcium phosphate coating has disadvantages, such as the need for a critical thickness to ensure complete coverage of the implant surface [375]. Another approach to enhancing osteoinduction is to promote the formation of hydroxyapatite on titanium in the human body. Calcium ion implantation [376] and a CaTiO_3_ coating [377] for titanium materials have been examined, and the improvement of biocompatibility with bone was confirmed.

The development of a post-operative infection following the implantation, such as a Ti-6Al-4V alloy total joint prosthesis, is a severe complication in many orthopedic surgeries. Preventing these bacterial infections could theoretically be accomplished by administering therapeutic doses of antibiotics as close to the implant site as possible. Mixing antibiotics with PMMA (polymethylmetaacrylate) bone cements has been shown to provide adequate local antibiotic concentrations for extended periods of time [378–380]. Because metallic materials dominate orthopedic bioprosthetic devices, there exists a definite need for developing methods to attach antibiotics to metallic surfaces. Since the naturally forming passive surface oxide layer of Ti-6Al-4V is thought to be responsible for the excellent biocompatibility and corrosion resistance of this alloy, this oxide layer would be a natural choice for facilitating antibiotic attachment and retainment. By carefully controlling the surface chemistry of the oxide and utilizing the pH dependence of surface charge characteristics of the oxide, the attachment of charged antibiotics may be facilitated at suitable pH values. Such a concept has already been successfully tested with macroporous oxides (1–10 μm pores) formed in sulfuric acid solutions [381]. Dunn *et al*. [378] also studied the microporous (about 1.5 μm) anodized oxides formed on Ti-6Al-4V alloy to facilitate the attachment and sustained release of antibiotics for longer times. The degree of entamicin sulfate attachment and retainment to microporous oxide layers created on the surface of Ti-6Al-4V materials was determined to be a function of the oxide morphology and surface chemistry. Sulfuric (5–10%) anodized samples were observed to retain the electrostatically attached antibiotic for a period of 13 days when washed in saline at a pH of 7.4. It was found that a longer retention of gentamicin by potentiostatically anodized surfaces in phosphoric acid may be attributed to the lower isoelectric point and more negative zeta potential of these surfaces [378]. Similar studies were conducted by Kato *et al*. [382,383] to evaluate the applicability of the titanium material as a carrier or a substratum. Spongy titanium adsorbed bone morphogenetic protein (BMP) was implanted in muscle pouches in the thighs of mice. It was found that the quantity of new bone induced was somewhat less than that of the control.

The adsorption of bovine serum albumin (BSA) on titanium powder has been studied as a function of protein concentration and pH, and in the presence of calcium and phosphate ions. Isotherm data have shown that the adsorption process does not follow the Langmuir model (inflection points). For the pH dependence of adsorption, it was found that (i) the amount adsorbed increased with decreasing pH, indicating that hydration effects are important, and (ii) adsorption increases and decreases in the presence of calcium and phosphate ions, indicating that electrostatic effects are important. The time dependence, isotherm, and desorption data provide indirect evidence of possible conformational changes in the BSA molecule [384]. Hence, protein adsorption is a dynamic event with proteins adsorbing and desorbing as a function of time. McAlarney *et al*. [385] investigated the role of complement C3 in the competitive adsorption of proteins from diluted human plasma (the Vroman effect) onto TiO_2_ surfaces. Ti oxide surfaces were made: (1) four anatase surfaces (70 nm, 140 nm, 70 nm aged and solid anatase), (2) three rutile surfaces (70 nm, 140 nm, and solid rutile), and (3) one electropolished Ti. It was found that (i) in both rutile and anatase surfaces, there was an increase in adsorption with increasing oxide film thickness and/or crystallinity, and (ii) anatase surfaces had greater C3 concentration than the equivalent rutile surfaces [385].

Titanium dental implants are widely used with success, but their rejection is not rare. One of the causes for implant failure may be due to biofilms created by interactions between the implant material and the surrounding tissues and fluids. The study described the selective adsorption of a specific salivary protein to Ti-oxide and the mechanism of adsorption. Klinger *et al*. [386] treated enamel powder, CpTi powder, as well as Ti powder by Ca, Mg, or K, which were suspended *in vitro* in human clarified whole saliva, or in various concentrations of purified salivary constituents, at pH 3.0 and 7.0. The powders were then suspended in EDTA solution in order to release proteins that may have adsorbed to their surfaces. It was found that (i) Ti powders adsorbed considerably less salivary proteins as compared with the enamel powder, (ii) human salivary albumin was identified by Western-immunoblot as the main protein that adsorbed to Ca-treated Ti powder, (iii) the Ca effect was not evident at pH 3.0 due to a neutral-basic shift of the protein at a pH level lower than its isoelectric point, and (iv) the *in vivo* investigation of salivary proteins adsorbing to Ti parts confirmed these results. Based on these findings, it was concluded that albumin was shown to be the main salivary protein adsorbing to Ti via a selective calcium and pH-dependent mechanism, and these findings are important for the understanding of Ti biocompatibility properties, as well as patterns of bacterial dental plaque accumulation on Ti implants, and the consequent implant success [386].

Hayakawa *et al*. [387] investigated to attach fibronectin directly to a titanium surface treated with tresyl chloride (2,2,2-trifluoroethanesulfonyl chloride) for the development of a strong connection of a dental implant to subepithelial connective tissues and/or peri-implant epithelia. Basic terminal OH groups of mirror polished titanium were allowed to react with tresyl chloride at 37 °C for 2 days. After the reaction of fibronectin with titanium, the X-ray photoelectron spectroscopy revealed the remarkable effect of the activation of terminal OH groups with the tresyl chloride treatment. It was mentioned that fibronectin, a well-known cell-adhesive protein, could easily be attached to the titanium surface by use of the tresyl chloride activation technique [387]. Studies in developmental and cell biology have established the fact that responses of cells are influenced to a large degree by morphology and composition of the extracellular matrix. In order to use this basic principle for improving the biological acceptance of implants by modifying the surfaces with components of the extracellular matrix (ECM), Bierbaum *et al*. [388,389] modified titanium surfaces with the collagen types I and III in combination with fibronectin. It was reported that (i) increasing the collagen type III amount resulted in a decrease of fibril diameter, while no significant changes in adsorption could be detected, (ii) the amount of fibronectin bound to the heterotypic fibrils depended on fibrillogenesis parameters, such as ionic strength or concentration of phosphate, and varied with the percentage of integrated type III collagen, and (iii) the initial adhesion mechanism of the cells depended on the substrate (titanium, collagen, fibronectin) [388,389].

Collagen, as a major constituent of human connective tissues, has been regarded as one of the most important biomaterials. Kim *et al*. [390] investigated the fibrillar self-assembly of collagen by incubating acid-dissolved collagen in an ionic-buffered medium at 37 °C. It was reported that (i) the degree of assembly was varied with the incubation time and monitored by the turbidity change, (ii) the partially assembled collagen contained fibrils with varying diameters, as well as nonfibrillar aggregates, while the fully assembled collagen showed the complete formation of fibrils with uniform diameters of approximately 100–200 nm with periodic stain patterns within the fibrils, which are typical of native collagen fibers, and (iii) without the assembly, the collagen layer on Ti adversely affected the cell attachment and proliferation [390].

A unique surface treatment on Ti was developed by Wang *et al*. [391]. Titanium screws and titanium flat sheets were implanted into the epithelial mantle pearl sacs of a fresh water bivalve by replacing the pearls. After 45 days of cultivation, the implant surfaces were deposited with a nacre coating with iridescent luster. The coating could conform, to some extent, to the thread topography of the screw implant, and was about 200–600 μm in thickness. It was found that (i) the coating was composed of a laminated nacreous layer and a transitional non-laminated layer that consisted mainly of vaterite and calcite polymorphs of calcium carbonate, and (ii) the transitional layer was around 2–10 μm thick in the convex and flat region of the implant surface, and could form close contact with titanium surface while the transitional layer was much thicker in the steep concave regions, and could not form close contact with the titanium surface. It was hence concluded that it was possible to fabricate a biologically active and degradable, and mechanically tough and strong nacre coating on titanium dental implants [391].

Frosch *et al*. [392] evaluated the partial surface replacement of a knee with stem cell-coated titanium implants for a successful treatment of large osteochondral defects. Mesenchymal stem cells (MSCs) were isolated from bone marrow aspirates of adult sheep. Round titanium implants were seeded with autologous MSC and inserted into an osteochondral defect in the medial femoral condyle. As controls, defects received either an uncoated implant or were left untreated. Nine animals with 18 defects were sacrificed after six months. It was reported that (i) the quality of regenerated cartilage was assessed by *in situ* hybridization of collagen type II and immunohistochemistry of collagen types I and II, (ii) in 50% of the cases, defects treated with MSC-coated implants showed a complete regeneration of the subchondral bone layer, (iii) a total of 50% of MSC-coated and uncoated implants failed to osseointegrate, and formation of fibrocartilage was observed, (iv) untreated defects, as well as defects treated with uncoated implants, demonstrated incomplete healing of subchondral bone and formation of fibrous cartilage. It was, therefore, concluded that in a significant number of cases, a partial joint resurfacing of the knee with stem cell-coated titanium implants occurs, and a slow bone and cartilage regeneration and an incomplete healing in half of the MSC-coated implants are limitations of the method [392]. The osseointegration of four different kinds of bioactive ceramic-coated Ti screws were compared with uncoated Ti screws by biomechanical and histomorphometric analysis by Lee *et al*. [152]. Calcium pyrophosphate, 1:3 patite-wollastonite glass ceramic, 1:1 apatite-wollastonite glass ceramic, and bioactive CaO-SiO_2_-B_2_O_3_ glass ceramic coatings were prepared and coated by the dipping method. Coated and uncoated titanium screws were inserted into the tibia of 18 adult mongrel male dogs for 2, 4, and 8 weeks. It was reported that (i) at 2 and 4 weeks after implantation, the extraction torque of ceramic-coated screws was significantly higher than that of uncoated screws, (ii) at 8 weeks, the extraction torques of calcium pyrophosphate coated and both apatite-wollastonite glass ceramics-coated screws were significantly higher than those of CaO-SiO_2_-B_2_O_3_ glass-coated and uncoated screws, and (iii) the fixation strength was increased by the presence of osteoconductive coating materials, such as calcium pyrophosphate, and apatite-wollastonite glass ceramic, which enabled the achievement of higher fixation strength even as early as 2–8 weeks after the insertion [152].

Bigi *et al*. [393] performed a fast biomimetic deposition of hydroxyapatite (HA) coatings on Ti-6Al-4V substrates using a slightly supersaturated Ca/P solution, with an ionic composition simpler than that of simulated body fluid (SBF) to fabricate nanocrystalline HA. It was found that (i) soaking in supersaturated Ca/P solution results in the deposition of a uniform coating in a few hours, whereas SBF, or even 1.5 × SBF, requires 14 days to deposit a homogeneous coating on the same substrates, (ii) the coating consists of HA globular aggregates, which exhibit a finer lamellar structure than those deposited from SBF, and (iii) the extent of deposition increases on increasing the immersion time [393].

### 5.4. Fluoride Treatment

It is known in the literature that fluoride ions have osteopromoting capacity leading to increased calcification of the bone. Titanium fluoride is reported to form a stable layer on enamel surfaces consisting of titanium which share the oxygen atoms of phosphate on the surface of hydroxyapatite. Ellingsen *et al*. [394] investigated as to whether a similar, or rather reverse, reaction would take place on fluoride pre-treated titanium after implantation in bone. Threaded TiO_2_-blasted titanium implants were pre-conditioned with fluoride. The implants were operated into the tibia of Chinchilla rabbits and let to heal for two months before sacrificing the animals. The strength of the bonding between the implants and bones was tested by removing the implants from the bones by the use of an electronic removal torque gauge. It was reported that (i) the fluoride conditioned titanium implants had a significantly increased retention in bone (69.5 N-cm) compared to non-treated blasted implants (56.0 N-cm) and smooth surface implants (17.2 N-cm), and (ii) the histological evaluation revealed that new bones formed on the surface of the test implants, as well as in the marrow or cancellous regions, which was not observed in the control groups, suggesting that fluoride conditioning of titanium has an osteopromoting effect after implantation [394]. Furthermore, push-out tests of fluoridated and control Ti implants placed in rabbits for up to 8 weeks were conducted [395]. It was found that the fluoridated implants sustained greater push-out forces than the controls, and substantial bone adhesion was observed in fluoridated implants, whereas the controls always failed at the interface between the bone and foreign materials. In other rabbit test conducted by Ellingsen *et al*. [396], it was reported that the fluoridated, blasted implants showed a significantly higher removal torque than the blasted test implant, again indicative of a bioactive reaction of fluoridated Ti implants.

### 5.5. Laser Applications

As mentioned before, laser technology and laser application have advanced remarkably. Lasers can be made to heat, melt, or vaporize materials, depending on laser power density [397,398]. Materials absorb power more readily from Nd:Yag laser beams (λ = 1.06 μm) than they do from CO_2_ laser beams (λ = 10.6μm). By heating materials, materials can be annealed, or solid state phase-transformation hardened. Using melting, materials can be alloyed, cladded, grain refined, amorphatized, and welded. Using vaporization, materials can be thin film deposited, cleaned, textured, and etched. Using shocking, materials can be shock hardened/peened [399]. Recent advances in the Nd:Yag and CO_2_ lasers have made possible a wide range of applications and emerging technologies in the computer, microelectronics, and materials fields. In the realm of materials processing, surface treatments and surface processing, surface treatments and surface modification for metals and semiconductors are of particular interest. With appropriate manipulation of the processing conditions (e.g., laser power density or interaction time), a single laser can be used to perform several processes [400,401].

Laser alloying is a material-processing method that utilizes the high power density available from focused laser sources to melt metal coatings and a portion of the underlying substrate [402]. Since the melting occurs in a very short time and only at the surface, the bulk of the material remains cool, thus serving as an infinite heat sink. Large temperature gradients exist across the boundary between the melted surface region and the underlying solid substrate. This results in rapid self-quenching (10^11^ ks^−1^) and resolidification (velocities of 20 m/s). What makes laser surface alloying both attractive and interesting is the wide variety of chemical and microstructural states that can be retained because of the repaid quench from the liquid phase. The types of observed microstructures include extended solid solutions, metastable crystalline phases and metallic glasses as an amorphous metal [402,403]. Alloy production with a wide variety of elements, as well as a wide range of compositional content, can also be accomplished by a mechanical alloying or powder metallurgy, both of which do not involve liquids (in other words, in solid state fabrication).

Direct laser forming (DLF) is a rapid prototyping technique that enables prompt modeling of metal parts with high bulk density on the base of individual three-dimensional data, including computer tomography models of anatomical structures. Hollander *et al*. [404] investigated DLFed Ti-6Al-4V for its applicability as hard tissue biomaterial. It was reported that rotating bending tests revealed that the fatigue profile of post-DLF annealed Ti-6Al-4 V was comparable to cast/hot isostatic pressed alloy. In an *in vitro* investigation, human osteoblasts were cultured on non-porous and porous blasted DLFed Ti-6Al-4V specimens to study morphology, vitality, proliferation and differentiation of the cells. It was reported that (i) the cells spread and proliferated on DLFed Ti-6Al-4V over a culture time of 14 days, (ii) on porous specimens, osteoblasts grew along the rims of the pores and formed circle-shaped structures, as visualized by live/dead staining, as well as scanning electron microscopy, and (iii) overall, the DLFed Ti-6Al-4V approach proved to be efficient, and could be further advanced in the field of hard tissue biomaterials [404].

Recently, the femtosecond-laser-based tooth preparation technique has been developed [405,406]. Any one of the existing laser technologies using a CO_2_ laser, Er:Yag laser, Ho:Yag laser, excimer laser, frequency-doubled Alexandrite laser, superpulsed CO_2_ laser, or picosecond Nd:Yag laser induce severe thermal adverse effects or do not supply sufficient ablation rates for completion of the mechanical drill [407]. Using the femtosecond laser technology for micromachining was successfully developed, for example in machining tools for repairing photolithographic masks or fuel injector nozzles [408].

### 5.6. Near-Net Shape (NNS) Forming

In order to achieve the better condition for fit, for example superstructure for implants or denture base for prostheses, the accuracy of the final products are very crucial and need to be improved. Among various manufacturing technologies, near-net shape forming and nanotechnology are most promising and supportive for these specific aims. New classes of fabrication processes, such as direct-write (DW) technique [409] and solid freedom fabrication (SFF) [410], have established the capability to produce 3-dimensional parts faster, cheaper, and with added functionality. The choice of starting materials and the specific processing technique will produce unique microstructures that impact the final performance, especially of macroscopic structural and electronic parts. Furthermore, the ability to do point-wise deposition of one or more materials provides the opportunity for fabricating structures with novel microstructural and macrostructural features, such as micro-engineered porosity, graded interfaces, and complex multi-material constructions. Near-net shape (NNS) processing offers cost reduction by minimizing machining, reducing part count, and avoiding part distortion from welding. NSS technologies such as flow-forming (FF), superplastic forming (SPF), casting, forging, powder metallurgy (P/M) methods, three-dimensional laser deposition, and plasma arc deposition (PAD) have been explored for potential use in tubular geometries and other shapes of varying complexity. It appears that the reduction in cost of a given titanium product will be maximized by achieving improvements in all of the manufacturing steps, from extraction to finishin [411].

### 5.7. Tissue Engineering and Scaffold Structure and Materials

Tissue engineering can perhaps be best defined as the use of a combination of cells, engineering materials, and suitable biochemical factors to improve or replace biological functions [412]. The advanced medicine indicates an interdisciplinary field that applies the principles of engineering and life sciences toward the development of biological substitutes that restore, maintain, or improve tissue function for understanding the principles of tissue growth, and applying this to produce functional replacement tissue for clinical use [412]. The term “regenerative medicine” is often used synonymously with “tissue engineering”, although those involved in regenerative medicine place more emphasis on the use of stem cells to produce tissues [412]. Tissue engineering *in vitro* and *in vivo* involves the interaction of cells with a material surface. The nature of the surface can directly influence cellular response, ultimately affecting the rate and quality of new tissue formation. Initial events at the surface include the orientated adsorption of molecules from the surrounding fluid, creating a conditioned interface to which the cell responds. The gross morphology, as well as the micro-topography and chemistry of the surface, determine which molecules can adsorb and how cells will attach and align themselves. The local attachments made of the cells with their substrate determine cell shape, which, when transduced via the cytoskeleton to the nucleus, result in expression of specific phenotypes. Osteoblasts and chondrocytes are sensitive to subtle differences in surface roughness and surface chemistry. Boyan *et al*. [413] investigated the chondrocyte response to TiO_2_ of differing crystallinities, and showed that cells can discriminate between surfaces at this level as well. Cellular response also depends on the local environmental and state of maturation of the responding cells. It was mentioned that optimizing surface structure for site-specific tissue engineering is one option; modifying surfaces with biological means is another biological engineering [413].

One major determination of the suitability of various engineering materials for use in biological settings is the relative strength of adhesion obtained between those materials and their contacting viable phases [414]. Maximal adhesive strength and immobility are desired for orthopedic and dental implants. For example, while minimal bio-adhesion is critical to preventing unwanted thrombus formation in cardiovascular devices, plaque buildup on dental prostheses, and bacterial fouling [414]. Attention should be directed to adhesive phenomena in the oral environment, examining new surface conditioning methods for the prevention of micro-organism deposits, as well as the promotion of excellent tissue bonding to implanted prosthetic devices. Other bio-adhesive phenomena considered included those important to the safe and effective function of new cardiovascular devices [414].

Scaffold material has a two-fold function: artificial extracellular matrices (ECM) and as a spacer keeping a certain open space [415]. Furthermore, scaffold material has to be dissolved completely into the living body after auto-cell is regenerated with artificial extracellular matrices [415]. There are several important biodegradable and/or biofunctional scaffold architectures, structures and materials. They include blended-polymer scaffolds, collagen-based scaffolds, and composite scaffolds of polyhydroxybutyrate-polyhydroxyvalerate with bioactive wollastonite (CaSiO_3_) [416]. Using an ink-injection technique [417], a thin film (with thickness of about 0.1mm) of calcium phosphate and binding agent is injected onto the substrate to build 3-D bony-like structures [418]. Lee *et al*. [419] employed three-dimensional printing (3DP) technology to fabricate porous scaffolds by inkjet printing liquid binder droplets. Direct 3DP, where the final scaffold materials are utilized during the actual 3DP process, imposes several limitations on the final scaffold structure. An indirect 3DP protocol was developed, where molds are printed and the final materials are cast into the mold cavity to overcome the limitations of the direct technique. Results of SEM observations revealed highly open, well interconnected, uniform pore architecture (about 100–150 μm) [419]. Scaffold materials for bone tissue engineering often are supplemented with bone morphogenetic proteins (BMPs). Walboomers *et al*. [420] investigated a bovine extracellular matrix product containing native BMPs. Hollow cylindrical implants were made from titanium fiber mesh, and were implanted subcutaneously into the back of Wistar rats. It was reported that (i) after eight weeks, in two out of six loaded specimens, newly-formed bone and bone marrow-like tissues could be observed, and (ii) after 12 weeks, this had increased to five out of six loaded samples. It was, therefore, concluded that the bovine extracellular matrix product loaded in a titanium fiber mesh tube showed bone-inducing properties [420].

Electrospinning [421] has recently emerged as a leading technique for generating biomimetic scaffolds made of synthetic and natural polymers for tissue engineering applications. Li *et al*. [422] compared collagen, gelatin (denatured collagen), solubilized alpha-elastin, and recombinant human tropoelastin as biopolymeric materials for fabricating tissue engineered scaffolds by electrospinning. It was reported that (i) the average diameter of gelatin and collagen fibers could be scaled down to 200–500 nm without any beads, while the alpha-elastin and tropoelastin fibers were several microns in width, and (ii) cell culture studies confirmed that the electrospun engineered protein scaffolds support attachment and growth of human embryonic palatal mesenchymal cells [422]. For fabricating meshes of collagen and/or elastin by means of electrospinning from aqueous solutions, Buttafoco *et al*. [423] added polyethylene oxide and NaCl to spin continuous and homogeneous fibers. It was reported that (i) upon crosslinking, polyethylene oxide and NaCl were fully leached out, and (ii) smooth muscle cells grew as a confluent layer on top of the crosslinked meshes after 14 days of culture [423].

Surface properties of scaffolds play an important role in cell adhesion and growth. Biodegradable poly(α-hydroxy acids) have been widely used as scaffolding materials for tissue engineering; however, the lack of functional groups is a limitation. Liu *et al*. [424] mentioned in their studies that gelatin was successfully immobilized onto the surface of poly(α-hydroxy acids) films and porous scaffolds by an entrapment process. It was found that (i) the amount of entrapped gelatin increased with the ratio of dioxane/water in the solvent mixture used, (ii) chemical crosslinking after physical entrapment considerably increased the amount of retained gelatin on the surface of poly(α-hydroxy acids), (iii) osteoblasts were cultured on these films and scaffolds, (iv) cell numbers on the surface-modified films and scaffolds were significantly higher than those on controls 4 h and 1 day after cell seeding, (v) the osteoblasts showed higher proliferation on surface-modified scaffolds than on the control during 4 weeks of *in vitro* cultivation, and (vi) more collagen fibers and other cell secretions were deposited on the surface-modified scaffolds than on the control scaffolds [424].

There are still unique scaffold systems developed, such as the collagen-carbon nanotubes composite matrices [425], chitosan-based hyaluronan hybrid polymer fibers system [426], bioactive porous CaSiO_3_ scaffold structure [427], or a three-dimensional porous scaffold composed of biodegradable polyesters [428].

### 5.8. Application of Nanotechnology to Surface Modification

Several studies have suggested that materials with nanopatterned surfaces produced from various chemistries, such as metals, polymers, composites and ceramics, exhibit better osseointegration when compared to conventional materials [429–432]. Nano-patterned surfaces provide a higher effective surface area and nanocavities when compared to the conventional microrough surfaces. These properties are crucial for the initial protein adsorption that is very important in regulating the cellular interactions on the implant surface. Taylor [433] pointed out that nanotechnology offers the key to faster and remote diagnostic techniques – including new high throughput diagnostics, multi-parameter, tunable diagnostic techniques, and biochips for a variety of assays. It also enables the development of tissue-engineered medical products and artificial organs, such as heart valves, veins and arteries, liver, and skin. These can be grown from the individual’s own tissues as stem cells on a 3-D scaffold, 3-D tissue engineering extracellular matrix, or the expansion of other cell types on a suitable substrate. The applications which seem likely to be most immediately in place are external tissue grafts; dental and bone replacements; protein and gene analysis; internal tissue implants; and nanotechnology applications within *in vivo* testing devices and various other medical devices. Nanotechnology is applied in a variety of ways across this wide range of products. Artificial organs will demand nanoengineering to affect the chemical functionality presented at a membrane or artificial surface, and thus avoid rejection by the host. There has been much speculation and publicity about more futuristic developments such as nanorobot therapeutics, but these do not seem likely within our time horizon [433].

There are studies done on nanotubes. For example, Frosch *et al*. [434] investigated the effect of different diameters of cylindrical titanium channels on human osteoblasts. Titanium samples with continuous drill channels with various diameters (300, 400, 500, 600, and 1,000 microns) were put into osteoblast cell cultures that were isolated from 12 adult human trauma patients. It was reported that (i) within 20 days, cells grew an average of 838 μm into the drill channels with a diameter of 600 μm, and were significantly faster than in all other channels, (ii) cells produced significantly more osteocalcin messenger RNA (mRNA) in 600 μm channels than they did in 1,000 μm channels, and demonstrated the highest osteogenic differentiation, (iii) the channel diameter did not influence collagen type I production, and (iv) the highest cell density was found in 300 μm channels, suggesting that the diameter of cylindrical titanium channels has a significant effect on migration, gene expression, and mineralization of human osteoblasts [434]. Macak *et al*. [435] reported on the fabrication of self-organized porous oxide-nanotube layers on the biomedical titanium alloys Ti-6Al-7Nb and Ti-6Al-4V by an anodizing treatment in 1M (NH_4_)_2_SO_4_ electrolytes containing 0.5 wt % of NH_4_F. It was shown that (i) under specific anodization conditions, self-organized porous oxide structures can be grown on the alloy surface, (ii) SEM images revealed that the porous layers consist of arrays of single nanotubes with a diameter of 100 nm and a spacing of 150 nm, (iii) for the V-containing alloy, enhanced etching of the β-phase is observed, leading to selective dissolution and an inhomogeneous pore formation, and (iv) for the Nb-containing alloy an almost ideal coverage of both phases is obtained. According to XPS measurements, the tubes are a mixed oxide with an almost stoichiometric oxide composition, and can be grown to thicknesses of several hundreds of nanometers, suggesting that a simple surface treatment for Ti alloys has high potential for biomedical applications [435]. A vertically aligned nanotube array of titanium oxide was fabricated on the surface of titanium substrate by anodization. The nanotubes were then treated with NaOH solution to make them bioactive, and to induce growth of hydroxyapatite (bone-like calcium phosphate) in a simulated body fluid. It is found that (i) the presence of TiO_2_ nanotubes induces the growth of a “nano-inspired nanostructure”, *i.e.*, extremely fine-scale (∼8 nm feature) nanofibers of bioactive sodium titanate structure on the top edge of the ∼15 nm thick nanotube wall, (ii) during the subsequent *in vitro* immersion in a simulated body fluid, the nano-scale sodium titanate, in turn, induced the nucleation and growth nano-dimensioned hydroxyapatite phase, and (iii) such TiO_2_ nanotube arrays and associated nanostructures can be useful as a well-adhered bioactive surface layer on Ti implant metals for orthopedic and dental implants, as well as for photocatalysts and other sensor applications [436].

Webster *et al*. have suggested that enhanced vitronectin adsorption, conformation and bioactivity are the major reason for increased osteoblast adhesion on nanophase alumina [437]. In the last years, a new method has been described to fabricate nanotubular structures on titanium surfaces. These titania nanotubes can be produced by a variety of methods including electrochemical deposition, sol-gel method, hydrothermal processes and anodic oxidation [438–440]. Using this novel approach, several studies showed that the presence of the nanotube structure on a titanium surface induced a significant increase in the action of osteoblastic cells compared to those grown on flat titanium surfaces [441,442]. Nanotubular TiO_2_ layer produced using anodization has an amorphous crystal structure and it has been shown that using heat-treatment it can be transformed into anatase to improve cellular interactions [441]. In this study, a sintering protocol at 450 °C for 2 h was used to perform a crystal phase transformation of nanotubes. Significantly higher cell proliferation rates and better cellular morphologies were observed on anatase nanotubular surfaces after 7 days of culture, as shown in the literature [442]. Park *et al*. [443] produced nanotubular surfaces having pore diameter of 15, 20, 30, 50, 70 and 100 nm without heat treatment and documented that on nanotubular surfaces above 50 nm, the cell attachment and spreading was significantly decreased, thereby causing an increased programmed cell death. They only showed better cell proliferation and matrix mineralization results on nanotubes having 15 nm pore diameter. Whereas, in another study, Oh *et al*. [444] performed heat treatment following anodization and showed that anatase nanotubes having larger pore diameter (70 to 100 nm) MSCs elongate better and undergo selective differentiation into osteoblast-like cells compared to small nanotubes. In the present study, nanotubes having pore diameter of 70–100 nm were produced and impaired cellular functions on non heat-treated amorphous nanotubular surfaces were observed. However, on anatase nanotubular surfaces, significantly increased cell proliferation values were recorded after 7 days of culture, as shown in the literature [441,442,444]. Residual fluorine within the amorphous nanotubes following anodization might be the factor for this decreased cellular density, as stated in the literature [441]. Therefore, this study was able to show that heat treatment is essential for the production of nanotubular implant surfaces since it provides a more ideal oxide crystal structure for the spreading and proliferation of the cells.

Beside microtopographical features, surface wettability and surface free energy are also important parameters influencing cell attachment, proliferation and differentiation [445]. Bauer *et al*. [446] cultured rat mesenchymal stem cells on nanotubular titanium surfaces having different wettability characteristics and found an increased cell attachment on super-hydrophobic surfaces compared with super-hydrophilic ones. In the present study, samples having different wettability profiles were obtained following roughening, anodization and heat treatment procedures. After anodization and heat treatment, water contact angles decreased gradually. However, due to the changes in surface chemistries following treatments, no correlation was found between surface wettability and cellular functions. Further studies are needed to evaluate the effect of hydrophilicity and surface chemistry of titania nanotubes on protein adsorption and cell responses.

Three types of bioactive polymethylmethacrylate (PMMA)-based bone cement containing nano-sized titania (TiO_2_) particles were prepared, and their mechanical properties and osteoconductivity are evaluated by Goto *et al*. [447]. The three types of bioactive bone cement were un-silanized TiO_2_, 50 wt%, silanized TiO_2_ 50 wt%, and 60 wt% mixed to PMMA. Commercially available PMMA cement was used as a control. The cements were inserted into rat tibiae and allowed to solidify *in situ*. After 6 and 12 weeks, tibiae were removed for evaluation of osteoconductivity. It was reported that (i) bone cements using silanized TiO_2_ were directly apposed to bone, while un-silanized TiO_2_ cement and PMMA control were not, (ii) the osteoconduction of cement with 60 wt% of silanized TiO_2_ was significantly better than that of the other cements at each time interval, and (iii) the compressive strength of cement with 60 wt% of silanized TiO_2_ was equivalent to that of PMMA, indicating that cement with 60 wt% of TiO_2_ was a promising material for use as a bone substitute [447]. Since it is essential for the gap between the hydroxyapatite coated titanium and juxtaposed bone to be filled out with regenerated bone, promoting the functions of bone-forming cells is desired. In order to improve orthopedic implant performance, Sato *et al*. [448] synthesized nanocrystalline hydroxyapatite (HA) powders to coat titanium through a wet chemical process. The precipitated powders were either sintered at 1,100 °C for 1 h in order to produce microcrystalline size HA, or were treated hydrothermally at 200 °C for 20 h to produce nanocrystalline HA. These powders were then deposited onto titanium by a room temperature process. It was reported that (i) the chemical and physical properties of the original HA powders were retained when coated on titanium by the room temperature process, (ii) osteoblast adhesion increased on the nanocrystalline HA coatings compared to traditionally used plasma-sprayed HA coatings, (iii) greater amounts of calcium deposition by osteoblasts cultured on Y-doped nanocrystalline HA coatings were observed [448]. With a wide variety of applications, nanotechnology has attracted the attention of researchers as well as regulators and industrialists, including nanodrugs and drug delivery, prostheses and implants, and diagnostics and screening technologies. We can take advantages of availability of ultra-fine nanomicrostructures of solid metals, alloys, powder, fibers, or ceramics to fabricate superplastically formed products.

It is indispensable here to mention the minimally invasive dentistry (MID) and minimally invasive surgery (MIS). The MIS concept has been created to allow new thinking and a new approach to dentistry where restoration of a tooth becomes the last treatment decision rather than first consideration as at present. It provides a practical approach to caries preventive measures based on the notion of demineralization and remineralization in a micro-phase in order to retain healthy teeth. The medical model of MID is characterized by (1) reduction in cariogenic bacteria, (2) preventive measures, (3) remineralization of early enamel lesions, (4) minimum surgical intervention of cavitated lesions, and (5) repair of defective restorations [449]. At the same time, it is mentioned that MIS has several advantages: (1) since the surgical area is narrower, damage on surrounding soft tissue can be minimized, (2) post-operation pain can be minimized, (3) hospital time can be shortened, and (4) early rehabilitation can be initiated [450]. These MIs in both dentistry and medicine inevitably require precisely manufactured prostheses in micro-scale or even nano-scale. It is anticipated that the MI-based technologies, as well as MI-oriented technologies, will be advanced in the near future.

### 5.9. Bioengineering and Biomaterial-Integrated Implant System

With the aforementioned supportive technologies, surfaces of dental and orthopedic implants have been remarkably advanced. These applications can include not only ordinal implant system but also miniaturized implants, as well as customized implants. Dental implant therapy has been one of the most significant advances in dentistry in the past 25 years. The computer and medical worlds are both working hard to develop smaller and smaller components. Using a precise, controlled, minimally invasive surgical (MIS) technique, the mini dental implants (MDI) are placed into the jawbone. The heads of the implants protrude from the gum tissue and provide a strong, solid foundation for securing the dentures. It is a one-step procedure that involves minimally invasive surgery, no sutures, and none of the typical months of healing. Advantages associated with the MDI are (1) it can provide immediate stabilization of a dental prosthetic appliance after a minimally invasive procedure. (2) It can be used in cases where traditional implants are impractical, or when a different type of anchorage system is needed. (3) Healing time required for mini-implant placement is typically shorter than that associated with conventional 2-stage implant placement and the accompanying aggressive surgical procedure. According the clinical reports, a biometric analysis of 1,029 MDI min-implants, five months to eight years *in vivo* showed that the MDI mini-implant system can be implemented for long-term prosthesis stabilization, and delivers a consistent level of implant success [451].

In addition to the aforementioned miniature implants, an immediate loading, as well as customized implants, have been receiving attention recently. Conventionally, a dental implant patient is required to have two-stages of treatment consisting of two dental appointments five to six months apart. Recently, a single stage treatment has received attention. Placing an implant immediately or shortly after tooth extraction offers several advantages for the patient as well as for the clinician. These advantages include shorter treatment time, less bone sorption, fewer surgical sessions, easier definition of the implant position, and better opportunities for osseointegration because of the healing potential of the fresh extraction site [452–455]. Titanium bar (particularly the portions in direct contact to connective tissue and bony tissue) is machined to have the exact shape of the root portion of the extracted tooth of the patient. The expected outcome of this method is a perfect mechanical retention, and therefore an ideal osseointegration can be achieved. This is called a custom (or customized) implant, which is fabricated by the electro-discharge machining (EDM) technique.

Prefabricated dental implant can be further machined to replicate the extracted tooth and machined implant (whose shape is exactly same as the patient’s extracted tooth) can be prepared within a relatively short operation time and can be placed within one hour to the patient. Since the root shape of the placed implant is exactly same as the extracted tooth’s root form, the follow-up reaction including osseointegration is expected shorter and placed implant’s retention force can be achieved within a relatively short time.

Electron-beam machining (EBM) is a machining process where high-velocity electrons (in the range of 50 to 200 kV to accelerate electrons to 200,000 km/s) are directed toward a work piece, creating heat and vaporizing the material. Electromagnetic lenses are used to direct the electron beam, by means of deflection, into a vacuum. The electrons strike the top layer of the work piece, removing material, and then become trapped in some layer beneath the surface. Applications of this process are annealing, metal removal, and welding. EBM can be used for very accurate cutting of a wide variety of metals. Surface finish is better and kerf width is narrower than those for other thermal cutting processes. The process is similar to laser-beam machining, but because EBM requires a vacuum, it is not used as frequently as laser-beam machining [456]. In addition to the immediate placement of dental implants, another concept has been introduced. Techniques such as stereoscopic lithography and computer-assisted design and manufacture (CAD/CAM) have been successfully used with computer-numerized control milling to manufacture customized titanium implants for single-stage reconstruction of the maxilla, hemimandible, and dentition without the use of composite flap over after the removal of tumors [457]. Nishimura *et al*. [458] applied this concept to dental implants to fabricate the individual and splinted customized abutments for all restoration of implants in partially edentulous patients. It was claimed that complicated clinical problems such as angulation, alignment, and position can be solved. However, with this technique, the peri-implant soft tissues are allowed to heal 2 to 3 weeks, so that at least two dental appointments are required.

Many oral implant companies (about 25 companies are currently marketing 100 different dental implant systems) have recently launched new products with claimed unique, and sometimes bioactive surfaces [459,460]. The focus has shifted from surface roughness to surface chemistry and a combination of chemical manipulations on the porous structure. To properly explain the claims for new surfaces, it is essential to summarize current opinions on bone anchorage, with emphasis on the potentials for biochemical bonding. There were two ways of implant anchorage or retention: mechanical and bioactive [459,460].

Recent research has further redefined the retention means of dental implants into the terminology of osseointegration *versus* biointegration. When examining the interface at a higher magnification level, Sundgren *et al*. [30] showed that unimplanted Ti surfaces have a surface oxide (TiO_2_) with a thickness of about 35 nm. During an implantation period of eight years, the thickness of this layer was reported to increase by a factor of 10. Furthermore, calcium, phosphorous, and carbon were identified as components of the oxide layer, with the phosphorous strongly bound to oxygen, indicating the presence of phosphorous groups in the metal oxide layer. Many retrospective studies on retrieved implants, as well as clinical reports, confirm the aforementioned important evidence (1) surface titanium oxide film grows during the implantation period, and (2) calcium, phosphorous, carbon, hydroxyl ions, proteins, *etc*. are incorporated in an ever-growing surface oxide even inside the human biological environments [460,461]. Numerous *in vitro* studies, (e.g., [461]) on treated or untreated titanium surfaces were covered and to some extent were incorporated with Ca and P ions when such surfaces were immersed in SBF (simulated body fluid). Additionally, we know that bone and blood cells are rugophilia, hence in order not only to accommodate for the bone growth, but also to facilitate such cells adhesion and spreading, titanium surfaces need to be textured to accomplish and show appropriate roughness [462]. Furthermore, gradient functional concept (GFC) on materials and structures has been receiving special attention not only in industrial applications, but in dental as well as medical fields [462]. Particularly, when such structures and concepts are about to be applied to implants, its importance becomes more clinically crucial. For example, the majority of implant mass should be strong and tough, so that occlusal force can be smoothly transferred from the placed implant to the receiving hard tissue [462]. However, the surface needs to be engineered to exhibit some extent of roughness. From such macro-structural changes from bulk core to the porous case, again the structural integrity should be maintained. The GFC can also be applied for the purpose of having a chemical (compositional) gradient. Ca-, P-enrichment is not needed in the interior materials of the implants. Some other modifications related to chemical dressing or conditioning can also be utilized for achieving gradient functionality on chemical alternations on surfaces as well as near-surface zones [462].

### 5.10. Technology-Integrated Implant Systems

Any new type of implant (not only dental but also orthopedic applications) should possess a gradual function of mechanical and biological behaviors, so that mechanical compatibility and biological compatibility can be realized with s single implant system [462]. Since microtextured Ti surfaces [67,463,464] and/or porous Ti surfaces [465–467] promote fibroblast apposition and bone ingrowth, the extreme left side representing the solid Ti implant body should have gradually increased internal porosities toward to the case side which is in contact with vital hard/soft tissue. Accordingly, mechanical strength of this implant system decreases gradually from core to case, whereas biological activity increases from core to case side. Therefore, the mechanical compatibility can be completely achieved. Porosity-controlled surface zones can be fabricated by an electrochemical technique [468], polymeric sponge replication method [65], powder metallurgy technique, superplastic diffusion bonding method [469], or foamed metal structure technique [470].

Once the Ti implant is placed in hard tissue, TiO_2_ grows and increases its thickness [30,41,471–478], due to more oxygen availability inside the body fluid, as well as co-existence of superoxidant. It is very important to mention here that Ti is not in contact with the biological environment, but rather there is a gradual transition from the bulk Ti material, stoichiometric oxide (*i.e.*, TiO_2_), hydrated polarized oxide, adsorbed lipoproteins and glycolipids, portroglycnas, collagen filaments and bundles to cells [40]. Such gradient functional structure can be also fabricated in CpTi and microtextured polyethylene terephthalate (PET) system [479]. In addition, a gradient structural system of Ti and TiN was developed [480]. During HA coating, a gradient functional layer was successfully fabricated [214]. To promote these gradient functional (GF) and gradient structural (GS) transitions, there are many *in vivo*, as well as *in vitro*, evidences indicting that surface titanium oxide is incorporated with mineral ions, water and other constituents of biofluids [30,38,39,481]. Since a surface layer of TiO_2_ is negatively charged, the calcium ion attachment can be easily achieved [33,482]. Retrieved Ti implants showed that surface TiO_2_ was incorporated with Ca and P ions [483], while *in vitro* treatment of TiO_2_ in extracellular fluids or simulated body fluid (SBF) for prolonged periods of incubation time resulted in the incorporation of Ca, P, and S ions into TiO_2_ [30,38–41,218,471,481,484]. Without prolonged treatment, there are several methods proposed to relatively short-time incubation for incorporation of Ca and P ions. For example, TiO_2_ can be electrochemically treated in an electrolyte of a mixture of calcium acetate monohydrate and calcium glycerophosphate [145]. As a result of incorporation of Ca and P ions, bone-like hydroxyapatite can be formed in macro-scale [485] or nano-dimension [448]. Again for reducing the incubation time, bone-like hydroxyapatite crystals can be formed by treating the TiO_2_ surface with water and hydrogen plasma immersion ion implantation, followed by immersion in SBF [146], or by treating in hydrogen peroxide followed by SBF immersion [147], or immersion in SBF while treating the TiO_2_ surface with micro-arc oxidation and irradiation with UV light [171]. It is also known that P ions can be incorporated into TiO_2_ while it is immersed in the human serum [40].

Bony growth extreme surface zones should have a same roughness as the roughness of receiving hard tissue through micro-porous texturing techniques. This area can be structured using nanotube concepts [434–436]. Because this zone responds strongly to osseointegration, the structure, as well as the chemistry, should accommodate favorable osteoinductive reactions. Bone morphogenetic protein [385,486,487], and nano-apatite can be coated [488]. The zone may be treated by femtosecond laser machining [404] to build a micro-scale 3D scaffold which is structured inside the macro-porosities. Such scaffold can be made of biodegradable material (e.g., chitosan), which is incorporated with protein, Ca, P, apatite particles or other species possessing bone growth factors [483].

## 6. Conclusions

A dental implant system is a typical and excellent example of integrated product using multiple disciplines including surface science and technology, surface modification and surface physics and chemistry. The success and longevity of dental implants are strongly governed by surface characteristics. We have been reviewing approximately 500 relevant articles and found that there are certain factors that successful implants must possess to accommodate the ossteointegation. They are (i) biological compatibility not to be toxic to surrounding hard and soft tissues, (ii) mechanical compatibility to smooth transfer the stress between the placed implant root and receiving hard tissue, and (iii) morphological compatibility to accommodate the surface rugophilicity and promote bony cell growth [462].

**Figure 2.**
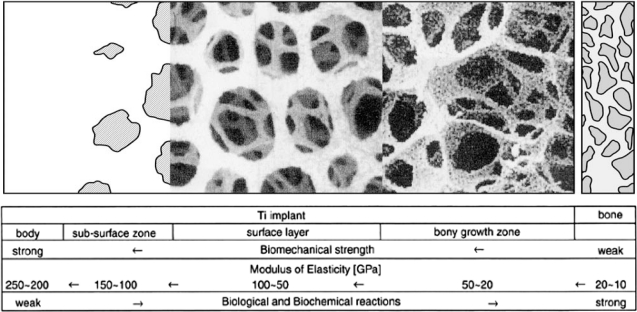
Schematic and conceptual Ti implant with gradient mechanical and biological functions.

Figure 2 illustrates a schematic and conceptual Ti implant that possesses a gradual function of mechanical and biological behaviors, so that mechanical compatibility and biological compatibility can be realized.

Some of ever-progressing manufacturing technologies as well as tissue engineering can be applied to fabricate dental implants. We are proposing a novel dental implant system, based on the gradual function design concept and aforementioned three major requirements for successful dental implant systems. Accordingly, new dental implant possesses two-fold function; (1) core portion of implant should solid and strong enough to bear biomechanical load and is gradually less strength with less modulus elasticity to fully accommodate the bony cell adaption at case portion of implant, and (2) such case portion’s characteristics could be suitable to growing hard tissue while core protion has still same function as those for adult patients.
